# Sequence analysis of the *Hsp70* family in moss and evaluation of their functions in abiotic stress responses

**DOI:** 10.1038/srep33650

**Published:** 2016-09-20

**Authors:** Ting Tang, Anmin Yu, Ping Li, Hong Yang, Gaojing Liu, Li Liu

**Affiliations:** 1 Key Laboratory for Plant Diversity and Biogeography of East Asia, Kunming Institute of Botany, Chinese Academy of Sciences, Kunming, 650201, China; 2 Key Laboratory of Economic Plants and Biotechnology, Kunming Institute of Botany, Chinese Academy of Sciences, Kunming, Yunnan 650201, China; 3 Yunnan Key Laboratory for Wild Plant Resources, Kunming, Yunnan 650201, China; 4 University of Chinese Academy of Sciences, Beijing, 100049, China

## Abstract

The 70-kD heat shock proteins (Hsp70s) are highly conserved molecular chaperones that play essential roles in cellular processes including abiotic stress responses. *Physcomitrella patens* serves as a representative of the first terrestrial plants and can recover from serious dehydration. To assess the possible relationship between *P. patens* Hsp70s and dehydration tolerance, we analyzed the *P. patens* genome and found at least 21 genes encoding Hsp70s. Gene structure and motif composition were relatively conserved in each subfamily. The intron-exon structure of *PpcpHsp70-2* was different from that of other *PpcpHsp70s*; this gene exhibits several forms of intron retention, indicating that introns may play important roles in regulating gene expression. We observed expansion of *Hsp70s* in *P. patens*, which may reflect adaptations related to development and dehydration tolerance, and results mainly from tandem and segmental duplications. Expression profiles of rice, *Arabidopsis* and *P. patens Hsp70* genes revealed that more than half of the *Hsp70* genes were responsive to ABA, salt and drought. The presence of overrepresented *cis*-elements (DOFCOREZM and GCCCORE) among stress-responsive *Hsp70s* suggests that they share a common regulatory pathway. Moss plants overexpressing *PpcpHsp70-2* showed salt and dehydration tolerance, further supporting a role in adaptation to land. This work highlights directions for future functional analyses of *Hsp70s.*

Plants cannot move to avoid harm, and have evolved a wide array of mechanisms to adapt to stressful environments. Heat shock proteins (HSPs) are stress proteins that were initially identified as responsive to heat stress^1^. HSPs serve as pivotal molecular chaperones by preventing aggregation of denatured proteins and promoting opportune protein folding under heat stress^2^,^3^,^4^. According to their approximate molecular weight, HSPs have been classified into five families: Hsp100, Hsp90, Hsp70, Hsp60 and small (s)Hsp^5^. Among them, Hsp70 (also known as DnaK-like) superfamily members together with their co-chaperone GrpE and Hsp40 (DnaJ-like) proteins, form a system for protein folding, degradation, and transport processes throughout the cell^6^. They also play essential roles in photosynthesis, signal transduction, transcriptional activation, and abiotic stress responses^7^,^8^. Structurally, Hsp70s are characterized by three distinct domains: an N-terminal adenosine triphosphatase (ATPase) domain, a substrate-binding domain, and a highly variable C-terminal domain.

Photosynthetic eukaryotes possess at least four types of Hsp70s, each of which localizes to a different cellular compartment: cytoplasm, mitochondrion (MT), chloroplast (CP) and endoplasmic reticulum (ER)^9^. The Hsp70s targeted to a given subcellular compartment share a similar evolutionary history. The ER and cytoplasmic Hsp70s evolved by gene duplication and subsequent divergence, whereas the MT and CP Hsp70s evolved by gene transfer from the endosymbiont to the nucleus^10^. In *Arabidopsis*, at least 18 Hsp70 superfamily members have been identified, and the genes show distinct expression profiles during different developmental stages and under thermal stress^11^. Moreover, the Hsp70s in peanut (*Arachis hypogaea* L.) have been confirmed play an important role in conferring drought tolerance^12^. Stromal Hsp70 chaperones also play a key role in chloroplast protein import^13^,^14^.

The model plant *P. patens* is dehydration tolerant and is able to recover upon rehydration, even after dehydration leading to 92% loss of fresh weight^15^. This ability of *P. patens* to tolerate severe dehydration makes it an ideal candidate for elucidating the molecular mechanisms by which plants respond to dehydration stress. *P. patens* is also an excellent model plant for studying plant physiology and development due to its amenability to gene knockout and allele replacement by homologous recombination^16^. Additionally, *P. patens* occupies a key position evolutionarily, serving as a link between green algae and seed plants^17^. *P. patens* can thus be used as a bryophyte representative of the first terrestrial plants^18^. As protective proteins, Hsp70s in *P. patens* have attracted attention for their potential roles in the process of adaptation to land, which would necessitate mechanisms for protection against stresses related to changes in temperature, light, and water availability^19^.

In this study, we identified *Hsp70* homologs in *P. patens*, analyzed the trends of *Hsp70* evolution among species, and examined the expression patterns of *Hsp70* genes during different developmental stages and during abiotic stress treatment. Analysis of overrepresented *cis*-elements among clusters was performed to identify conserved motifs potentially responsible for specific regulatory pathways. Furthermore, we prepared moss *cpHsp70-2* overexpression lines and found that they showed salt and dehydration tolerance. Our results lay the foundation for functional analysis of the roles of Hsp70s in stress tolerance of *P. patens*.

## Results

### Phylogenetic Analyses of the Hsp70 Superfamily

To identify putative *P. patens Hsp70* genes, we first searched Phytozome databases using a published *Arabidopsis* Hsp70 protein with conserved domain sequences as query; 29 genes were obtained using a maximum E-value of 1e-5 (Supplementary Table S1). Domain searches in SMART (http://smart.embl-heidelberg.de/smart) identified the Hsp70 domain in 21 of the corresponding predicted protein sequences. It was previously reported that 24, 18 and 20 *Hsp70* genes are present in rice, *Arabidopsis* and poplar, respectively. *Escherichia coli* has three *Hsp70* genes: *DnaK*, *HscA* and *HscC*, whereas *Saccharomyces cerevisiae* contains 12 family members. In contrast to algal genomes, which have only a single cytosolic *Hsp70*, *P. patens* and other land plants have apparently required the expansion of protective proteins over the course of evolution^20^. The *Hsp70* genes identified in *P. patens* encode proteins ranging from 509 to 714 amino acids (aa) in length. Detailed information on the *Hsp70* genes in *P. patens*, including their gene IDs and the characteristics of their encoded proteins are listed in Supplementary Table S2.

**Figure 1:**
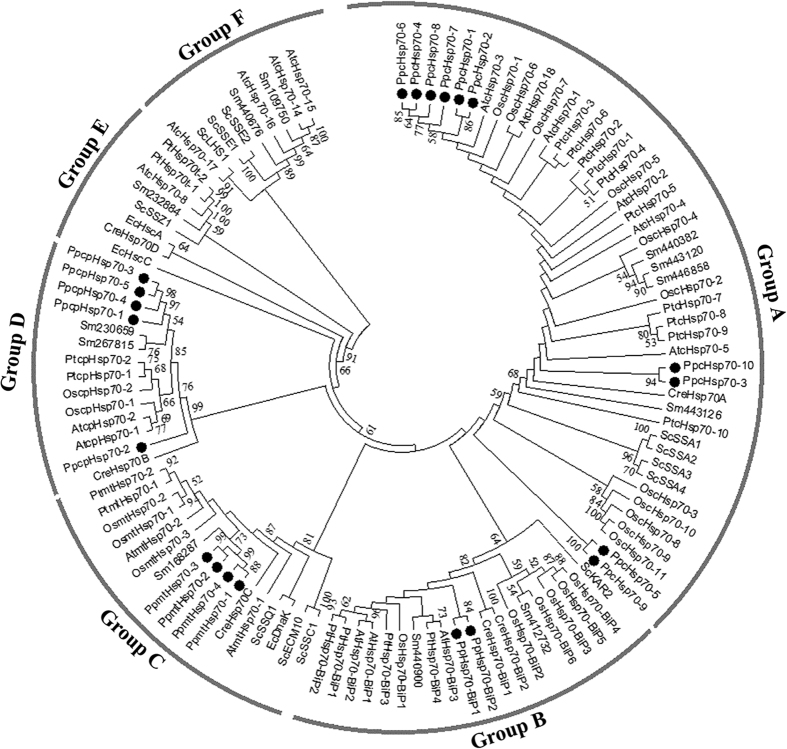
Phylogenetic tree of Hsp70 superfamily in eight species. The tree was constructed using the Neighbor-Joining (NJ) method based on the amino acid sequences of Hsp70 members from *Escherichia coli* (Ec), *Saccharomyces cerevisiae* (Sc), *Chlamydomonas reinhardtii* (Cr), *Physcomitrella patens* (Pp), *Selaginella moellendorffii* (Sm), *Oryza sativa* (Os), *Arabidopsis thaliana* (At), and *Populus trichocarpa* (Pt). The Hsp70s were classified into six groups, Group A localized in the cytoplasm, Group B localized in the ER (endoplasmic reticulum), Group C localized in the mitochondrion, Group D localized in the chloroplast according to the phylogenetic analyses, Group E comprised truncated genes, and Group F was a Hsp110/SSE subfamily. The 21 Hsp70 proteins of the *P. patens* were marked with black dots, and were classified into 4 groups. Numbers at each branch indicate the percentage support for the node among 1,000 bootstrap replicates.

To investigate the evolutionary relationships among *Hsp70s* from different species including both eukaryotes and prokaryotes, we identified *Hsp70* genes from *E. coli*, *S. cerevisiae*, *C. reinhardtii*, *S. moellendorffii*, *O. sativa*, *Arabidopsis*, and *P. trichocarpa* (Table 1). A Neighbour-joining (NJ) phylogenetic tree was generated by aligning the full-length Hsp70 protein sequences from these species. The Hsp70s were classified into six major groups (from group A to F), each appearing to correspond to a particular predicted subcellular localization, except groups E and F (Fig. 1). Group A was the largest subfamily, with 45 members including ten members with moss paralogs. This group comprised members localized in the cytoplasm, distributed from *E. coli* to *P. trichocarpa*. Group B had 20 members, which were predicted to be localized in the ER lumen based on the *Arabidopsis* and rice orthologs. Two moss Hsp70s belonged to the same group as BiP.KAR2 in the yeast *S. cerevisiae*. Group C had 17 members, consisting of proteins likely found in the mitochondrion matrix, with four moss proteins forming a divergent group. Group D consisted of 14 members with five moss paralogs in the plastid stroma, and *C. reinhardtii* Hsp70B was the basal member of this group. Group E comprised five members that perhaps represented truncated genes, based on *P. trichocarpa* paralogs^21^. Group F formed an Hsp110/SSE subfamily, members of which are similar to Hsp70 in structure and belong to the Hsp70 superfamily. No moss proteins were included in Groups E and F.

The Predotar and LocTree3 tools predicted that moss PpcHsp70-1 to PpcHsp70-10 localize to the cytoplasm. PpHsp70-BiP1 and PpHsp70-BiP2 were predicted binding proteins (BiPs) localizing in the ER. Integration analysis of the phylogenetic tree and subcellular localization predictions indicates that the moss Hsp70 proteins also function in different compartments like other land plants. Specifically, Group A members are likely to function preferentially in the cytoplasm, Group B in the ER, Group C in the mitochondrion matrix, and Group D in plastid stroma. In *P. patens*, the *Hsp70* superfamily genes encode 10 cytosolic Hsp70s, 2 BiPs, 5 plastid Hsp70s (cpHsp70s), 4 mitochondrial Hsp70s (mtHsp70s). In general, the relationships displayed in the phylogenic tree were consistent with the traditional taxonomic classification, while the number of chloroplast Hsp70s varied a lot.

### Multiple Sequence Alignment, Domain, and Gene Structure Analysis of *Hsp70* Family Members in *P. patens*

**Figure 2:**
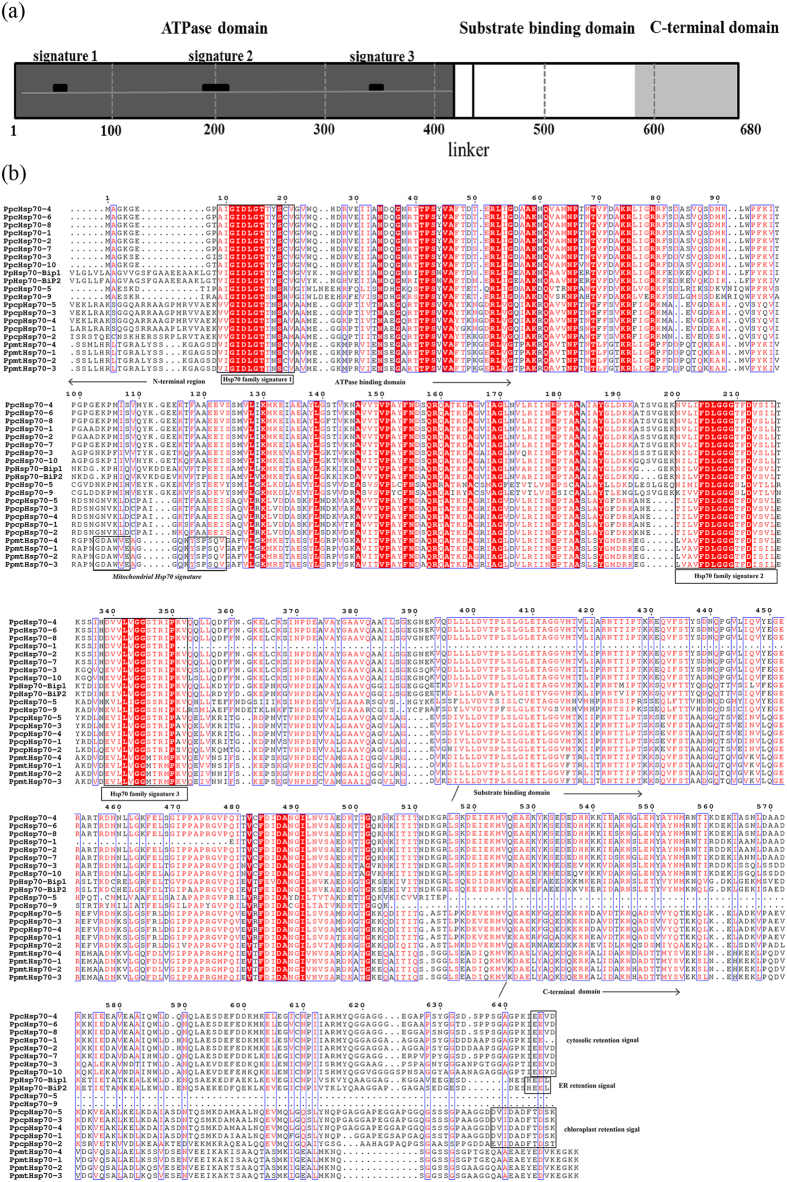
Analysis of conserved domains in Hsp70 superfamily proteins from *P. patens*. (**a**) PpmtHsp70-1 was shown as representative example of the domain structure of Hsp70 proteins, including the ATPase domain (1–400 aa, dark gray box, containing three typical signature motifs), the substrate-binding domain (437–579 aa, white box) and the C-terminal domain (580–680 aa, light gray box). (**b**) Multiple sequence alignment of Hsp70 proteins. The amino acid sequences of PpHsp70s are numbered on the left. In the ATPase domain, the three typical Hsp70 signature motifs are highlighted and boxed. In the C-terminal domain, the C-terminus specific signature motifs are boxed. The sequence from 220 aa to 330 aa is instead marked by *dots* to indicate conservation.

**Figure 3:**
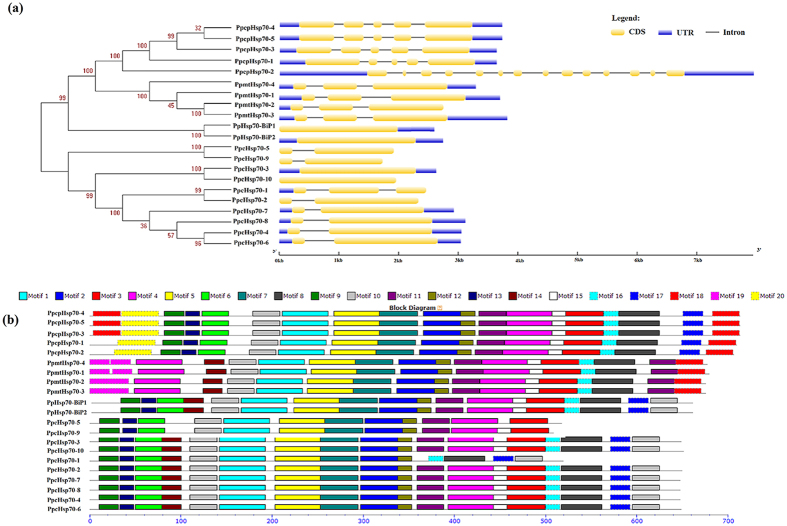
Phylogenetic relationships, gene structures, and motif compositions of Hsp70 superfamily members in *P. patens*. (**a**) Multiple sequence alignment of Hsp70s from *P. patens* was performed using MEGA 6.06 by the NJ method with 1,000 bootstrap replicates (left panel). In the right panel, intron-exon structures of the *Hsp70* genes are shown. Yellow boxes represent exons, black lines represent introns, and blue boxes represent UTR (Untranslated Regions). (**b**) A schematic representation of conserved motifs were presented in Hsp70 superfamily proteins. Motifs were identified by MEME software using complete amino acid sequences of Hsp70 proteins. Different motifs are represented by different colored boxes. Details of the individual motifs are in Supplementary Table S3. The protein sequences are arranged in the order shown in the NJ tree.

Multiple sequence alignment showed that Hsp70s in moss were similar to Hsp70s of other species, and included three distinct domains (Fig. 2a). The highly conserved N-terminal adenosine triphosphatase (ATPase) domain (approximately 400 amino acids) contained three Hsp70 signature sequences in groups A, B, C and D. The substrate-binding domain (approximately 200 amino acids) was extremely well conserved, but the C-terminal domain was highly variable (Fig. 2b). Most of the moss predicted Hsp70 proteins contained these domains. However, cHsp70-1 lacked part of the N terminal-ATPase domain and the substrate-binding domain. In addition, cHsp70-5 and cHsp70-9 did not include the C-terminal domain. We next used the Hsp70 proteins from *P. patens* to construct a phylogenetic tree by Mega 6.06 (Fig. 3a, left). In the cytoplasmic group, a conserved retention signal EEVD was found at the C terminus, except in cHsp70-5 and cHsp70-9. The mitochondrial Hsp70 homologs possessed the conserved signature sequences GDAWV and YSPSQI. Interestingly, whereas Hsp70-Bip1 and Hsp70-Bip2 were homologous to ER proteins, they had HED/EL sequences at the C terminus, in contrast to the HDEL ER retention signal of Hsp70 in other land plants. The motif for five chloroplast Hsp70s was conserved (DVIDADFTDSK), while the plastid signal of cpHsp70-2 was slightly different from that of other four cpHsp70s.

Using MEME, 20 consensus motifs were detected in moss Hsp70 proteins, with lengths ranging from 15–50 aa (Fig. 3b, and Supplementary Table S3). Most Hsp70s contained motifs 1, 2, 4, 5, 7, 10, 11, and 12, which corresponded to the conserved ATPase domain. Motifs 3, 8, 15 and 16 were included in the substrate peptide domain. Motif 20 was uniquely found in the chloroplast group, whereas motif 14 was absent from those same proteins. The conserved ATPase domain of the mitochondrion group lacked motifs 9 and 13, but contained motif 19. Motif 4 was repeated close to the C-terminus of the mitochondrial Hsp70s, but motif 17 was absent in those proteins. The ER and cytosolic groups were almost identical in motif type and order.

To gain further insights into the structural diversity of *Hsp70* genes in *P. patens*, we compared the intron-exon organization in the coding sequences between individual *Hsp70* genes of *P. patens* (Fig. 3a, right). Most closely related members in the same subfamily shared similar intron number and exon length. In the chloroplast group, *Hsp70* genes had four introns except *cpHsp70-2*, which had 13 introns. The genes for mitochondrion-localized *Hsp70s* had two introns, and genes encoding ER-localized BiPs had zero or one intron, while most genes for cytosolic Hsp70s had one or two introns, although *cHsp70-3* and *cHsp70-10* had no introns. These patterns are highly suggestive of a duplication-mediated origin for these genes. Interestingly, knock-out analysis of chloroplast *Hsp70s* in *P. patens* has showed that only *PpcpHsp70-2* is vital for moss development, with the other four genes showing redundancy^14^. This observation combined with our results indicates introns may play important roles in *PpcpHsp70-2* function, consistent with the role of alternative splicing events that increase the diversity of gene function^22^. Accordingly, we analyzed the RNA-seq database in Phytozome and other reported data, and found that only *PpcpHsp70-2* showed evidence of intron retention event^23^,^24^. Thus, analysis of the function of *PpcpHsp70-2* introns appears to be a potentially fruitful topic for further research.

### Gene Duplication is the Main Factor Increasing the Diversity of *Hsp70* Genes in *P. patens*

**Figure 4:**
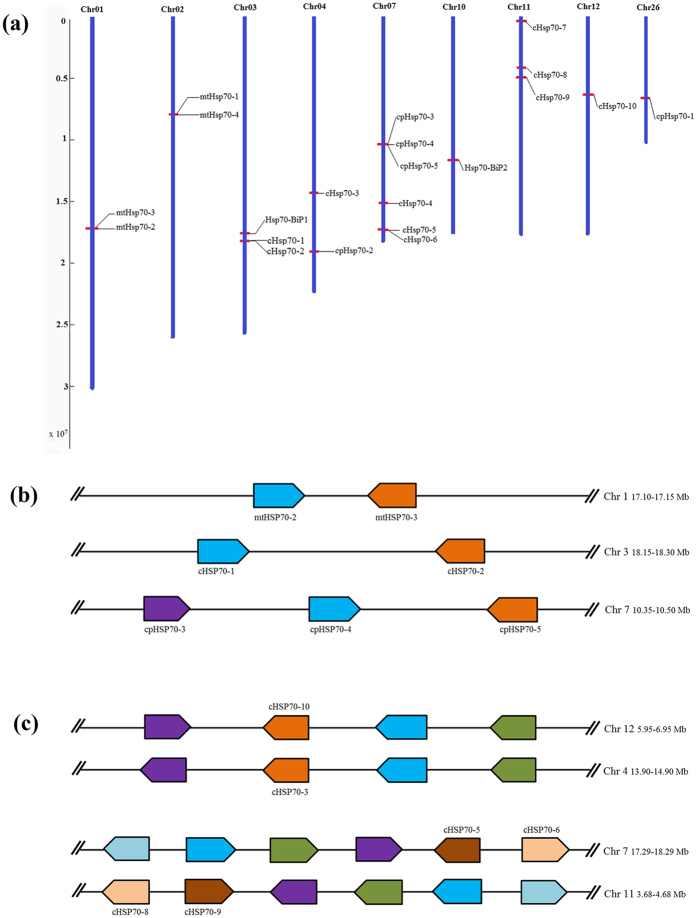
Chromosomal locations and gene duplications of *P. patens Hsp70s*. (**a**) The 21 *Hsp70* genes were mapped to 9 chromosomes. Schematic diagram of *P. patens Hsp70s* based on the sequence map was provided by the Phytozome website. Gene names are listed to the left of the chromosomes, and map markers are listed to the right. (**b**) Evidence for tandem duplication of *P. patens Hsp70s*. Diagram shows chromosomal locations of *Hsp70* genes and linked homologous genes in *P. patens* identified in PTGBase (http://ocri-genomics.org/PTGBase/). Pentagons point in the 5′→3′ direction. (**c**) Evidence for segmental duplication of *P. patens Hsp70s*. Paralogous gene pairs generated by gene duplication within the *Hsp70* family of *P. patens* were analyzed using the Plant Genome Duplication Database (http://chibba.agtec.uga.edu/duplication/). The black line represents syntenic blocks in *P. patens* chromosomes, and the different colors of pentagons represent different genes. The *Hsp70* gene names are marked above or below the pentagons. Synonymous (Ks) and nonsynonymous substitution (Ka) rates are presented for each pair. Gene pairs were generated by tandem duplication (T) and whole-genome duplication (W).

To investigate further the evolution of *Hsp70* genes in *P. patens*, we examined the chromosomal locations of the identified *PpHsp70* genes. The 21 genes were distributed on 9 chromosomes, with 3 genes (*Hsp70-BiP2*, *cHsp70-10*, *cpHsp70-1*) localized alone on Chr 10, 12, 26, respectively (Fig. 4a). The distributions of *Hsp70* genes among the *P. patens* chromosomes appeared to be uneven: Chr 1, 2, 3, 4 and 11 each contained two or three *Hsp70* genes, while relatively high densities were presented on Chr 7.

Segmental and tandem duplication has played a crucial role in the evolution and expansion of gene families in plants^25^. As shown in Fig. 4b, four pairs of *PpHsp70* genes were tandemly duplicated on Chr 1, 3 and 7. For example, *mtHsp70-2* and *mtHsp70-3* were found in tail-to-tail orientation on Chr1. Similarly, *cHsp70-1* and *cHsp70-2* were linked tail-to-tail on Chr3. These genes likely arose from local gene duplication. Likewise, *cpHsp70-3* and *cpHsp70-4* were tandemly arranged in head-to-tail orientation, and *cpHsp70-4* was linked tail-to-tail with *cpHsp70-5* on Chr7. In addition, segmental duplication was observed among 6 genes forming 3 groups (Fig. 4c) on Chr 12, 4, 7 and 11. Synteny involving *cHsp70-3* and *cHsp70-10* linked homologues of two other genes on Chr 4 and Chr12, respectively. *cHsp70-5* on Chr 7 was also linked segmentally to *cHsp70-9* on Chr 11. We further identified large blocks of synteny between Chr7 and 11 (including *cHsp70-5*/*cHsp70-9*, *cHsp70-6*/*cHsp70-8* and six other paired genes) by investigating the flanking regions within 500 kb in either direction of *Hsp70* genes. The synonymous substitution rate (Ks) values of three paired genes (*cHsp70-3*/*cHsp70-10*, *cHsp70-5*/*cHsp70-9*, *cHsp70-6*/*cHsp70-8*) ranged from 0.7 to 1.4, and the ratios of the nonsynonymous substitution rate (Ka) to the synonymous substitution rate (Ks) were 0.21, 0.01 and 0.05, respectively (Table 2).

### *Hsp70* Genes in Land Plants are Responsive to ABA, Salt or Drought

**Figure 5:**
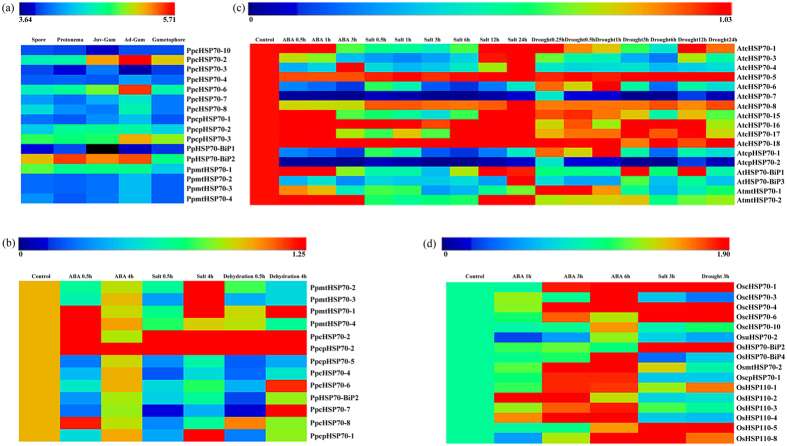
*Hsp70* expression profiles for *P. patens, O. sativa*, and *Arabidopsis* are shown. The *Arabidopsis* microarray gene expression data were obtained from AtGenExpress. The public expression data in rice were obtained from the Michigan State University (MSU) Rice Genome Annotation (http://rice.plantbiology.msu.edu) databases. The *P. patens* transcriptome data were obtained from Phytozome 10.3 (http://phytozome.jgi.doe.gov/pz/portal.html). (**a**) The heat map shows expression of *Hsp70* genes in different developmental stages (spore, protonema, juvenile stage, adult stage and gametophore) according to available microarray-based data. The expression profile was generated with log-transformed average values (**b**) *P. patens Hsp70* superfamily genes expression under ABA (0.5 h and 4 h), salt (0.5 h and 4 h) and dehydration treatment (0.5 h and 4 h). (**c**) *Arabidopsis Hsp70* superfamily genes expression under, ABA (0.5 h, 1 h and 3 h), salt (0.5 h, 1h, 3 h, 6 h, 12 h and 24 h), drought treatment (0.25 h, 0.5 h, 1h, 3 h, 6 h, 12 h and 24 h). (**d**) Rice *Hsp70* superfamily genes expression under ABA (1 h, 3 h and 6 h), salt (3 h), and drought (3 h) treatment. The expression profile of (**b**–**d)** was generated with the fold changes using the average values for each treatment divided by the values of the control.

We obtained publicly available microarray data to explore the expression profiles of *Hsp70* genes at different stages of development and under abiotic stress treatments in moss, rice and *Arabidopsis*. As Fig. 5a and Supplementary Table S4 show, the expression of two cytoplasm-group genes (*cHsp70-2*, *cHsp70-6*), and two chloroplast-group genes (*cpHsp70-2* and *cpHsp70-3*) in *P. patens* increased gradually with developmental stage from spore to protonema through adult gametophore, suggesting that these genes involved in the development of moss. Other genes maintained a steady expression level throughout multiple stages of development. For example, a gene encoding a homolog of the binding proteins in the ER (*Hsp70-BiP1*) showed high expression in both protonema and the adult gametophore. In *P. patens, cHsp70-2* and *cpHsp70-2* were highly induced by ABA, salt and dehydration treatment (4–11 fold increase) and maintained high expression as the time of treatments continued. The expression of other *Hsp70* genes in *P. patens* decreased significantly except for *mtHsp70-1*, *mtHsp70-4* and *cpHsp70-8*, which was initially induced dramatically by salt and then dropped rapidly. In rice, more than half of the *Hsp70* genes were highly induced by ABA, salt and dehydration treatments, whereas *OscpHSP70-1* expression was only slightly induced by ABA treatment. By contrast, most *Arabidopsis Hsp70* genes, such as chloroplastic *AtcpHsp1* and *AtcpHsp2*, exhibited a pattern of a decreased expression, with slight quantitative differences. Among all chloroplast *Hsp70* genes, only moss *cpHsp70* was up-regulated highly and steadily under ABA, salt and dehydration treatment (none were up-regulated in rice or *Arabidopsis*), suggesting that moss *cpHsp70s* might have been critical for the adaptation to land.

**Figure 6:**
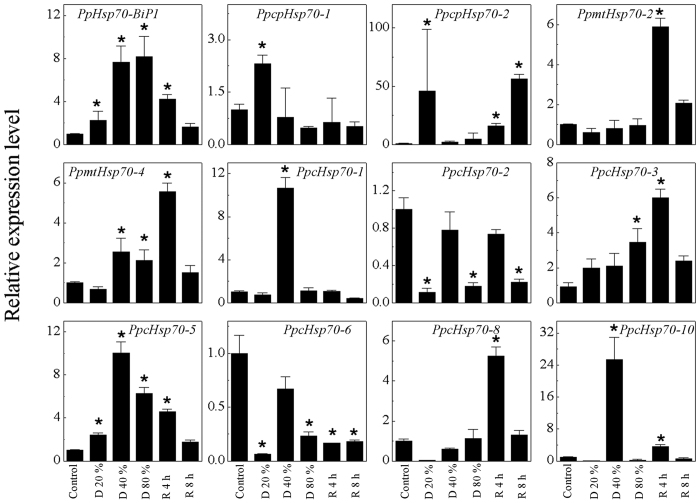
Relative normalized expression of *P. patens Hsp70* superfamily genes during treatment with dehydration stress and rehydration. The line-chart shows relative expression of *Hsp70* genes at different points during treatment with dehydration stress and rehydration, as monitored by RT-qPCR (with *Actin* as control). Control, *P. patens* gametophores with no treatment; D 20%, *P. patens* gametophores air-dried to 20% water loss; D 40%, *P. patens* gametophores air-dried to 40% water loss; D 80%, *P. patens* gametophores air-dried to 80% water loss; R 4 h, D 80% *P. patens* gametophores re-watered for 4 h; R 8 h, D 80% *P. patens* gametophores re-watered for 8 h. There were five replicates for each treatment, and the experiment repeated at least three times. Values are mean ± S.D, *n* = 5. An asterisk indicates that the value of treatment is different from control (*p* < 0.05).

To further explore the *P. patens Hsp70* response to dehydration stress and rehydration, we used qRT-PCR to determine relative normalized expression (Fig. 6). At the beginning of dehydration treatment, decreased expression was observed for most of the *P. patens Hsp70* genes, e.g., *mtHsp70-2*, *mtHsp70-4*, *cHsp70-2*, *cHsp70-6*, *cHsp70-8*, and *cHsp70-10*. By contrast, at the beginning of rehydration most *Hsp70* gene expression increased, as for *cpHsp70-2*, *mtHsp70-2*, *mtHsp70-4*, *cHsp70-3*, *cHsp70-5*, *cHsp70-8*, and *cHsp70-10*. However, *cHsp70-1* transcripts accumulation increased 10-fold after dehydration treatment leading to 40% water loss, and those of *cpHsp70-2* increased 46-fold after dehydration to 20% water loss. Thus, the *Hsp70* expression patterns during dehydration and rehydration reveal these genes to be quite sensitive to such stress in *P. patens*. Overall, these results indicate the likely function of moss *Hsp70* superfamily genes, especially chloroplastic *cpHsp70-2*, in abiotic stress defense.

### *Cis*-element Analysis of *Hsp70* Promoter Sequences Points to Conserved Regulatory Pathways

**Figure 7:**
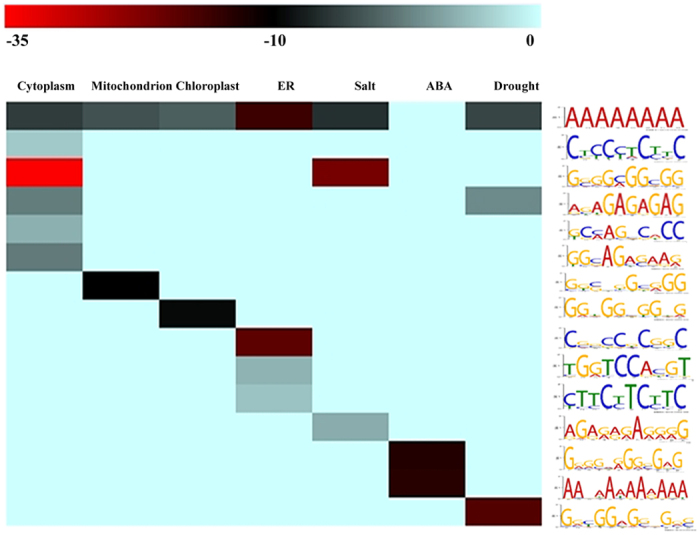
*Cis*-element analysis of promoter sequences of genes for *Hsp70s* localized to different cellular locations and induced under different abiotic stress treatments. Over-representation of known *cis*-elements in promoters of *Hsp70* superfamily genes was extracted according to the E-value. Logo representations of known *cis*-elements are on the vertical axis, and the different cellular locations and treatments are on the horizontal axis. Colored boxes represent log 10-transformed average E-value of *cis*-element and cellular locations and treatment with a significant statistical link.

To explore the evolution of the regulation of *Hsp70* genes in land plants, we performed a comprehensive *cis-*element analysis for seven clusters of *Hsp70* genes: cytoplasm localized, mitochondrion localized, chloroplast localized, ER localized, salt induced, ABA induced, drought or dehydration induced (for details of clusters see Supplementary Tables S5 and S6). Certain *cis*-elements were selectively enriched in various clusters (Fig. 7 and Supplemental Table S6), although there was an obvious difference in the range of *cis*-elements distributed among the different clusters. Seven clusters were enriched for the MARTBOX, which is the most common element in flowering plants and is suggested to play role in transcriptional regulation^26^. The SORLIP2AT element, which is over-represented in light-induced promoters of phytochrome genes (phyA) in *Arabidopsis*^27^, was significantly enriched in cluster 3. Another element, DOFCOREZM, associated with plant metabolism and drought responses^28^,^29^, was overrepresented in cluster 6. The GCCCORE was enriched in cluster 5 and is reported to be present in promoters of many pathogenesis-related genes with a role in JA signaling pathways or plant defense signal perception^30^,^31^. A novel element (GGCGGAGGGGGG) was prominently enriched in cluster 7, with E-value 7.6E-19. These results suggest that clusters of *Hsp70* genes share common regulatory factors and indicate conservation of elements in the evolution of stress regulatory networks in land plants.

### Moss Plants Overexpressing *PpcpHsp70-2* Show Salt and Dehydration Tolerance

**Figure 8:**
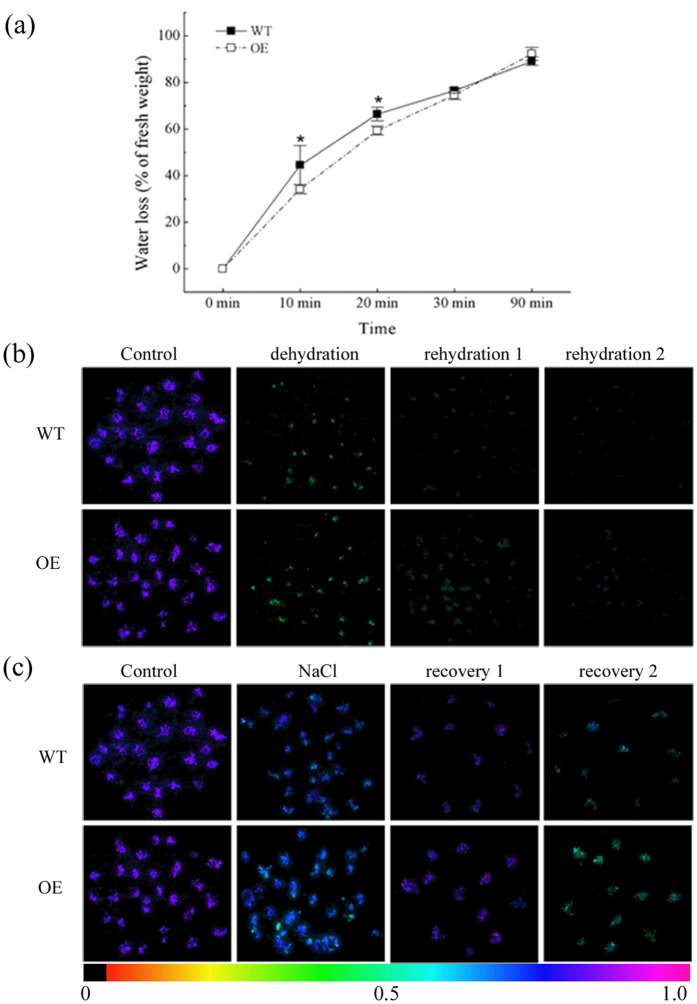
Overexpression *PpcpHsp70-2* plants showed salt and dehydration tolerance. (**a**) Time courses of water loss from gametophores of wild-type (WT) and over-expression *PpcpHsp70-2* (OE) plants. Water loss was calculated as the percentage of initial fresh weight. (**b**) Chlorophyll florescence of wild-type and overexpression plants during the course of dehydration and rehydration. *P. patens* gametophores air-dried to 80% water loss (dehydration) and then re-watered for 1 d (rehydration 1) and 2 d (rehydration 2) at room temperature. (**c**) Chlorophyll florescence of wild-type and overexpression plants after NaCl treatment and recovery at normal growth conditions. *P. patens* gametophores were treated on plates with 500 mM NaCl for 3 d and then transferred to normal conditions for recovery periods of 1 d (recovery 1) and 2 d (recovery 2). There were five replicates for each treatment, and the experiment repeated at least three times. Values are mean ± S.D, *n* = 5. An asterisk indicates that the value of treatment is different from control (*p* < 0.05).

To address the question of whether *PpcpHsp70-2* plays important roles in abiotic stress responses *in vivo*, transgenic moss overexpressing *PpcpHsp70-2* under the control of the CaMV35S promoter was generated and tested for salt and dehydration responses. These plants exhibited clearly increased tolerance of salt and dehydration, relative to WT plants (Fig. 8). Chlorophyll fluorescence of two overexpression lines and WT grew weaker during the salt and dehydration treatment, but WT lost photosynthetic activity faster than did the transgenic lines (Fig. 8b,c). These data support the conclusion that *PpcpHsp70-2* exerts a function not only in protein import but also in abiotic stress defense.

## Discussion

### The Hsp70 Superfamily in *P. patens*

Hsp70 proteins exist widely and play significant roles in organisms ranging from prokaryotes to the land plants. In this work, we aimed to characterize *Hsp70* genes in *P. patens* because Hsp70 proteins occupy a central position in the cellular chaperone network, interacting with chaperones of other families. To elucidate the evolutionary relationships between Hsp70 proteins in moss and other organisms, a combined phylogenetic tree was produced. Our phylogenetic analysis revealed that the Hsp70 family includes many paralogous genes with different functions, according to the six major clades displayed in the tree. Group E and F were divided earliest and the genes of these two groups, which were distributed broadly in many species, might be non-functional. Subsequently, Group D diverged and a large number of chloroplast Hsp70 proteins of different green plants were clustered in the same large clade, which suggests a common ancestry of plastid Hsp70 in diverse land plants. In particular, *PpcpHsp70-2* exhibited a distance from other *PpcpHsp70s* and was placed close to *CreHsp70-2*, which suggests this moss chloroplast *Hsp70* might have evolved from that in green algae. In general, the fact that *Hsp70* genes from various species were fell into the same large groups according to their predicted cellular locations, indicative of evolutionary conservation among organisms.

We identified 21 genes in the moss genome encoding the domains characteristic of Hsp70 proteins. For example, PpmtHsp70-1 contained an N-terminal ATPase domain, a substrate-binding domain, and a C-terminal domain and shared three typical signature motifs (Fig. 2), which coincide with the structural characteristics of the Hsp70 superfamily^9^. Though there have been studies on the cytosolic and chloroplast Hsp70 families of moss^5^,^14^, this work represents the first comprehensive study of the entire moss *Hsp70* superfamily.

We found four mtHsp70 proteins in moss, one more than in rice^32^. PpmtHsp70s possessed the conserved signature sequences, suggesting that they are ture mitochondrial Hsp70 homologs^33^. The mRNA level of a mitochondrial *Hsp70* gene from the Antarctic moss *Pohlia nutans* was peviously reported to increase after water deprivation and continually increase after re-watering^34^. Interestingly, we found a similar expression pattern for *mtHsp70-2* and *Hsp70-BiP2* in *P. patens* during dehydration and rehydration. In addition, there are two Hsp70-BiPs in both moss and *S. moellendorffii*, fewer than in rice and *Arabidopsis*. As demonstrated in tobacco, constitutive overexpression of Hsp70-BiPs is enough to confer tolerance to water stress^35^. The evolutionary similarity of the moss mtHsp70 and Hsp70-BiPs to those of the flowering plants suggests that the moss Hsp70 proteins might also share the common localization and function.

We identified 10 cytosolic *Hsp70* genes in moss, one more than previously reported^19^. Among these were genes for seven canonical proteins (including the EEVD at C terminus) and three nonclassical cytosolic Hsp70 proteins (cHsp70-1, cHsp70-5 and cHsp70-9). It has been reported that EEVD sequences are involved in binding proteins such as Hop (Hsp70 Hsp90 organizing protein) through tetratricopeptide motifs^36^. The seven canonical cytosolic Hsp70 proteins might play similar important roles in moss as in other land plants. By contrast, we did not find evidence for expression of the nonclassical *Hsp70* genes in the profiling data reported by Hiss^37^, which provides evidence that *cHsp70-1*, *cHsp70-5* and *cHsp70-9* genes probably are pseudogenes (Fig. 4). The function and impact of these pseudogenes in moss remains to explore in the future.

Shi *et al.*^14^ previously described three cpHsp70 proteins, and we found genes corresponding to five cpHsp70 proteins in our study, thanks to improved genome sequencing information. Although cpHsp70-3, cpHsp70-4 and cpHsp70-5 had the same predicted length, pI and molecular weight, their corresponding genomic positions were different. Compared with other plant species (Fig. 1), moss had more cpHsp70 proteins, suggesting that they might have been important in adaptation to the land environment. PpcpHsp70-1, PpcpHsp70-3, PpcpHsp70-4 and PpcpHsp70-5 were clustered into a separate clade and formed a sister group with two predicted proteins of *S. moellendorffii*, whereas PpcpHsp70-2 was placed in a sister group with Hsp70B of *C. reinhardtii*. Knockout of *PpcpHsp70-2* is lethal^14^, and considering the difference in plastid motif between cpHsp70-2 and other cpHsp70s, cpHsp70-2 might play vital and unique role in the response of moss to land environment. The chloroplast- localized Hsp70 proteins from *P. patens*, *Arabidopsis* and rice have been reported to be essential for protein import into chloroplasts and for chloroplast differentiation under high temperatures^38^. Recently, Liu *et al.*^13^ demonstrated that a stromal Hsp70 in *P. patens* serves as a motor protein via ATP hydrolysis for the import of proteins into chloroplasts. In addition, stromal Hsp70s in *Arabidopsis* are important for thermotolerance of germinating seeds^39^, indicating that plastid physiology is important for seeds to endure heat stress. In rice, OsHsp70CP1 is essential for chloroplast development under heat-stress conditions^38^. Thus, land plants might share a general mechanism by which the stromal Hsp70s play roles in stress tolerance, possibly related to maintenance of chloroplast photosystem activity.

### Duplications Played Major Roles in the Diversification of *Hsp70* Gene Families

The *P. patens* genome is approximately 480 Mb organized as 27 chromosomes. Rensing *et al.*^40^ reported that *P. patens* genome duplication might have occurred between 30 and 60 million years ago, based on the construction of linearized phylogenetic trees. It was also predicted that tandem and segmental duplications contributed to expand the number and roles of gene families^41^.

In moss, *mtHsp70-2* and *mtHsp70-3*, *cHsp70-1* and *cHsp70-2*, *cpHsp70-3* and *cpHsp70-4*, *cpHsp70-4* and *cpHsp70-5* are all pairs of tandemly arrayed genes (Fig. 4b) that are closely related in the NJ tree (Fig. 3a, left), suggesting that they are the result of tandem duplication. Conversely, other pairs of *Hsp70* genes (*cHsp70-3*/*cHsp70-10*, *cHsp70-5*/*cHsp70-9* and *cHsp70-6*/*cHsp70-8*) are located at collinear positions on different chromosomes, and thus appear to have been copied during whole-genome duplication or other large-scale segmental duplication events. Their low Ka/Ks ratios indicate that these three gene pairs might have evolved under the influence of purifying selection, a phenomenon that has also been observed for *Hsp70* genes in *P. trichocarpa*^21^. Gene duplication often leads to expansion and functional diversity of this gene family^42^. Accordingly, our data support a model for the evolution of the moss *Hsp70* family involving a whole-genome duplication accompanied by multiple segmental and tandem duplications, suggesting that the moss *Hsp70* gene family might serve diverse functions in resistance to land-related stresses.

### *Hsp70* Genes could be Vital in Responses to Abiotic Stress

The Hsp70 ATPase is thought to be one of the most ancient proteins according to molecular clock analysis^43^. Hsp70 functions have been widely reported in various species, but mainly in heat shock responses and protein import, whereas research in drought response-related functions of Hsp70 is limited. The ER Hsp70, i.e., Bip of tobacco and soybean positively regulate drought resistance^44^. In addition, ER-resident *Hsp70-5* of *Citrus* has a key function in seed desiccation tolerance^44^. Here, we found evidence that *P. patens Hsp70* genes are expressed constitutively during development and differentially during stress treatment (dehydration and rehydration), suggesting that *Hsp70* genes have played critical roles in growth and in stress responses from the origin of land plants. Most *Hsp70* genes showed high expression in the gametophore stage, indicating their possible roles in the growth of *P. patens* (Fig. 5). The finding that *Hsp70* genes showed different expression during dehydration and rehydration stress demonstrates their sensitivity to stress and indicates their possible role in *P. patens* stress tolerance (Fig. 6). In *Chaetomorpha valida*, a bloom-forming green alga, *CvHsp70* most probably acts as stress-responsive gene that participates in protecting *C. valida* from environmental stresses^8^, suggesting that cytosolic Hsp70 might have evolved protective functions to help maintain rapid growth and allow successful colonization. In *Symbiodinium*, the cytosolic Hsp70 has been suggested as a potential stress biomarker^45^. These findings, together with our result that expression of *cpHsp70-2* was highest in *P. patens* during dehydration (R20%), and that *cpHsp70-2* was the most highly expressed after 8-h rehydration, illustrate that chloroplastic Hsp70 likely plays a prominent role in both growth processes and responses to drought stress. Furthermore, mRNA levels of *cpHsp70* continually increased after dehydration in *P. patens*, suggesting that the chloroplast might also be involved in preventing cellular dehydration and improving stress tolerance. It has been reported that *cis*-acting elements regulate the molecular processes of developmental and diverse stress responses^46^,^47^,^48^. Several elements including SORLIP2AT, DOFCOREZM, GCCCORE, and a novel one (GGCGGAGGGGGG) were overrepresented in the promoter regions of groups of *Hsp70* genes responsive to salt, ABA or drought (Supplementary Tables S5 and S6), which implies that *Hsp70s* are involved in responses to stress through shared evolutionarily conserved pathways.

From the data above, we hypothesized that moss *cpHsp70* played a critical role not only in protein import but also in adaption to dehydration stress. Considering its unique intron-exon structure, strongly active intron retention alternative splicing events, copy number, and known function under abiotic stresses, as shown in Additional Data 2^24^, we chose *PpcpHsp70-2* for further analysis. *PpcpHsp70-2* was previously found to be essential in moss, as the knockout was lethal^14^. Here, we found that moss plants overexpressing *PpcpHsp70-2* exhibited clear salt and dehydration tolerance (Fig. 8), which provides clear evidence for a role of *cpHsp70* in dehydration stress tolerance.

## Conclusion

In this study, we have identified 21 *Hsp70* genes from the genome sequence of *P. patens*. A comprehensive analysis of these genes, including of gene structure, phylogeny, gene duplication, expression profile, enriched *cis*-elements and dehydration tolerance, was performed. Our phylogenetic and evolutionary analysis based on *Hsp70* sequences points to a number of gene duplication events having taken place in this gene superfamily. Further, overexpression analysis showed that *PpcpHsp70-2* is involved in salt and dehydration tolerance. The information presented in this study provides detailed characterization of the *P. patens* Hsp70 protein superfamily and lays a foundation for further functional studies of these genes in *P. patens* development and dehydration stress.

## Materials and Methods

### Identification of Putative Hsp70 Proteins in *P. patens*

For Hsp70 retrieval in *P. patens* genome database v3.1 (http://phytozome.jgi.doe.gov/pz/portal. html#!info?alias=Org_Ppatens_er), *Arabidopsis* Hsp70 proteins containing conserved domain sequences were used as query to identify potential moss Hsp70 proteins, with a maximum E-value of 1e-5 (Supplementary Tables S1 and S2). Functional domains of PpHsp70 sequences were analyzed using SMART (http://smart.embl-heidelberg.de/) and PROSITE (http://prosite.expasy.org/). Predotar v.1.03 (https://urgi.versailles.inra.fr/predotar/predotar.html) and LocTree3 (https://rostlab.org/services/loctree3/) were used to predict the protein subcellular localizations^49^.

### Multiple Sequence Alignment and Domain Analysis

Multiple sequence alignment of all Hsp70 proteins was performed using MegAlign (v.7 Lasergene). To identify signature domains of Hsp70 proteins in *P. patens*, InterProScan 5 (http://www.ebi.ac.uk/Tools/pfa/iprscan5/) and SMART (http://smart.embl-heidelberg.de/) web programs were used^50^. ClustalX2.1 software and the ESPript tool were used to analyze the PpHsp70 sequences and structures^51^.

### Motif and Gene Structure Prediction

Conserved motifs were identified using MEME (http://meme.nbcr.net/meme/tools/meme), with parameters set as follows: numbers of repetitions = any, minimum motif width = 6, maximum motif width = 50, and maximum number of motifs to identify was 20; default values were used for other parameters^52^. Information on intron-exon structure was illustrated using Gene Structure Display Server (http://gsds.cbi.pku.edu.cn/). To identify the various *cis*-elements in the promoter of each cluster, 2 kb upstream sequences were extracted. Upstream sequences were downloaded from the Rice Annotation Project Database (http://rapdb.dna.affrc.go.jp/). The *Arabidopsis* Information Resource (http://www.arabidopsis.org/) and Joint Genome Institute Launches Phytozome v10.3 (http://phytozome.jgi.doe.gov/pz/portal.html#), respectively. *Cis*-element analysis was performed using MEME (http://meme.nbcr.net/meme/tools/meme), with parameters set as follows: numbers of repetitions = any, minimum motif width = 6, maximum motif width = 12, and maximum number of motifs to identify was 50; default values were used for other parameters^52^. The elements were annotated with PLACE (Supplementary Tables S3, S5 and S6, https://sogo.dna.affrc.go.jp/cgi-bin/sogo.cgi?lang=en&pj=640&action=page&page=newplace).

### Phylogenetic Analyses

The amino acid sequences encoded by the complete *Hsp70* gene families from other species, including *Escherichia coli*, *Saccharomyces cerevisiae*, *Chlamydomonas reinhardtii*, *Selaginella moellendorffii*, *Oryza sativa*, *Arabidopsis*, and *Populus trichocarpa*, were retrieved from Phytozome v10.3 (http://phytozome.jgi.doe.gov/pz/portal.html#) or NCBI (http://www.ncbi. nlm.nih.gov/) using BLAST searches. A phylogenetic tree was constructed in MEGA 6.06 (http://www.megasoftware.net/history.php) using the Neighbor-Joining (NJ) method. The bootstrap values reported for each branch reflect the percentage of 1,000 replicate trees containing that branch.

### Chromosomal Location and Gene Duplication Analysis

Chromosomal analysis was performed using Matlab programming language. The chromosomal positions of the *Hsp70* genes and the lengths of the chromosomes were obtained from Phytozome (http://phytozome.jgi.doe.gov/pz/portal.html#). Tandem and segmental duplications of *Hsp70* superfamily genes in *P. patens* were identified in PTG Base (http://ocri-genomics.org/PTGBase/) and Plant Genome Duplication Database (PGDD; http://chibba.agtec.uga.edu/duplication/), respectively.

### Analysis of Publicly Available Microarray Data

Microarray data (bulk accession numbers E-MTAB-914, E-MTAB-916, E-MTAB-917) from the public repository ARRAYEXPRESS (Hiss *et al.*^37^) (http://www.ebi.ac.uk/arrayexpress/) were used to analyze the expression profiles of *P. patens Hsp70* genes at different developmental stages (spore, protonema, juvenile, adult stage and gametophore) and under different treatments (dehydration, ABA and salt). The *P. patens* transcriptome data were obtained from Phytozome 10.3 (http://phytozome.jgi.doe.gov/pz/portal.html). In addition, the samples of rice and *Arabidopsis* used in the microarray data analysis included three abiotic stress conditions, i.e., salt, ABA and drought. The *Arabidopsis* microarray gene expression data were obtained from AtGenExpress. The public expression data in rice was obtained from the Michigan State University (MSU) Rice Genome Annotation (http://rice.plantbiology.msu.edu) databases (Supplementary Table S4).

### Plant Material, Stress Treatment and Chlorophyll Fluorescence Analysis

*Physcomitrella patens* (Gransden) wild type was maintained on BCD medium supplemented with 5 mM ammonium tartrate and 1 mM CaCl_2_ overlaid with cellophane, at 23 °C under continuous light (60 to 80 μmol photons m^−2^ s^−1^) for 2 weeks then transferred on the growth matrix block for another two weeks to get gametophytes. To examine the response to dehydration and rehydration stress in Fig. 6, we treated *P. patens* samples as follows. *P. patens* gametophytes were treated with air drying and water recovery, with 6 samples collected: dehydration samples (D), with relative water-content loss to 20%, 40%, 80%, and rehydration samples (R), with water recovery time to the 80% water-loss samples (4 h and 8 h).

The *cpHsp70-2* overexpression plants were a gift from Dr. Steven Theg in UC. Davis. In these plants, a *cpHsp70-2* knockout cassette (cloned into pCR4 TOPO vector) and rescue plasmid *(cpHsp70-2* cDNA cloned into pART7 vector) with 35S promoter and the OCS terminator were co-transformed moss protoplasts to generate rescued transgenic plants^14^. We refer to these rescued transgenic plants as *cpHsp70-2* overexpression transgenic plants because the mRNA and protein expression levels of these transgenic plants was much higher than those of wild-type moss^13^,^14^. The *cpHsp70-2* overexpression transformants and wild-type (WT) plants were grown for 4 weeks to obtain gametophytes. Then, 500 mM NaCl was used for salt treatment for 3 d, followed by recovery. For water loss measurement, leafy gametophores were weighed and placed on the laboratory bench (the relative humidity was between 30 and 40%) at 22 °C. Weight loss of the leafy gametophores was monitored for 1.5 h at the indicated time intervals. Water loss was expressed as the percentage of initial fresh weight. The leafy gametophores were then transferred to water for rehydration. Chlorophyll florescence of leaf gametophores was monitored using an IMAGING-PAM chlorophyll fluorometer and Imaging Win software (Walz, Effeltrich, Germany), as described previously, was measured under salt, dehydration and rehydration treatments. A dark-light induction curve was applied to assess dark- and light-adapted parameters. Plants were given a saturating pulse (>1,800 μmol photons· m^−2^·s^−1^) and the levels of *F*_*v*_*/F*_*m*_ were determined after 20 min of dark adaptation. *F*_*v*_*/F*_*m*_ was calculated as (*F*_*m*_ − *F*_*0*_)*/F*_*m*_. False-colour images of the *F*_*v*_*/F*_*m*_ parameter are presented through the Imaging Win software^53^.

### Quantitative Expression Analysis by Real-time PCR

Total RNA was isolated from dehydrated/rehydrated samples using TRIzol following the supplier’s instructions (Invitrogen, Argentina). RNA concentration was measured using a Nanodrop-2000 spectrophotometer (Thermo scientific). For each sample, 1 μg RNA was treated with DNaseI and reverse-transcribed using the PrimeScript™RT reagent Kit with gDNA Eraser (Takara, Japan). Reverse transcription quantitative real-time PCR (RT-qPCR) was carried out in a 25-μl reaction mix containing: 1 μl each primer (10 μM concentration), 1 μl cDNA sample and SYBP Premix Ex Taq II (Tli RNaseH Plus). RT-qPCR was performed using 96-well plates (Bio-Rad CFX96), with the program: 95 °C for 30 s, 39 cycles of 95 °C for 5 s and 60 °C for 30 s, followed by melting curve analysis (60 to 95 °C). The RT-qPCR assays were carried out with three biological replicates for each condition. The relative normalized expression was calculated using Bio-Rad CFX96 software with *Actin* expression (F: 5′CAGGGTGCGAGTGCGTATTG3′, R: 5′TCGGCAACGGAGACATAAGAGTA3′) for normalization.

## Additional Information

**How to cite this article**: Tang, T. *et al.* Sequence analysis of the *Hsp70* family in moss and evaluation of their functions in abiotic stress responses. *Sci. Rep.* **6**, 33650; doi: 10.1038/srep33650 (2016).

## Supplementary Material

Supplementary Information

## Figures and Tables

**Table 1:** *Hsp70* gene numbers in various species.

Organism	Cytoplasm	ER	Mitochondrion	Chloroplast	Truncated	Hsp110/SSE	Number of Genes
*E. coli*	0	0	1	0	2	0	3
*S. cerevisiae*	4	1	3	0	1	3	12
*C. reinhardtii*	1	2	1	1	1	0	6
*P. patens*	10	2	4	5	0	0	21
*S. moellendorffii*	4	2	1	2	1	2	12
*O. sativa*	11	6	3	2	0	0	24
*A. thaliana*	5	3	2	2	1	4	18
*P. trichocarpa*	10	4	2	2	2	0	20

**Table 2:** Divergence between paralogous *Hsp70* gene pairs in *P. patens*.

No.	Gene 1	Gene 2	Ka	Ks	Ka/Ks	Duplication
1	*PpcHsp70-5*	*PpcHsp70-9*	0.15	0.71	0.21	W
2	*PpcHsp70-6*	*PpcHsp70-8*	0.01	1.4	0.01	W
3	*PpcHsp70-3*	*PpcHsp70-10*	0.04	0.84	0.05	W
4	*PpcHsp70-1*	*PpcHsp70-2*	0.0008	0.0030	0.2774	T
5	*PpmtHsp70-2*	*PpmtHsp70-3*	1.94	4.43	0.44	T
6	*PpcpHsp70-3*	*PpcHsp70-4*	NA	NA	NA	T
7	*PpcpHsp70-4*	*PpcHsp70-5*	NA	NA	NA	T

Synonymous (Ks) and nonsynonymous substitution (Ka) rates were presented for each pair. Gene pairs were generated by tandem duplication (T) and whole-genome duplication or other large-scale segmental duplication (W).

## References

[b1] Feder M. E. & Hofmann G. E. Heat-shock proteins, molecular chaperones, and the stress response: Evolutionary and ecological physiology. Annu Rev Physiol 61, 243–282 (1999). 10099689 10.1146/annurev.physiol.61.1.243

[b2] Georgopoulos C. & Welch W. J. Role of the major heat-shock proteins as molecular chaperones. Annu Rev Cell Biol 9, 601–634 (1993). 8280473 10.1146/annurev.cb.09.110193.003125

[b3] Craig E. A., Gambill B. D. & Nelson R. J. Heat-shock proteins - molecular chaperones of protein biogenesis. Microbiol Rev 57, 402–414 (1993). 8336673 10.1128/mr.57.2.402-414.1993 PMC372916

[b4] Mayer M. P. & Bukau B. Hsp70 chaperones: Cellular functions and molecular mechanism. Cell Mol Life Sci 62, 670–684 (2005). 15770419 10.1007/s00018-004-4464-6 PMC2773841

[b5] Saidi Y. *et al.* The heat shock response in moss plants is regulated by specific calcium-permeable channels in the plasma membrane. Plant Cell 21, 2829–2843 (2009). 19773386 10.1105/tpc.108.065318 PMC2768932

[b6] Wang W. X., Vinocur B., Shoseyov O. & Altman A. Role of plant heat-shock proteins and molecular chaperones in the abiotic stress response. Trends Plant Sci 9, 244–252 (2004). 15130550 10.1016/j.tplants.2004.03.006

[b7] Brodsky J. L. & Chiosis G. Hsp70 molecular chaperones: Emerging roles in human disease and identification of small molecule modulators. Curr Top Med Chem 6, 1215–1225 (2006). 16842158 10.2174/156802606777811997

[b8] Deng Y. Y., Zhan Z. F., Tang X. R., Ding L. P. & Duan D. L. Molecular cloning and characterization of an Hsp70 gene from the bloom green alga *Chaetomorpha valida* (Cladophorales, Chlorophyta). J Appl Phycol 27, 489–497 (2015).

[b9] Karlin S. & Brocchieri L. Heat shock protein 70 family: Multiple sequence comparisons, function, and evolution. J Mol Evol 47, 565–577 (1998). 9797407 10.1007/pl00006413

[b10] Renner T. & Waters E. R. Comparative genomic analysis of the Hsp70s from five diverse photosynthetic eukaryotes. Cell Stress & Chaperones 12, 172–185 (2007). 17688196 10.1379/CSC-230R1.1 PMC1949330

[b11] Lin B. L. *et al.* Genomic analysis of the Hsp70 superfamily in *Arabidopsis thaliana*. Cell Stress Chaperon 6, 201–208 (2001). 10.1379/1466-1268(2001)006<0201:gaoths>2.0.co;2 PMC434401 11599561

[b12] Govind G. *et al.* Identification and functional validation of a unique set of drought induced genes preferentially expressed in response to gradual water stress in peanut. Mol Genet Genomics 281, 591–605 (2009). 19224247 10.1007/s00438-009-0432-z PMC2757612

[b13] Liu L., McNeilage R. T., Shi L.-x. & Theg S. M. ATP requirement for chloroplast protein import is set by the Km for ATP hydrolysis of stromal Hsp70 in *Physcomitrella patens*. The Plant Cell 26, 1246–1255 (2014). 24596240 10.1105/tpc.113.121822 PMC4001381

[b14] Shi L. X. & Theg S. M. A stromal heat shock protein 70 system functions in protein import into chloroplasts in the moss *Physcomitrella patens*. Plant Cell 22, 205–220 (2010). 20061551 10.1105/tpc.109.071464 PMC2828695

[b15] Hoekstra F. A., Golovina E. A. & Buitink J. Mechanisms of plant desiccation tolerance. Trends Plant Sci 6, 431–438 (2001). 11544133 10.1016/s1360-1385(01)02052-0

[b16] Brun F., Gonneau M., Laloue M. & Nogue F. Identification of *Physcomitrella patens* genes specific of bud and gametophore formation. Plant Sci 165, 1267–1274 (2003).

[b17] Schaefer D. G. & Zryd J. P. The moss *Physcomitrella patens*, now and then. Plant Physiol 127, 1430–1438 (2001). 11743086 PMC1540175

[b18] Floyd S. K. & Bowman J. L. The ancestral developmental tool kit of land plants. Int J Plant Sci 168, 1–35 (2007).

[b19] Rensing S. A. *et al.* The Physcomitrella genome reveals evolutionary insights into the conquest of land by plants. Science 319, 64–69 (2008). 18079367 10.1126/science.1150646

[b20] Hu W. H., Hu G. C. & Han B. Genome-wide survey and expression profiling of heat shock proteins and heat shock factors revealed overlapped and stress specific response under abiotic stresses in rice. Plant Sci 176, 583–590 (2009). 26493149 10.1016/j.plantsci.2009.01.016

[b21] Zhang J. *et al.* Hsf and Hsp gene families in Populus: genome-wide identification, organization and correlated expression during development and in stress responses. Bmc Genomics 16 (2015). 10.1186/s12864-015-1398-3 PMC4373061 25887520

[b22] Buckley P. T., Khaladkar M., Kim J. & Eberwine J. Cytoplasmic intron retention, function, splicing, and the sentinel RNA hypothesis. Wires Rna 5, 223–230 (2014). 24190870 10.1002/wrna.1203 PMC4449146

[b23] Chang C. Y., Lin W. D. & Tu S. L. Genome-wide analysis of heat-sensitive alternative splicing in *Physcomitrella patens*. Plant Physiol 165, 826–840 (2014). 24777346 10.1104/pp.113.230540 PMC4044832

[b24] Wu H. P. *et al.* Genome-wide analysis of light-regulated alternative splicing mediated by photoreceptors in *Physcomitrella patens*. Genome Biol 15 (2014). 10.1186/gb-2014-15-1-r10 PMC4054894 24398233

[b25] Cannon S. B., Mitra A., Baumgarten A., Young N. D. & May G. The roles of segmental and tandem gene duplication in the evolution of large gene families in *Arabidopsis thaliana*. BMC Plant Biol 4, 1–21 (2004). 15171794 10.1186/1471-2229-4-10 PMC446195

[b26] Cserhati M. Motif content comparison between monocot and dicot species. Genomics Data 3, 128–136 (2015). 26484161 10.1016/j.gdata.2014.12.006 PMC4535654

[b27] Hudson M. E. & Quail P. H. Identification of promoter motifs involved in the network of phytochrome A-regulated gene expression by combined analysis of genomic sequence and microarray data. Plant Physiol 133, 1605–1616 (2003). 14681527 10.1104/pp.103.030437 PMC300717

[b28] Corrales A. R. *et al.* Characterization of tomato Cycling Dof Factors reveals conserved and new functions in the control of flowering time and abiotic stress responses. J Exp Bot 65, 995–1012 (2014). 24399177 10.1093/jxb/ert451

[b29] Yanagisawa S. Dof1 and Dof2 transcription factors are associated with expression of multiple genes involved in carbon metabolism in maize. Plant J 21, 281–288 (2000). 10758479 10.1046/j.1365-313x.2000.00685.x

[b30] Brown R. L., Kazan K., McGrath K. C., Maclean D. J. & Manners J. M. A role for the GCC-box in jasmonate-mediated activation of the PDF1.2 gene of *Arabidopsis*. Plant Physiol 132, 1020–1032 (2003). 12805630 10.1104/pp.102.017814 PMC167040

[b31] Chakravarthy S. *et al.* The tomato transcription factor Pti4 regulates defense-related gene expression via GCC box and non-GCC box cis elements. Plant Cell 15, 3033–3050 (2003). 14630974 10.1105/tpc.017574 PMC282854

[b32] Sarkar N. K., Kundnani P. & Grover A. Functional analysis of Hsp70 superfamily proteins of rice (*Oryza sativa*). *Cell S*tress Chaperon 18, 427–437 (2013). 10.1007/s12192-012-0395-6 PMC3682022 23264228

[b33] Germot A., Philippe H. & LeGuyader H. Presence of a mitochondrial-type 70-kDa heat shock protein in Trichomonas vaginalis suggests a very early mitochondrial endosymbiosis in eukaryotes. P Natl Acad Sci USA 93, 14614–14617 (1996). 10.1073/pnas.93.25.14614 PMC26182 8962101

[b34] Liu S. H. *et al.* Characterization and expression analysis of a mitochondrial heat-shock protein 70 gene from the Antarctic moss *Pohlia nutans*. Polar Biol 37, 1145–1155 (2014).

[b35] Alvim F. C. *et al.* Enhanced accumulation of BiP in transgenic plants confers tolerance to water stress. Plant Physiol 126, 1042–1054 (2001). 11457955 10.1104/pp.126.3.1042 PMC116461

[b36] Ramsey A. J., Russell L. C. & Chinkers M. C-terminal sequences of hsp70 and hsp90 as non-specific anchors for tetratricopeptide repeat (TPR) proteins. Biochem J 423, 411–419 (2009). 19689428 10.1042/BJ20090543 PMC3709441

[b37] Hiss M. *et al.* Large-scale gene expression profiling data for the model moss *Physcomitrella patens* aid understanding of developmental progression, culture and stress conditions. Plant J 79, 530–539 (2014). 24889180 10.1111/tpj.12572

[b38] Kim S. R. & An G. Rice chloroplast-localized heat shock protein 70, OsHsp70CP1, is essential for chloroplast development under high-temperature conditions. J Plant Physiol 170, 854–863 (2013). 23394789 10.1016/j.jplph.2013.01.006

[b39] Su P. H. & Li H. M. *Arabidopsis* stromal 70-kD heat shock proteins are essential for plant development and important for thermotolerance of germinating seeds. Plant Physiol 146, 1231–1241 (2008). 18192441 10.1104/pp.107.114496 PMC2259073

[b40] Rensing S. A. *et al.* An ancient genome duplication contributed to the abundance of metabolic genes in the moss *Physcomitrella patens*. Bmc Evol Biol 7 (2007). 10.1186/1471-2148-7-130 PMC1952061 17683536

[b41] Zhang J. Z. Evolution by gene duplication: an update. Trends Ecol Evol 18, 292–298 (2003).

[b42] Lynch M. & Conery J. S. The evolutionary fate and consequences of duplicate genes. Science 290, 1151–1155 (2000). 11073452 10.1126/science.290.5494.1151

[b43] Caetano-Anolles G., Kim K. M. & Caetano-Anolles D. The phylogenomic roots of modern biochemistry: origins of Proteins, cofactors and protein biosynthesis. Journal Mol Evol 74, 1–34 (2012). 10.1007/s00239-011-9480-1 22210458

[b44] Yu A. M. *et al.* Roles of Hsp70s in stress responses of microorganisms,plants, and animals. Biomed Res Int (2015). 10.1155/2015/510319 PMC4663327 26649306

[b45] Rosic N. N., Pernice M., Dove S., Dunn S. & Hoegh-Guldberg O. Gene expression profiles of cytosolic heat shock proteins Hsp70 and Hsp90 from symbiotic dinoflagellates in response to thermal stress: possible implications for coral bleaching. Cell Stress Chaperon 16, 69–80 (2011). 10.1007/s12192-010-0222-x PMC3024090 20821176

[b46] Jiang C., Iu B. & Singh J. Requirement of a CCGAC cis-acting element for cold induction of the BN115 gene from winter *Brassica napus*. Plant Mol Biol 30, 679–684 (1996). 8605318 10.1007/BF00049344

[b47] Narusaka Y. *et al.* Interaction between two cis-acting elements, ABRE and DRE, in ABA-dependent expression of *Arabidopsis* rd29A gene in response to dehydration and high-salinity stresses. Plant J 34, 137–148 (2003). 12694590 10.1046/j.1365-313x.2003.01708.x

[b48] Singh A. *et al.* Comprehensive genomic analysis and expression profiling of phospholipase C gene family during abiotic stresses and development in rice. Plos One 8 (2013). 10.1371/journal.pone.0062494 PMC3640072 23638098

[b49] Small I., Peeters N., Legeai F. & Lurin C. Predotar: A tool for rapidly screening proteomes for N-terminal targeting sequences. Proteomics 4, 1581–1590 (2004). 15174128 10.1002/pmic.200300776

[b50] Zdobnov E. M. & Apweiler R. InterProScan–an integration platform for the signature-recognition methods in InterPro. Bioinformatics 17, 847–848 (2001). 11590104 10.1093/bioinformatics/17.9.847

[b51] Gouet P., Robert X. & Courcelle E. ESPript/ENDscript: extracting and rendering sequence and 3D information from atomic structures of proteins. Nucleic Acids Res 31, 3320–3323 (2003). 12824317 10.1093/nar/gkg556 PMC168963

[b52] Bailey T. L., Williams N., Misleh C. & Li W. W. MEME: discovering and analyzing DNA and protein sequence motifs. Nucleic Acids Res 34, W369–W373 (2006). 16845028 10.1093/nar/gkl198 PMC1538909

[b53] Woo N., Badger M. & Pogson B. A rapid, non-invasive procedure for quantitative assessment of drought survival using chlorophyll fluorescence. Plant Methods 4, 27 (2008). 19014425 10.1186/1746-4811-4-27 PMC2628343

