# Supplementary material for: Sequence analysis of the Hsp70 family in moss and evaluation of their functions in abiotic stress responses
Source: Sci Rep. 2016 Sep 20;6:33650. doi: 10.1038/srep33650 (PMC5028893; doi:10.1038/srep33650)
Supplement: Supplementary Information [file srep33650-s1.pdf]

Sequence analysis of the *Hsp70* family in moss and evaluation of their functions in abiotic stress responses

Ting Tang[1,2,3&], Anmin Yu[1,2,3,4&], Ping Li[1,2,3], Hong Yang[1,2.3], Gaojing Liu[1,2.3], and Li Liu[1,2.3]*

1. Key Laboratory for Plant Diversity and Biogeography of East Asia, Kunming Institute of Botany, Chinese Academy of Sciences, Kunming, 650201, China

2. Key Laboratory of Economic Plants and Biotechnology, Kunming Institute of Botany, Chinese Academy of Sciences, Kunming, Yunnan 650201, China

3. Yunnan Key Laboratory for Wild Plant Resources, Kunming, Yunnan 650201, China

4. University of Chinese Academy of Sciences, Beijing, 100049, China

[&] These authors contributed equally to this work.

* Corresponding author, email: liulia@mail.kib.ac.cn

Address: 132 Lanhei Road, Kunming 650201, Yunnan, China

Fax number: +86 087165216952

Table S1 The putative *Hsp70* genes in *P. patens*

	query id	subject id	% identity	alignment length	mismatches	gap opens	q. start	q. end	s. start	s. end	evalue	bit score
1	AT5G02500.1	Phpat.003G105300.1.p	91.3	653	54	2	1	652	1	651	0	1219
2	AT5G02500.1	Phpat.011G003900.1.p	90.4	653	58	3	1	652	1	649	0	1211
3	AT5G02500.1	Phpat.007G103800.1.p	90.2	652	62	1	1	652	1	650	0	1208
4	AT5G02500.1	Phpat.007G084200.1.p	90.2	652	61	2	1	652	1	649	0	1204
5	AT5G02500.1	Phpat.011G025600.1.p	89.7	652	64	1	1	652	1	649	0	1201
6	AT5G02500.1	Phpat.012G032000.1.p	85	653	97	1	1	652	1	653	0	1130
7	AT5G02500.1	Phpat.004G083500.1.p	84.5	652	99	1	1	652	1	650	0	1128
8	AT5G02500.1	Phpat.010G067200.1.p	65.7	613	204	4	7	618	31	638	0	831
9	AT5G02500.1	Phpat.003G101600.1.p	64.6	613	211	4	7	618	31	638	0	814
10	AT5G02500.1	Phpat.003G105200.1.p	92.6	352	26	0	1	352	1	352	0	690
11	AT5G02500.1	Phpat.003G105200.1.p	81.6	174	29	2	477	649	##	520	0	277
12	AT5G02500.1	Phpat.007G102300.1.p	56.1	508	215	5	8	510	9	513	0	591
13	AT5G02500.1	Phpat.011G026700.1.p	54.7	499	219	5	9	502	10	506	0	561
14	AT5G02500.1	Phpat.002G053100.1.p	50.4	595	268	11	2	590	47	620	0	561
15	AT5G02500.1	Phpat.001G091100.1.p	50.1	605	273	12	2	600	45	626	0	561
16	AT5G02500.1	Phpat.001G091000.1.p	50.1	605	273	12	2	600	45	626	0	561
17	AT5G02500.1	Phpat.002G052800.1.p	50.1	595	270	11	2	590	49	622	0	557
18	AT5G02500.1	Phpat.007G061800.1.p	49.7	578	270	9	10	582	82	643	0	538
19	AT5G02500.1	Phpat.007G061900.1.p	49.7	578	270	9	10	582	82	643	0	538
20	AT5G02500.1	Phpat.007G061700.1.p	49.7	578	270	9	10	582	82	643	0	538
21	AT5G02500.1	Phpat.026G035400.1.p	47	640	311	12	10	642	80	698	0	538
22	AT5G02500.1	Phpat.004G105700.2.p	47.5	646	292	15	10	643	78	688	0	532
23	AT5G02500.1	Phpat.004G105700.1.p	47.5	646	292	15	10	643	78	688	0	532
24	AT5G02500.1	Phpat.004G105700.3.p	47.5	646	292	15	10	643	78	688	0	532
25	AT5G02500.1	Phpat.003G001400.1.p	53.5	508	215	6	10	507	11	507	0	516
26	AT5G02500.1	Phpat.020G074800.1.p	35.6	410	260	3	10	418	4	410	0	280
27	AT5G02500.1	Phpat.010G079900.1.p	31.3	543	339	9	10	535	4	529	0	278
28	AT5G02500.1	Phpat.024G005800.1.p	32.9	410	253	4	10	418	4	392	0	247
29	AT5G02500.1	Phpat.014G073900.1.p	27.3	521	342	8	7	494	27	543	0	226

Table S2 *Hsp70* gene families in *P. patens*

Gene Name	Locus	Genomic position	Length (aa)	pI	MW (kDa)	Subcellular localization predictions
PpmtHsp70-2	Phpat.001G091000	Chr01:17,126,371..17,130,194	676	6.19	72	mitochondrion
PpmtHsp70-3	Phpat.001G091100	Chr01:17,133,788..17,136,541	676	6.19	72	mitochondrion
PpmtHsp70-1	Phpat.002G052800	Chr02:8,149,392..8,153,094	680	5.94	73	mitochondrion
PpmtHsp70-4	Phpat.002G053100	Chr02:8,196,411..8,199,708	678	5.82	72	mitochondrion
PpHsp70-BiP1	Phpat.003G101600	Chr03:17,656,022..17,658,623	662	5.07	73	ER
PpcHsp70-1	Phpat.003G105200	Chr03:18,198,892..18,201,354	520	5.16	57	cytoplasm
PpcHsp70-2	Phpat.003G105300	Chr03:18,246,365..18,248,697	650	5.12	71	cytoplasm
PpcHsp70-3	Phpat.004G083500	Chr04:14,397,874..14,400,506	660	5.07	72	cytoplasm
PpcpHsp70-2	Phpat.004G105700	Chr04:19,181,152..19,189,094	707	5.4	76	chloroplast
PpcpHsp70-3	Phpat.007G061700	Chr07:10,405,881..10,409,515	714	5.25	76	chloroplast
PpcpHsp70-4	Phpat.007G061800	Chr07:10,424,865..10,428,592	714	5.25	76	chloroplast
PpcpHsp70-5	Phpat.007G061900	Chr07:10,453,098..10,456,825	714	5.25	76	chloroplast
PpcHsp70-4	Phpat.007G084200	Chr07:15,180,727..15,183,782	648	5.13	71	cytoplasm
PpcHsp70-5	Phpat.007G102300	Chr07:17,602,178..17,604,100	518	5.48	58	cytoplasm
PpcHsp70-6	Phpat.007G103800	Chr07:17,790,078..17,793,122	649	5.13	71	cytoplasm
PpHsp70-BiP2	Phpat.010G067200	Chr10:11,770,163..11,772,910	662	5.12	73	ER
PpcHsp70-7	Phpat.011G003900	Chr11:467,447..470,376	648	5.19	71	cytoplasm
PpcHsp70-8	Phpat.011G025600	Chr11:4,177,991..4,181,113	648	5.09	71	cytoplasm
PpcHsp70-9	Phpat.011G026700	Chr11:4,434,823..4,436,556	509	5.28	57	cytoplasm
PpcHsp70-10	Phpat.012G032000	Chr12:17,115,540..17,435,539	652	5.42	71	cytoplasm
PpcpHsp70-1	Phpat.026G035400	Chr26:6,686,993..6,690,627	710	5.25	76	chloroplast

Table S3 Multilevel consensus sequences of MEME-derived motifs

Motif	Width(aa)	E-value	Best possible match
1	50	1.5e-685	VKNAVVTVPAYFNDSQRQATKDAGRIAGLNVLRIINEPTCASIAYGMDRK
2	41	1.4e-468	HFYSTITRACFEELCMHMFRRCMEPCEKCLRDAKMDYKDIH
3	41	1.5e-515	NNLLGKFELSGIPPAPRGVPQIEVCFDIDANGILNVSAMDK
4	50	2.6e-632	VQDILLLDVTPLSLCLETMGGVMTRLIPRNTTIPTKKSQVFSTYHDNQPS
5	50	1.4e-660	FDLGGGTFDVSLLTIEEGVFEVKATNGDTHLGGEDFDNRMVHHFVQEFKR
6	29	8.50E-278	ERLIGDAAKNQVAMNPTNTFFDVKRLIGR
7	41	8.1e-390	KYGKDISSHPRALQRLREAAERAKRELSSTHQTTIEIPFLY
8	44	4.2e-388	KDEIEKMVQEAEKYKQEDEEHKKKIDAKNHLENYMYNMRNQIKE
9	21	4.00E-190	GIDLGTTYSCVGVWQHDHHEI
10	29	5.0e-316	CEFEEKMFEPEEICNMVLIKMYQIAEAYL
11	29	6.90E-287	FNGKEPCKSINPDECVAYGAAVQAGILSG
12	15	5.50E-159	EVVLVGGSTRIPKVQ
13	15	4.90E-141	NDQGNRTTPSWVAFT
14	21	2.50E-134	RFSDPSVQSDMKMWPYKITPG
15	15	5.90E-105	VCIQVYQGEREMTRD
16	15	2.00E-100	TGKKQKITITNDKGR
17	21	2.30E-95	DKQKMEDAMEAAIQWLMQNQQ
18	29	5.00E-68	PEAQMMNQQGGSSGPGAGGDQVIDAEYTD
19	21	2.40E-46	MASPCPWRHGSSLLHQRTGRA
20	40	5.70E-32	FSHRRNAFFGGDVKSRAGFGVEKLRAKSGGQCRRAHGPMR

Table S4 Analysis of publicly available microarray data

Gene name	Spore	Protonema	juv-Gam	ad-Gam	Gametophore	GeneName	Control	Drought 3h	salt 3h	ABA 1h	ABA 3h	ABA 6h	GeneName	Control	ABA 0.5 h	ABA 4.0 h	Dehydration 0.5 h	Dehydration 4.0 h	Salt 0.5 h	Salt 4.0 h
PpcHsp70-10	8987.30	8115.46	6192.69	8741.27	8877.29	OscHsp70-1	1.00	2.44	2.24	0.97	1.83	1.99	PpmtHsp70-2	1.78	1.60	1.74	1.68	1.48	1.59	2.14
PpcHsp70-2	41740.63	47802.56	220326.26	513464.29	173743.17	OscHsp70-3	1.00	0.39	0.66	1.33	1.02	2.03	PpmtHsp70-3	1.16	0.94	1.15	0.75	0.85	0.70	1.34
PpcHsp70-3	8364.33	6826.23	11937.98	5118.35	7853.37	OscHsp70-4	1.00	5.81	6.31	1.30	1.99	2.05	PpmtHsp70-1	1.89	1.99	1.86	1.86	2.02	1.73	2.03
PpcHsp70-4	9720.95	10168.49	9343.45	15995.28	10516.18	OscHsp70-6	1.00	6.33	5.25	1.12	1.70	1.38	PpmtHsp70-4	1.92	2.05	1.94	1.89	1.75	1.80	1.90
PpcHsp70-6	40814.64	52893.46	102830.78	366837.27	45368.65	OscHsp70-10	1.00	1.13	0.92	0.95	1.02	1.58	PpcHsp70-2	1.92	2.68	1.88	2.63	2.53	2.53	2.26
PpcHsp70-7	15605.72	11064.17	19031.63	35458.46	12422.05	OsuHsp70-2	1.00	0.55	0.72	0.26	0.52	1.32	PpcpHsp70-2	0.79	1.47	0.92	1.41	1.86	1.52	1.60
PpcHsp70-8	29869.08	14692.26	12210.92	44984.12	12231.88	OsHsp70-BiP2	1.00	4.41	3.03	1.17	1.25	1.08	PpcpHsp70-5	2.32	1.75	2.30	1.65	1.89	1.81	2.12
PpcpHsp70-1	11370.54	9461.38	14308.66	22323.82	9269.16	OsHsp70-BiP4	1.00	0.70	0.36	1.04	1.14	2.34	PpcHsp70-4	2.74	2.36	2.74	2.11	2.47	2.04	2.44
PpcpHsp70-2	25116.21	52482.66	55513.81	47819.97	27103.15	OsmtHsp70-2	1.00	0.94	1.45	1.22	1.90	2.04	PpcHsp70-6	2.69	2.43	2.69	2.28	2.76	2.38	2.56
PpcpHsp70-3	80475.52	66713.18	74968.55	209053.21	118244.21	OscpHsp70-1	1.00	0.68	0.68	1.12	1.81	1.80	PpHsp70-BiP2	2.27	1.79	2.22	1.50	2.21	1.94	2.09
PpHsp70-BiP1	6911.30	8477.26	4329.52	5836.64	7456.21	OsHsp110-1	1.00	1.66	1.33	1.18	1.80	1.78	PpcHsp70-7	0.70	0.09	0.64	-0.25	0.83	-0.20	0.29
PpHsp70-BiP2	189599.61	353522.73	254492.57	330581.42	60652.69	OsHsp110-2	1.00	0.80	0.75	1.88	2.20	1.40	PpcHsp70-8	1.88	1.95	1.84	1.91	1.81	1.36	1.69
PpmtHsp70-1	94321.29	58401.52	64240.77	58249.13	25193.28	OsHsp110-3	1.00	0.99	1.20	1.32	1.70	1.83	PpcpHsp70-1	1.44	1.15	1.45	0.88	1.38	1.01	1.79
PpmtHsp70-2	10785.35	10240.76	11252.01	21758.31	9792.31	OsHsp110-4	1.00	0.64	0.69	1.61	1.96	1.88								
PpmtHsp70-3	10785.35	10240.76	11252.01	21758.31	9792.31	OsHsp110-5	1.00	7.86	3.36	0.98	1.05	1.58								
PpmtHsp70-4	14439.15	9490.34	11632.99	21308.01	9838.79	OsHsp110-8	1.00	1.67	2.83	0.53	1.37	1.90								

Gene-name	Control	Salt 0.5h	Salt 1h	Salt 3h	Salt 6h	Salt 12h	Salt 24h	Drought 0.25h	Drought 0.5h	Drought 1h	Drought 3h	Drought 6h	Drought 12h	Drought 24h	ABA 0.5h	ABA 1h	ABA 3h
AtcHsp70-1	1.00	0.72	0.70	0.64	0.79	1.31	1.37	1.02	0.93	0.89	0.78	0.61	1.05	0.95	1.05	1.00	0.80
AtcHsp70-3	1.00	0.32	0.26	0.35	0.51	1.00	1.55	0.80	0.66	0.62	0.40	0.21	0.84	0.66	0.85	0.81	0.44
AtcHsp70-4	1.00	0.27	0.10	0.17	0.35	0.74	4.40	0.36	0.25	0.29	0.32	0.12	0.21	0.28	0.05	0.21	1.86
AtcHsp70-5	1.00	0.85	1.05	0.77	0.99	1.08	1.64	0.84	1.01	0.88	1.70	2.09	1.19	1.12	0.58	0.56	0.57
AtcHsp70-6	1.00	0.88	0.82	0.52	0.41	0.60	0.79	0.98	0.93	1.01	0.83	0.57	0.82	0.77	0.62	0.56	0.44
AtcHsp70-7	1.00	0.54	0.47	0.17	0.15	0.44	0.58	0.87	0.63	0.62	0.26	0.23	0.67	0.52	0.37	0.30	0.14
AtcHsp70-8	1.00	0.76	0.77	0.71	0.68	0.76	1.89	0.65	0.68	0.67	0.66	0.80	0.60	0.81	0.24	0.21	0.19
AtcHsp70-15	1.00	0.69	0.62	0.48	0.73	1.02	1.59	0.96	0.98	0.93	0.86	0.60	0.87	0.75	1.32	1.23	0.74
AtcHsp70-16	1.00	1.09	1.16	0.96	1.18	1.06	1.16	0.82	0.95	0.77	1.09	1.39	1.21	0.77	1.63	1.72	1.54
AtcHsp70-17	1.00	0.71	0.86	0.73	1.07	1.24	1.23	0.88	0.93	0.93	1.29	0.95	1.23	0.83	1.51	1.28	0.80
AtcHsp70-18	1.00	1.10	1.20	1.08	1.06	1.07	1.23	1.10	1.00	1.01	0.94	1.04	0.97	1.18	1.07	1.07	1.05
AtcpHsp70-1	1.00	0.88	0.82	0.52	0.41	0.60	0.79	0.98	0.93	1.01	0.83	0.57	0.82	0.77	0.62	0.56	0.44
AtcpHsp70-2	1.00	0.54	0.47	0.17	0.15	0.44	0.58	0.87	0.63	0.62	0.26	0.23	0.67	0.52	0.37	0.30	0.14
AtHsp70-BiP1	1.00	0.59	0.59	0.35	0.81	1.10	0.99	0.67	0.65	0.64	1.12	0.67	1.34	0.59	1.37	1.34	0.78
AtHsp70-BiP3	1.00	0.67	0.65	0.64	0.68	0.55	1.35	0.68	0.55	0.55	0.85	0.62	0.78	0.63	0.59	0.69	0.50
AtmtHsp70-1	1.00	0.76	0.75	0.29	0.40	0.67	0.79	1.04	1.01	0.91	0.67	0.39	0.59	0.72	0.93	0.89	0.59
AtmtHsp70-2	1.00	0.54	0.44	0.50	0.55	1.01	2.33	0.71	0.75	0.77	0.79	0.47	0.63	0.68	1.25	1.36	1.07

Table S5 Promoter sequences of *Hsp70* gene family under treatment of ABA, salt and drought.

		ABA
AtcHsp70-15	AT1G79920.1	TATGCGGAACAAAGTGAGAATCTTTATTTCGTCTTTTCATTGGCAGTATGCATATGTAAAGTTGTGAGTATTGGGTACTAACGTGTCTTGTTTTCAGCTGAGTGACAAATACCAGGAATATATCACTGACTCGGAGAGGGAAGCAT<br>TCCTGGCGAATCTGCAGGAAGTTGAGGATTGGTTGTATGAAGATGGGGAAGATGAGACCAAAGGTGTTTATGTTGCGAAGCTCGAGGAGCTCAAGAAGGTAATGCATGCATATGATGACACTTTATATTGTGCAGGAAGAAATC<br>ATATTTCAGGATAATGTTTTAACAGAGGAGTGGGATTAATATTTTGCAGGTGGGTGACCCTGTTGAAGTGCGTTACAAGGAGTCATTGGAAAGAGGGTCGGTCATTGATCAGCTTGGTTACTGTATTAACAGCTACAGAGAGGCA<br>GCTATGTCTACTGATCCCAAGTTTGATCACATTGAATTGGCAGAGAAGCAAAAGGTAATAATTTGGCTCAATCTTGGAAGCACAAAAAAAATCTTAAATTTCTCAGGCTTGGGGAAATTGTTTGATGTTTTGTTGTTGTGAAATGA<br>TTAAGGTTTTGAACGAGTGTGTGGAAGCAGAAGCATGGCTGCGGGGAAAGCAGCAGCAGCAGGACACACTTCCCAAGTATGCCACACCGGCTCTCTTGTCAGCTGACGTTAAAAGCAAGGCAGAGGCATTGGACAAGTAAGCA<br>AGGCTCCTTGTGTCTTTGTCTTTTAAAAGAAATGCTAAATCTGATAATAGAGAGTGAACCAGCTATGGATGTTTTACAGGTTTTGCAGGCCTATAATGACCAAACCAAAGCCGGTGGCTAAAGCAGAGGCACCACAAGCCAAGGG<br>AGGTGAGCAGGCGGATGAGGGCAAGAGTGAGCCAGAGCAACCAGCTTCTGCCGAACCAATGGAGACTGAGAATCCCGCGGAAGGTAGTACCTAAATGAATGACGAGAGAGAGATGGTGTTGTATCGATTTTGTTCTTAACTCTT<br>TTTGATTTACCTAGTGGAACCTCTTTTTCTGGTTTCGCTATTATTCCCGGAAAAAATCTATATTGTTAATTCTTCCTTTTCGAAAACAATTTTTAGGAAGCATCTTTTTTCTTATTGTTCCTTGTGGTTTTGTTTTAATTTGAAAGTCA<br>TTTTTTCTGCTCCTCTATATAACTATTATGGAGTGTACACGGATTCGTTCTATGTTGGACTGGGCTGTTTTTATAAACAGCAAATGAAATGCAATTTGGTTCTATATATATTGGAGCTTTAAAAGGCAACTTTGAACCCCCTAAGCTT<br>AACATCTTTTCTCTTATATCTTACGTGAAATCAGTGTGACAAAACTCGTCTTCCTCACTTCTCTTCCTTACCATTGTTGTACAAAGAACATCTTCCTTGATGAAGCAGTCGTAGCCTTTGGCAACATCTTTGAGGTCATCCAGGCTGA<br>AGTTGTATCGTTTCTCCAATCCAAACGGGGTTAAACAAGGATGAATTCATTTCCTCGTTATGAGCACAGAATGAGTGCGACTGCTACGATATGTGATTAAGCTGCCTGCACCAGTGAGGACAATGAAAATCTCTAACATAAATCC<br>ATGAATCATCTCTGGGCGCCGCAGATAATGGCATGTTCCAGACACCACAATGCAATTAGTTCGTGCATATCTTATGTACCACAAATTCTGATAACACCAGCCACTTCTCTATAATAATTTTATTAATTTTAAATACATTGGGTTTAA<br>ATAATTATATGTAGACTCGAATAAAAATATACAAAAACACTGGGATAAAAAAAAAAAACCTAAGAAAAAAAAAGGCAATAAATAAATAATTTAGGCAAAAATATCACAAAAATAAAAAGGAAATGAAAGTGTTTTTAAAATAA<br>AAAAAAAAAAGGAAAGGAGAAGAACATTCCAGAGGAACCCAAACCCTGTGGTGGTGTCTGCTCCTTAATTAAAACCTCTTCTCTCTTGCTCTTCCTTTATAAAAGA
AtcHsp70-16	AT1G16660.1	TTCTCAAGTTCATAACGTTATAGACTTATAGCTTTTCATAACCGAGGAAATTAGAATTCTTTCATTACAATAAAAAGAAAAGTCAGCGATTTTTCCCCTGCTAGATCAGTAATATTCCTAAATTATGTCCATTAATTGAAATCATT<br>ATTCATTAATTATAAGAAATCTATCAAAAAATTAAGAACTCAACAAAACTATTGATTAATGGTTTTTCCGACATGGACACAAAGGTTAAAACCAAATTCCACATATGTATTTTTGGGCTGCAAACTCGTAAATTAAAATGCTAAAG<br>TAGTAAATGTTATCTTATACATGTAAGTACTATATATATATATGACCTCATTTTTGCAAACAAATAAATTAAGATATTTGGACGGCAAGCTCTTAAATTTAAATGTTTACAGTCTAAGATGTTATCTTACAAATATATAAACTATATGA<br>CCTCATTTTGCATAATTAAATATGAACAATGATTTTTATATACAGGCATTAACTATAAGATTGTTAGCTGGAAAATTCATACTATCTTATATATTATCTTAATGATTTGACTTATAATTAAGTTTAATTGTCCTCTCTTTTTTTTTGGC<br>CAAAAAGTTTAGTTTCTAATAAGAAATTACATAACTTGAGAAAAAGAAAATAAGAAACACGTATACAAGCTACAAGTTAGTGCACCAGCACTGCTCAATGCAATGACTACAAATACTTGGTCATTTCAACTCCTCTCCTCATCTC<br>TCTCTGATCACACTACCTTCTCTTCCAAAAGTCGTCGCCGGGAAAACACAAAAAGACGAAAATGACCCTATTCAAGAGGCCATGTCTTTACTCAAAGATGGACAAGGAAGATCCAGAAGAGGTACTTAGCCGACGAGCCAAGTT<br>CTTGATTTACAAAACACTTCAAGAAGCCGACTTGATTTCTCGGCGTGATCCTCATTCGTCGTTTATACGGCTGAAGCTTTATCTGTTGAAGGTCAAGATCGGAAAGAGGTTGGCTAAGCTACGCCGGAGTGTAGTATCCGCCGTTA<br>GATTTGGCGGAATTCGAAAGCATTCTCATAATGGCGTGAGAGCGTTGAAGAAGATGTTCCAAGGTGGTGCAACAACTGGACTACCTAGGCCTATTTTCTCCCTTGAAGTTTGAAATTTCATTTATTTATATTTCTTGTTTTGTGTAA<br>TTTTTTTTCTTTTGTTTCCGGAGTTTCTTTTGATCTCTATTACAAAAGTTTATCAAGAAATGTAATATGAAGAGATCATGAGTTTTGTTTCTTTCAAAAAGGTAAAAAAAAAATCTTGAAATAATCATTTAACTATATGATGATCTAT<br>TTTTATTTTGTCAAATTTTAATTATATGATGATGCTAAAATATATATAAGACACACAGATTAGTGTAAATGTGAGTTATGTTTACTTAATTTTTGTACCTAAACTTTTTTTTTTTTTTTTTTTTTGCTAAAGAACCTAAACTTTTCAAAC<br>GCTCATCAAAGTGGTTAGAGATCAAATTTAAGTTTGATGTAAATGTGAGTTACGTTTACGTAATTTTTGTACCTAAACTTTTTTTTTTTGCTTATGAACCTAAACTTATTATTCAAACGCTCATCAAAATGGTTAGAGATCAAATTTG<br>AGTTTGGATATTTTGGATAGTCATAAAAAGCATGGTTGGATAGCTTTTTTCAAATTTCAAATTAGTACCACATATATGGATACAAATCAAGTAATTCATGCATGTTAGCAATATGTAAGTTCAAACGACCTACCTTTTAAAGCGAA<br>ATAAAATCTCAAGTCAAAAAGTTATTGAATCCGAAAATCCGATCCTACTCGATTGAAAGTTTGAAACATGTGGGGGCATTCGTGCGACATCGTGAATCAATCTTGCGCTAGGTTGTCCAAAAATATCTCTGGGATATAATATCAAT<br>TAAAAGGAATTAATAAGGACTTAAATGCAAATACATTTTTCTTTTCGTTAGCCTTTCCCTTGTCTCTTATTTTTCTAATTTCTAGCCCTTTC
AtcHsp70-4	AT3G12580.1	TGATTAGTGTGACTGAACCTAACCGGTGCGTTTGTAATATAAATTCTTGTCATTATTTTGGCTGCCACTATCTGATCTGAGAAGCCACAAGGAGTTGAAGTTTACAGTTGTTAAAATCAATTTGACAAACAAAAAATCAAGAGAGA<br>AACATACAATTACCAATTTGATCCACAGATAAATCAATTGTTAAAAAGTAATTAAATACTACTATTGGGCCAGGCCTGCAAAATAAAGACCCTCAGACATCGAAACCCTGAGAGTGAGATCCAATACTTGTTCTGTTCTGCAAAT<br>CGCTTCTGGTCCCTGGAGTCTGGAGATCTCATCCTTTCCCACCGATTCCGTCCCGATAAAACGGTAAATCTCGCTCAATTTTAAAACTATGTATATATATGCATTTCTTCTTGAGTGATCTTCTGATTCGACGACAAGTGTGTTTGAT<br>TTATGATCTGGTTAGTCTTAGACTCTTAGCTGTTCTTCTCGCGATAGAGATTGTCTCTGTGGCGGCCATCTGTGTGTGATGGTCCTTTGAATCCGTCTCTGTCGACGAGAACTGCCAAATTCTCTTGTCGGATTGTGTCACGTTTTAG<br>GTTTTTACATAAATTCTCAACTTGTTCTCCAATGTGATCCCATAGCGGAGTGGATATCGCGTTAGACTCCGAATCTAAAGGTCGTGGGTTCGATTCCCACTGGGATCATCCCATTTTTTTGGTCTTTTTTTTTGTTGGTTATTAATTAT<br>TACTATCAATCCGTTCTTTTAGCAGTGATCAAGCGAATTTAGGTCTGGCTCTGCCTCTGGCTCTAGTTGAGTTTAGGGAGCTGCTCCTTGTTGTTTGCCATTATGGTTTCTCTACTTTGGAGTCAACTTTGCAATGTTACCCTTGTAGT<br>CTCTTCTGTGGACTTTGTATTTGGTGAAATTGAAAAGTTTAGTAGAAATCTGGCTGCGCCAAAGATGATAGAGACTCTAATGGGATCCTTATTCGAACATTTTGCTGAACTGATTAGATACAATAAGGCAACACTGATTCCATTAC<br>TGTTAGTCTGTTACTACAAAAAGAATTGTTGCTCAAGTATTGTTTGTGTTGGTTGGTGCAGATGATCGATGATCAGGACTTGGGGTTTATTGCCAACTTTCTTGGCATCTTCATCTTCGCATTGGTAATTGCTTATCACTACGTAACT<br>GCTGATCCCAAATACGAAGCCACTTGAGTGATGATATTTTAGAATGATGTAAGGCTTTTTAGTTTATACTAGTATTATCTGTGTTTCAAACTGAGAAGAGATAATAACAGTCTTTGTTGAGATGATAATGTTTTCAAGATGTTCCTA<br>ATCCATTTCACATCTTCTCAATTTTATATGCATGTGCATATATATGTTCCCTCCAATTATGTTGTTCGAATGTTTGATGAAACTTTGAATTTTTTTCTTTAAGCAAAAAAAAATCTCAAACACCAAAGCGAGGAGTCATTCTAGTTCA<br>GTTTTGAGTCATTCTAGTTATTTTTACAAAGTTTTGAGTCAAATTGGGTAAATTTTTTGGTTATTTTGGTCATAAAAATAACTAGATTATCTCTTATATCTTATGAGTTAATTTGGTAAATAAACCATTTATTTGGGTCAAACTATTTT<br>TTTCCCCATATATATATATCCAATCAATAATAAATTCATAATATATTTCATTAACGCGATTGAAATACTAGTAATTAATTGAGGACTAAAGAAAAAGTAATTTCCTTTTTATCTTTAAAATGTGCAAAAAAAACAAAAATGTTAATTG<br>GGTGATGAAATAACTTGTTTTCAAAACGGGAGTTACTATTTGACAATTTAAAAAAGACCCATCTCGAAGGAGCTAGAAGCGATAACAAAATAAAAAGGAAACAATAGTAATTAGATGGCGCAAAAATAAGATCCAACGGCTGA<br>GATCTTTACTCGTGAACGTTCTCGAAAGCTCTTTGCCGACCCACTCTTCATTCATATATAAACAAACACCTCTCTGCCTTCTCTTCCT
AtHsp70-BiP1	AT5G28540.1	TCCACACCAAGACGGAATAGAAATACTGAAGATATTGACTCTCCAATTCGTAGAATGGTATATATCTTAAATGATTTGAGTTTATTTTAAATTGTTATTTGTATTCTAATTTTTTTAATTATGATGTTTCATTTAATTATTCAAGTAT<br>CATTTAATTCACATAACTTTAGTTTAATATTTTAAAAATATTTAGATTAAATTATTTTTTGAATTTATTTACCATTTTAGTGTTATTTAATTGATTGTAATTAATCATCGTAGAGAAAACATGATCAAAGATATATACTTGAAGAAGA<br>AAGAACGTTGATTGAATTATATGATGAAACACTAGTTTTAAACAATTACATGTTTAAAGATCCGGGACAACCGGGACGAGCATATATTGTTCAAAAAATTCAATGAAAACATCAAGTGAACAGATTTAAGATGCAAATTCGATGA<br>GTGGAAGAAACAATACAAAAGATGGATCCTTTTACATGGTAAGACTGGTATTAGGGTAGATACCATTAGTGGAAGAATTGAAGCTTCAAATGAGTGGTGTAATGAACGTATACATGTAAATTAATCTTCTTAAGTATTAATATGG<br>TTTAACTATTTGTAATTTTTTTTATTATGCGGTTACAATTTTTAATATTATATTTTTGCAAGAAAACGACAGAGTGGGACGTTTTCAACGAGAACCTTCTAAGTTTTGGGAAATCTGAAACGTCCCATCCCGAATCGAGGATGTTAC<br>AAAATCATACGTATATGTTTTGCAAATCTTGATGTGGGTTCTCAACGTCAACATTCAGTGCGACAAAGAAGAGAAGAACTGATAAGAGAGAATGAAAGTTGTTTAGAGGATAGAAAGATGCATATGATGAAGAAAATGCATATA<br>GAAAACGAGCAAAATGACTCAAATGAACAAGAGGAGGTTGAGAGAGAAGATAGACAATATATGGCAAATATCTAAGAAAGTAGTGTCAATATGTTATTTTGATTTATAATTTTTAAAATATTGTTGTAATAGTTATTAAAATGAT<br>ATGTTATTAATTATTAAAATTGTTAAAATTTGTTTTGGTTTTCGGTACACACTTTTGCATACTACAGTACGTTTTTAACCATTCAAAGGATAAATTAAAGTTAGTACGCATGACTCCTTACCAAGATGTTAGTACGCATGTCTTAGG<br>ATAGAACATACCATTCAATCATTAAAGGCTTTAATGGGTCATTTCACTTGGGCTTCTAAAATTTAATATCGGTTCTATTTATTTTAGATTAGAACTAATTAAGTTAATCTCATTCTTCATAATATAATCTCATTTATGAAAGAATATT<br>TTTTTTAAATGATAACAAAATAAATATAAAACAAATACTTTTTAAAAAATCAATAGCAAAAATAGATAGTACGTTTCTCTTTGTTAACAATGATGCTTAGGGGTAGTAGACATTCGGGTACGCAGGTCGGGTTTGGATAAAATCC<br>GATCGGGTAAGGATCCTTTGGGTTGAGAAGTTTAGGATTCAATAGGATAAATCTAAAATCTAAATTTGTTTCCGGTTTGGGTCCAATCGGATTTGAGATTTTTTTACTATATTTTTCAGATCTTTTTTTGGAACTAATACCCAAACT<br>ATTGAGTAAATATCTGGATTTTTGGGTAAAATTCGGAATACGGATTCGGGTTCGGATTCGATTTTTTTGGATAGAAAAAATTTAGGATCCAATAAAATAATTTCTCAGCTTTTAAATTTAGATTTGTATCCAATTTTTCGGATGGTTT<br>GGATTATTCGGGTTAGGTTTTTCTTCGCAGCCCTAGTGATACTAATAGAAGAAGACTGGGCCCAACAAAAGCTCATTGTCTAGAACTAAGAAGAAGAACCGAAGTAACCAACGGCCACGATTACTCCAACACAAGACCAAATCT<br>GATTGGTTGACATTACAAATCGTCGTAAGATAATTGGTCCACGTCATCTCTGATGACATAGTTAAATTTCTTCGTCTTCCATAAAAAGCTTCTTCTTCAA
AtmtHsp70-2	AT5G09590.1	TGAGGATATCGATCTTCGGCGATGGATGTTACCAGATATCAAAGCTTTCAGCTTTGGTCTTAAGGATTGGCGCTCAAGGATCAATTGTATCATCAATCCTTACATGTACGAACAGGTCCGATGTCTATTCACATTGTTTCCTTTTGT<br>GAAATAATTAAACTCTTATTGAATCTGTGCATGTCCTTAAACCATTCCTCAGTATAGTATTTCATGTCTCTCTATGTTGCAGTTGTTGAAAATAAGTTCAGCGGATCTCATTGCGTATGGGAATTCGTATTTTTCATCTCGTCTTATT<br>GATGCAAAGAAACTGCTAAAGCTGAATAAAGCTTTCAAGATTAGATTGGGAAGAGGGTTATATGGAGAGTGCCTGGTAATTGTTTTTTTTTTGTTGCATCAAAATTCTCTTTGTTTCAAAAATCACTGATTGAGAATTTGTTCTAATC<br>ATAGGGAATGCGCGCAGATGGTAACCATCAACTAAGCGATGAACTTGGCAAGCTTCTTAGTCTACAAAGTTCTGCAGCTGGTCTGAGGTAAATATAATTTCCTTATGACGCTTCTGTTGTTGTGAAGAAATGTGGATAGTAGTGTA<br>AGTTAGTATCACCTCTTTGATATCAGGCCTATAGGAGCTGTTACATTTGTGCAAAGGAATAATCTCAAAATGTGCTTGAGAAGTACCGATGCTATAACCAATACATCCGAAGTTGCTAAGGTTTGTCATGTTTCGAAATCTTACTG<br>CAACTTTTTGTTTAATGGTTTTAGTGATCAGTTAAACTTATATACCTTCCATGTTTTTTAAAATTTGGAAAAGGCTTATGGAGGAGGTGGAACGTCTAGTTCAAGCTCGTTTATCATAAGAATGGATGAATATAATCAATGGATTTC<br>GAAGCTTTGACCATGAAAACTCTTCTCCAAGACCAATCTCAAAGCGAAATTTTGGCATTGTCAAAGACATTAGACTTAGAAGTCAATATCTAGTGACAAGTTTGGTGATCACAAACGGCTGATAAATTATCGAAGATTGAAGTTG<br>AAAGTTGAGTCGATTCCTTTCTTGAGCCGGTTTTGATTTAGATTAGTTTGGGCACAGCTACAGCTACATGTAAGGCGGTGAAATCGAACGGCCTAAAATTGAGAACGCAACAAGTGTACATGTCTACTCCAGTTATTCTTGATTTT<br>GTTTATTCTCTTTTTTTTTTTTTGTCAAAGTATCTTGAGCCGTTTTCTCTGGATTTTTATTTGACTCGCTCTTCTTTTCTTGCTATCCAAAAAAGGTTAAAAGAAGTTGGAAGCTTGTTTGGCTTCAGGTTTAAGACTATCATCAATCAG<br>GCGTTGTTACGAAACCCGCTAAAAGAGAAAGAGGGTTATAGTTATTTAGTTTCACTCGTTCGCGAAGTCCCACATCGCTAAGAAAAGAACGTGAAAGAAAAAACGAGTGGTATAAAGTACGAGGCCATGGCACTCAGCAAATCA<br>TACCTTTCTCGGCCTTTTGGCTAAGATCAAGTGTAGTATCTGTTCTTATCAGTTTAATATCTGATATGTGGGCCATCGGCCCACACGATATTAACTCTATTTTTTAAGGGAGGAAGCCCGTTTAGATAGCTTGCTATCTGGGCTTTCA<br>CGAGTCTCCCATGCGTTGCACTATTGCGAGGGCTTGGCTCAACCCGCCATTTATTCAGTCTAAGGAAATAAAAAAAAGCTAAAGTGATTAAATCGAAAAGCCAAATAAAATCCATGAACGTCCGTCTTTCTTGTATAGCCCATGA<br>CAGTTGGGCTTAAGGAGCCCATAATCAAAACCCATACAAAAGATGTATATATTAGTACCTAAATAAATTGCAATTCGACCACTCTGTTTCCTGAGATTTATCCTCAGTACCCGATACTCTAAAATTGACTTACCTGAAATTCAACA<br>TAACGATTCTCAAACTTCCACGTGGCAATATCACGTCCTTGCACTTGGTCTTCACGATTTTCGTTTTTAAACGACGTCGTTTCAAAGAGCATTATA
OsmtHsp70-2	Os03g02260	GGTTTAAGGAACAGAATGAAGTAATGCAGCGTTCTATTCAATCAACACAACAGCTGTTACATACTTCTATAAGGAACACAGTTCTACCTGGAGGACTAGGGCCCAATTATAGTATGCATCTGGATCTTCCGGATTTCTTTCGATGG<br>CACTAACATATCTGCAGAACATATGTTTAAAGCACAATCGTGAACTAATTATAAGTTCATAACGTCAGCACAGGATTAAGTTAATCAAGTCAGACAAACAATAACCTTTTGGCAGCAAAGGAAAGAATGCGTTGACGAGAACGA<br>CCTTCCTCGTCAACTCCAGTGACACTATTTATCAAGTCCATGGCAACATCATGATGGGATGAGAACTGTAGGCTGTCCTGGCTGAAATGGAGCAAAGAAAGTGCTGCGATTCGTAACATGGTAGCTAGTTTCGAGATAATTTGGA<br>GAATTGAGGATTAAAAAATCTATCTACGATCTCATCAGTGGTAATAGAAAAAACAATTCTAACAAATAAAATAGTGTTGATATAATCATATAAATAATAATATTGATAATGAAAACCCACGAAACTGCATGGTGGTGACTTGTTG<br>AAATGAAACATTTATTGACTAGATAACTGCAAGTGTTTACATTTAAGTACAAACCAGGCTCTTAGGATATAGTTGGTAGGAATATTTGTCATGTACAGGTCAGGGGGAAATCCAGATGGTTTTTCCAGAGTTTAGCACTGTCGCGA<br>CAGCCAATGTTAAACAGGACAAGACAGCCTTTAGTGCTCAAGCCCTGAATCCAATTGGGAAGCTAAGCACGGTACAATTTACTAGTTTTTTCCCCCTTCCTGCATACCAGACACAACTATTGCACTATGCAAGCATCCACGATATG<br>TTAGGTTTGCTAAAAAAAATCAGAGTGCCGAATGACTAATAATAACAGTCATCTGATTCAGCATGTTATTTTAGCCACATCCTAAAAAATGGATCCGCACGTAGCAACTTGAAAACTTCACACGATCAGTAAGCTAGTTCATCGG<br>TACAGGGTGAAGTAGGAATACGGCATTACAACCCCTGGAAGAGACTACGTGGGACTTGAATTGGCTCGAAAAATAATATAACTGACGCAATCTCAGACATATCTAGAAGCCATTAGGACGAGCAAAGCCACTAACCAATCAGAT<br>CACCAGCGCCTTCCTACGAAATGTCAAGCACTAAACAATTCGACGAAAAAAACTATCGAAATGTACCACCACGCAGTTCACGCAAATGGCAGATCTGAGGGAAGGTGTACAGCTGAGCAAGCAGCGAAAGCTGAGACGCAGCT<br>GCACAGCTCCAATGGCTTCTTCCCCCCCCCCCCCCCCCCCCCGAGTCCCGAGATCCGAATAGCTTCGTAGAAACCCAAAGCGAGAGCATAGAAACTGACCTGGCGCGCTCCGCACCGGCAGATCCGATCCCATTGCCCTCCGAA<br>AGCGTGGATCTCCCCGACGAGGGCTCCCCGCTGTCCTTGAGCTCCCCAAGCAGCTCCCGCATGGTGAGCGCCCGCGAGGGCTCGCCCTCTGCGCCCAGAGCCGCGTTCGTCTCCGAATCGTTCGCAATTCCCGATTCCTCCCCCTC<br>CTCCGGAGCCTCCCCCGCGTCGGGCGCCACCACCGCATCCTCCGCCATCCCCGCGCCGTTGGGGAGCTCCCCGGAGTCAGCGGGCGTCGGCCGCGCAGAATCCTCCATGACTCGAGTCTCTCCTCGGATTAGGATCTAACTCGGC<br>GGGAGATCTGGGCGATGCGATGCGATGCGATGCGAGGAGGAAGAGGACGCGAGCCGAGCCGAGCCACTAGTGGTCGCTCCCGCTGTGAAGCGGATCGTTCACTGGAAAGAAAAGAAAAAAAAGGGTTTACGTGTGCGACCACT<br>GGGGCCCACCCGTCAGAGTCTCATTGGTGTAGTTGGGCCGGGCCGTGGCAAACCTGGGCCGGCCCAAAAGTGGAAGGGGTCCGTACCTTCCAGAGGCTTCTTCGCCACCAAATCCCA
OscHsp70-3	Os03g16880	GATAGATTCTTCCATATTTTTATGGTGTATATTAGAAATAACAATTTCAATGTTAGCTGCGTTTTTTGCGCACTTTATAGATAATATATTATATATCACATTTCAATCATAAAATTACATCTAGAGGTTGTATAACCAGCAAGCTCA<br>GGCTTTGTTTAGATCCCAAAAAATTTTGGCCAAAAACGTCACATCAAATGTTTGGACACATGCATGGGGCATTAAATGTGGGGAAAAAACACCAATTGCACAATTTGCATGTAAATTGCGAGACGAATCTTTTGAGCCTAATTAC<br>GCCATGGTTTGACAATATGATGCTACAGTAAACTTTTGCTAATGACGGATTAATTAGGCTTAATAAATTCGTCTCGCAGTTTACATGCGGAATTTGTAATTTGTTTTGTTATTAGTTTACGTTTAATACTTCAAATGTGTGTCCGTAT<br>ACTTAAAAACTTTACACCCGAAGAACTAAACACACCCTCATTCATGAAGGTAAAGTAGTTTTTTGCATAATTGGTGGTACTTTTATGTCAGAGTGTCAGACATAGAAGACTTGTAAAAGGGGTGATTGTGCAAACTATTTCAAAA<br>ATATGATCATACAGAGATCTAAATAGAGAATTCCTCGACAACTGAACTAACAGGACACATTGCATTCTTGGTCAGCCTTTGAGACAAGCAGCATACGGAATTCTGGCTCTACTAGATTGATTATTCTGTCTACCTCAGATATATAT<br>GGGATAGGACCATAATGCATCCCATTTCTTTACCATTTGGACAGCTATCATCGCAGTTGCACATTTGGGTGACATCTGCATTATATACAGATGCCTGTATTTTGACTATCAACATTCTGTTGGATTATTTAAAATGCTCGATATATT<br>GCTTAATTCACAGTCCACTCAGCACAGTCAATGGGCAATTTTATCATCTGTAGAGTTGAGCTCTAACCTGTAATGTTTCTCCTGACGCAGGCTCTTCATGGGTAATACTTAGCCGATCATTCCTGGAATTTTGCTTATTGGGATGGG<br>ACAACCTTCCTCGCACTCTACTCATGTATTTTGCCAACTTCTTGGCATCCTCGGAGGGCTACTTCCACACGGTGATCTGCAACTCAAAGTACTATCAGAACACTACCGTTAATAATGATCTCCGATTCATGGCATGGGACAACCCTC<br>CACGAACATTACCTGTTAACCTGACAACCGAGCATTTTGACGCAATCGCAAGCAGCGGTGCACCATTTGCACACTCCTTTGCCAATGACAATCCAGTCCTGGATATGATCGACACCAAGCTGTTGAGGAGAGCACCTGAACGATT<br>CACACCTGGTGGATGGTGCTTAGGTAGCTCGGTAAATGACAAAGATCCATGTTCCTTCTTCGGAAGGTCTTTTGTTCTGAGGCCGACGAAAAGTTCGGCAAAGTTAGAGAAATTGCTGTTGAAACTTTTGGAACCTGATAATTTCA<br>GGTCTAAACAGTGTATATAACACATTCTTGTTACATGTAGCCTTGTAGCCAGTTGTAGAATTTTGATAGTGTTCAGAGGCAAGTGTGACCGGAGGTGGAGATGGTTTCTCATTGTTGCGGCTGTAGGCACACAGTGACACAGCGTG<br>TCGTTGTACATTTGCAACTGGAAGGTCAAGTAAAGTGACTGAAATCATACATTTACACAAAATTTTCATCTCTTTCATGGATACGAATACGCTGCACAATGTGCCATGGAATCAGGTACCCCAGTTACAGTGGAGTCAGGCCGAGT<br>TATGGGCTTGCGGCTGAATTTTTCGTGTAAAAAAAAAGGGTAAAAAAAAAAAGAAACGCCTACCGAGTACCGACTGTTGATTTCGTGAATCCTTTTAATGCAGCCTCGTAGAACGCCAATGTTCGCTTCACCTCCCAATTGCAGTT<br>CACACTTGTGTGTATGCTAGCAAAATGGCTTCCCGCCTTCCTCTGATTAAAACTGCATGTATATCTGTATAAATGCGGCACAATCCCCTGTACTGATCA
PpcHsp70-2	Phpat.003G105300	TAATATAAACATTATCTTAGAAACAAATATTGAAGCCATTTATTTGCAATCGATTTCAAATGACAAGAAATAAGAGTGACTATAGATCTCCAAGTTGGCCTTTAAATGGAGGCAAATTTATATTCTACATACATATACATAAGTAT<br>TCTTTGTGCTTTGTAGAGGAATAGTGAATGGAAAATAGAAATCAAGGAATAAAGAACAAGAAATAAGAATAACTAGGTAAAGATTTCTTTTGGCTATACACTTGCACATATGCTTTGATATGTGGATTAGATCTTGAATCAAGTG<br>GTATTTTGTTGATTTCGACAATCTAATACAATTAAACGCCACCAATATGAATTTTACTCATTTTGGAGTACCTTCACACGTCAATCTTTGGGTGACTCTCTCACCAAAAAATGTTTTCAAAGAATTTTTGGAGGAAAAAAAATTCTTT<br>TCTCTTCCAAAAAGTTATTGTACTCTTAGCTCCTTTGTAACATTTTGTGAATATTTCTTTTTGGTCTATTCTCTTCTTTTGATGTTTTTTAATAAAGTTATATTTTTTAGTATTTTTAGTATGTATGTATATATATATATATATATATA<br>TTTTCATAAGATACATGTGTGATAAATTAAATGTTGTAACAAACTTGTTAAACTTTTGGATGTCATATATTGTTGTTTACATAACATTCAAAAAATCTATTATCATTTTTCTCATTCTATCATTGATACAAAAGTTATCATATTTGAC<br>AAATATTTTAATTTAAGAATGTTTTAATTTAAGGATGTGCTAAGGAGGCTATTGCTAGATATCTATTCTTTATATCCTCAAATGTTTTGACCTCTTTTGTTATTAGAGCAATGTAAGATGTAGCTTTTACGTGCAAAACATGTGTATT<br>TGTCTATTATTCACTAAACTACAATAAAGTACTTGATCATATTAGAATGATTCTAATTACAAATATTATAAAATGTGATCCTTCTATAATAATCTAAATGTGATGATGTGGGTTATATTGATTTAGACTTGACATCAAACATTCAT<br>TAATAAAACTGAAATTATAAAACTAACACTAGTCTCTAAGATTATCACATGTAATTGGAACTTGACCAACTCCCATCAGCTACCAAGCTTGATGTTGGATTATGAAAGGGTGAATAAATCACAATGAACTACTAATGGATGAATG<br>CATAAATGAGCTAAAAGAGTCCTTTTCCATGGGGGGAATAAAAATGAGAGATACAAAAGAAGTAATAGATTGTGTGGAAAGGGAAGCAGCCCCTTCTTCTTAAAAGAAGAATGTTACCATGATGGCTTGGTCGGGGATAGAGGT<br>GGGGGGGGGGAGGAGGAAGGAGCAGAAAGAAAGGTTGGATGCGGTGGAAGGGGGAGTGGGGGGGTTCCGACACATGGCGATGCACATGGGGATCGGGGGGCGTTGAGGTGGTCGGCTGCGACGCCCCCAAGGCCGGATTTGG<br>ACCCGCTTGACGCACCGACTGGCAGTGCGAACCGGATTTTGGCCATTGTGGCCTGTTTTCGTCTATTCTGGACACTAGACCCGGACTTCACCCCTTCCTTCCTTGTCTTGATTTTCTCAAGGTACGCCCGGGCCTGGTCAGAGCTTA<br>GGGACGGACGGAGGCCAGAAGAAGAAGAGTCTGGAAGCCCCTGGAACTCCCCCAGCGCTCGCGGGTGCCCGGTGGAGGGCCGGGTCTGGGGGGTGAGTCATTGGGTCCGGAACCCTCTAGAAGTTGCTGGAACGCGCTCGAAC<br>CCGGCGAACCTTCAGCTGCGAGCAGCCAACTGTTGATTCGGCCGATATCGTCAGCCTAGAAAACTCAAGCCTCGCTGCTCGCTCTCGCCAATTGATACTTAGATTTGTCTCAGCATCGCGGCTCTGAACTTGCGAGCGTCCGTCTC<br>CGTCTCTCTAGCTACCAGTGCCCTTTAGGTGTTAGATTTGGTTTCCTTTTTCTAGCGTGTTGCTGTTGCGTTCGAGGGGAGTTTTCTAGTGAGCGTCGCCA
PpcpHsp70-2	Phpat.004G105700	TTCATCGTAAATGGATGTGTATTGAAGACTGGGCTGATGACTGAAAGTTAATTGCTAATAGCCACGTGTACATTTAGGAATTGATCACGACGAAGGGTATATTTACGCACACATGGCGAGATTTTTTTTAATTCAATGCCGCACCG<br>CTTCATCTGAACAAGACAAACTTTATATATGTCATGAATGCTTCCATCACCTTCATAAAAGAGCGTTTTTCTTGATGCCAATGACCTGATTAGACAATAATACATTTGAAGCAATTTCAATCAGATCTTCTGCACTGGTTGCCTCGA<br>TATCATTAGCTCTTGGGTATTCTCCATAGAATTACGGTTTACAAATCTGAAGCAACAGAAGTAAGTGCCTCTGGAATCAAAATAAAAATCATAAGAATAAGAAAAACAGTAAGAGATTCGCTTATTCGCAGATTACTGCTATGTC<br>AGAAACGCTCTGCCGGGTACGATCGACGAGGGTACAGCTGGCTGTGAAGCTGAAAATTTCCGGCCCTCCAGCTTTTATTCTTGGCTTCTGCTCTAACCAATACAATGCTTAAATGCTCTGGAGTAGGTTAACTACAAAGAAAATGC<br>TGTTTCACCCTCACGTGTTGTCACCCACGTCTGGCGGGCACCATAGAAATGCGGGACACTAACTCCCTCCTCCCTCAATCCTCCTCATTTAAGGTCACTCTCATCCAGGACTGCCGTTTAAGTATGATTTGCAGGTTACCTGTACTC<br>TGGTGGTTTCTGATATCCTCGATTAAAGATGTTTCATCCTCCAAATAAATAAATAAATAAAATAATAAAAAACTACCACTCCAGAGATTGAATATTTCCGATGGGAGAAAGGGTACCAACATACCATTTCAGTAAATGAAAATTG<br>AGCTTCAGGATCTTCTGGTTGGAATGTTGTTTCCTCTTCTAACTGTAGGCTTAGCTGTGTGGTTCTAGTCTGCCGCCATAATGTTGTTCTCACATGACTCTTATATTGTACTTTTGTTACCGTTAACCTTTTTGAATCTTCCGTGTTTCAT<br>GATGAGGAAGGCATACTCTCTGAATTCCCCCTCACTTCGATCTGCCAAACAACTGGAGTCTCCTCTTTGAAAGTTAAGTCTAGCTTTCCTCCTTACATTCACGCCTTCAGACAGTACAGACCGTATCCACAATACTAAGAACCTG<br>TTTCGGAACCCGAGAAATTTCTACATAAGCGAGGACGCTGCACTAAAAGAAACCATTCATGTGGACGGATCTTACCAACTTCTGCAGTTTAACACGAGGCATGTTTTAACAAACTCAGTAGAAGCACCGAGCGGACTGAGGC<br>CTGGAACGAAACCAACCTTGTTCGTTTCCTGTAAAATCTGACTACAGATGCAACAAAGATATCACGGATGATCCAGTCGAGAATTACTGTCTGACGCTCATCCCAGAATCGGCACACCGTAGTTTTGTGATATGTTGCACTACT

		CTCCTACCCATCTGCGCACCAAGTGTTCCAGAAATTGTCTCGAACGCAACAATCTAAAAATAAGAGCGGTTCCAACGTAATATGGGAACTGTTGTGAAAGCTAAGTTTAATCGATAACAACGAGCAGTCCTAGCAAGGGAGGGTT<br>CAAGAAGACATTCAGAAATTACTAGTCTCTGTTGTTGTTATTGTTTACGAATCAAATATCATTTTAACCCACCACAACTGAGATTTACGGGAGTTTAGAGCTGAGATTTCCCAGAACCAATAGGAACCCCAACAACACAAAGTTGC<br>ATCCGCGACACAACCCATGGACTCGCAAACTGTAGCCACCTGAATGAGACATTGCTCTTCGGAGATACGGAAGCTTGAATCTCGTCGACACTGCGTATTCTCGATTATTTCGGAAATTTCTGGAAGGTCGTTCAATTCTAGAAAAT<br>GATTTCCACGTTGGAATGGATCCACCACAATCCTCCACTTGGCAGCAGCAAGAACTGGAGCTTTCTTCGTCCAATTCTTCGCTTGAAATCCATCTTTTAGC
Salt		
Atc<br>Hs<br>p70<br>-1	AT5<br>G025<br>00.1	TCATTTTTCGTAGTGTTTCAATCATTCAAGTTTTAGTATGCAAGTTTTCTGTTTAGAGCATATTGCATACATGGGCGAATATGGTATACCTGGATTTCCCAGTTCTTCAGGTCTTCTCTGTAGCTCTGTTACTTCTATCACAGTTATCG<br>GGTATTTGAGAAAAAAGAGTTAGCTAAAATGAATTTCTCCATATAATCATGGTTTACTACAGGTTTACTTGATTCGCGTTAGCTTTATCTGCATCCAAAGTTTTTTCCATGATGTTATGTCATATGTGATACCGTTACTATGTTTATA<br>ACTTTATACAGTCTGGTTCACTGGAGTTTCTGTGATTATGTTGAGTACATACTCATTCATCCTTTGGTAACTCTCAAGTTTAGGTTGTTTGAATTGCCTCTGTTGTGATACTTATTGTCTATTGCATCAATCTTCTAATGCACCACCCT<br>AGACTATTTGAACAAAGAGCTGTTTCATTCTTAAACCTCTGTGTCTCCTTGCTAAATGGTCATGCTTTAATGTCTTCACCTGTCTTTCTCTTCTATAGATATGTAGTCTTGCTAGATAGTTAGTTCTACAGCTCTCTTTTGTAGTCTTG<br>TTAGAGAGTTAGTTGAGATATTACCTCTTAAAAGTATCCTTGAACGCTTTCCGGTTATGACCAATTTGTTGTAGCTCCTTGTAAGTAGAACTTACTGGGACCAGCGAGACAGTTTATGTGAATGTTCATGCTTAAGTGTCGAACGTA<br>TCTATCTCTACTATAGCTCTGTAGTCTTGTTAGACAGTTAGTTTTATATCTCCATTTTTTTGTAGTCTTGCTAGTTGAGATATTACCTCTTCTCTTCAAAGTATCCTTGAACGCTCACCGGTTATGAAATCTCTACACTATAGCTCTGT<br>AGTCTTGCTAGATAGTTAGTTCTTTAGCTCTCTTTTTGTAGCCTAGTTCTTTAGCTCTCCTTTTGTAGCCTTGCTACAGAGTAAGATGGGATATTACCTCCTTGAACGCTCTCCGGTTATGACCAATTTGTTGTAGCTCCTTGTAAGTA<br>GAACTTAGGATAGAGTGAGTCAACTTTAAGAAAGAACCTAGTATGTGGCATAACCAGATTGCAGGCTCTGTCTCGGCTACAGTAACGTAACTCTATAGCTCTTTGTTTTGTTCAGAAAGAACCAGTGATTGGATGATTCGTCCTTA<br>GAAACTGGACCTAACAACAGTCATTGGCTTTGAAATCAAGCCACAACAATGCCTATATGAACCGTCCATTTCATTTATCCGTTTCAAACCAGCCCATTACATTTCGTCCCATTGATAACCAAAAGCGGTTCAATCAGATTATGTTTT<br>AATTTTACCAAATTCTTTATGAAGTTTAAATTATACTCACATTAAAAGGATTATTGGATAATGTAAAAATTCTGAACAATTACTGATTTTGGAAAATTAACAAATATTCTTTGAAATAGAAGAAAAAGCCTTTTTCCTTTTGACAAC<br>AACATATAAAATCATACTCCCATTAAAAAGATTTTAATGTAAAATTCTGAATATAAGATATTTTTTACAACAACAACCAAAAATATTTATTTTTTTCCTTTTTTACAGCAACAAGAAGGAAAAACTTTTTTTTTTGTCAAGAAAAGG<br>GGAGATTATGTAAACAGATAAAACAGGGAAAATAACTAACCGAACTCTCTTAATTAACATCTTCAAATAAGGAAAATTATGATCCGCATATTTAGGAAGATCAATGCATTAAAACAACTTGCACGTGGAAAGAGAGACTATACG<br>CTCCACACAAGTTGCACTAATGGTACCTCTCACAAACCAATCAAAATACTGAATAATGCCAACGTGTACAAATTAGGGTTTTACCTCACAACCATCGAACATTCTCGAAACATTTTAAACAGCCTGGCGCCATAGATCTAAACTCT<br>CATCGACCAATTTTTGACCGTCCGATGGAAACTCTAGCCTCAACCCAAAACTCTATATAAAGAAATCTTTTCCTTCGTTATTG
Atc<br>Hs<br>p70<br>-15	AT1<br>G799<br>20.1	TATGCGGAACAAAGTGAGAATCTTTATTTCGTCTTTTCATTGGCAGTATGCATATGTAAAGTTGTGAGTATTGGGTACTAACGTGTCTTGTTTTCAGCTGAGTGACAAATACCAGGAATATATCACTGACTCGGAGAGGGAAGCAT<br>TCCTGGCGAATCTGCAGGAAGTTGAGGATTGGTTGTATGAAGATGGGGAAGATGAGACCAAAGGTGTTTATGTTGCGAAGCTCGAGGAGCTCAAGAAGGTAATGCATGCATATGATGACACTTTATATTGTGCAGGAAGAAATC<br>ATATTTCAGGATAATGTTTTAACAGAGGAGTGGGATTAATATTTTGCAGGTGGGTGACCCTGTTGAAGTGCGTTACAAGGAGTCATTGGAAAGAGGGTCGGTCATTGATCAGCTTGGTTACTGTATTAACAGCTACAGAGAGGCA<br>GCTATGTCTACTGATCCCAAGTTTGATCACATTGAATTGGCAGAGAAGCAAAAGGTAATAATTTGGCTCAATCTTGGAAGCACAAAAAAAATCTTAAATTTCTCAGGCTTGGGGAAATTGTTTGATGTTTTGTTGTTGTGAAATGA<br>TTAAGGTTTTGAACGAGTGTGTGGAAGCAGAAGCATGGCTGCGGGGAAAGCAGCAGCAGCAGGACACACTTCCCAAGTATGCCACACCGGCTCTCTTGTCAGCTGACGTTAAAAGCAAGGCAGAGGCATTGGACAAGTAAGCA<br>AGGCTCCTTGTGTCTTTGTCTTTTAAAAGAAATGCTAAATCTGATAATAGAGAGTGAACCAGCTATGGATGTTTTACAGGTTTTGCAGGCCTATAATGACCAAACCAAAGCCGGTGGCTAAAGCAGAGGCACCACAAGCCAAGGG<br>AGGTGAGCAGGCGGATGAGGGCAAGAGTGAGCCAGAGCAACCAGCTTCTGCCGAACCAATGGAGACTGAGAATCCCGCGGAAGGTAGTACCTAAATGAATGACGAGAGAGAGATGGTGTTGTATCGATTTTGTTCTTAACTCTT<br>TTTGATTTACCTAGTGGAACCTCTTTTTCTGGTTTCGCTATTATTCCCGGAAAAAATCTATATTGTTAATTCTTCCTTTTCGAAAACAATTTTTAGGAAGCATCTTTTTTCTTATTGTTCCTTGTGGTTTTGTTTTTAATTTGAAAGTCA<br>TTTTTTCTGCTCCTCTATATAACTATTATGGAGTGTACACGGATTCGTTCTATGTTGGACTGGGCTGTTTTTATAAACAGCAAATGAAATGCAATTTGGTTCTATATATATTGGAGCTTTAAAAGGCAACTTTGAACCCCCTAAGCTT<br>AACATCTTTTCTCTTATATCTTACGTGAAATCAGTGTGACAAAACTCGTCTTCCTCACTTCTCTTCCTTACCATTGTTGTACAAAGAACATCTTCCTTGATGAAGCAGTCGTAGCCTTTGGCAACATCTTTGAGGTCATCCAGGCTGA<br>AGTTGTATCGTTTCTCCAATCCAAACGGGGTTAAACAAGGATGAATTCATTTCCTCGTTATGAGCACAGAATGAGTGCGACTGCTACGATATGTGATTAAGCTGCCTGCACCAGTGAGGACAATGAAAATCTCTAACATAAATCC<br>ATGAATCATCTCTGGGCGCCGCAGATAATGGCATGTTCCAGACACCACAATGCAATTAGTTCGTGCATATCTTATGTACCACAAATTCTGATAACACCAGCCACTTCTCTATAATAATTTTATTAATTTTAAATACATTGGGTTTAA<br>ATAATTATATGTAGACTCGAATAAAAATATACAAAAACACTGGGATAAAAAAAAAAAAACCTAAGAAAAAAAAAAGGCAATAAATAAATAATTTAGGCAAAAATATCACAAAAATAAAAAGGAAATGAAAGTGTTTTTAAAATAA<br>AAAAAAAAAAGGAAAGGAGAAGAACATTCCAGAGGAACCCAAACCCTGTGGTGGTGTCTGCTCCTTAATTAAAACCTCTTCTCTCTTGCTCTTCCTTTATAAAAGA
Atc<br>Hs<br>p70<br>-8	AT2<br>G321<br>20.1	GCTATACAAGCGGCGGAAACGGTGGTAGTTGATGTTAAAACTTTATCGGAGCTTATACGGAGTAGTAACGGAGGAGGTGGTGGAAGTGGCAGTGGAAGCGGTAGCTCCCTTGAGAATCAGCCGAGGAGATCAAAGGTGAGGAA<br>GCAAGTGTGGGCTGAAGTTCTCGGCATCTCATCCGACTCCACCATCATATTAGAGTGTATATAGTTGGTTTAGTTATGGTTTAGATTCGTTAGTATTTTGATTTAGTTTACTTGTATTTAAGTTTAATTATATATCATTTTTACACTTA<br>ACTAGATATTGACCCGCGATACACCACAGATTGATATTTTCATTACAAGGTGTATCATATATTAAAAAAACAATAGCCTATATATTAAGGGTAACAAAATAATCAAATGATCGTCCGTATAACATATGTTTTACATGTAATAGAGT<br>TTCGACGTCGACGATGAGGAAGGTCTCCTCCAACAATGGAGTCGGAGTCGGTACGTTTAAGTCACAGAATCCAAATTTCGACGATGAAGATGACGACGGAGACTGCCTTTTTAAAGCGGTGGTTGCGAAGATCTTCGCTAGTACA<br>ACTTCCATTAAAGCTGCTTACGCAGAGCTTCAGAGGGCTCAAAGTCCGTACGATAGCGACGCTATACAAGCGGCGGATACGGTGGTAGTTAATGAACTAAAAACTTTATCGGAGCTTAAACGGAGTTTATGAGGAAGGAATTG<br>AATCTTTCACCAAAAGTTGCGATTATGTTAGCTGAGATCCATGAACAGCAGAGTTTGATGAGGACTTATGAAATCGCGATGAAGAGACTTGAATTTGAAGTTACGGAGAAGAAAGTGAAGATCGATGAGTTGAAGATGAATCTT<br>GAAGAGAATTTGGTGATGAATAAATCGTTGGAGAAGAAACTCACAGCTACTTGACTCATCGCTTGAATTGAAAATTATTGTATTTTCACTAACTTGTTGGACTTTAGATATGAGCCGAGCTTGCTTTTAAGTTGACATTGAACGAA<br>TTTTTGTAGAAATTTTGCAAAATAACTATTCAACGTCCGTGACCAAGAAATTTAAAAAGTTAAGTGAGACAACCCTATTAATTATCATGCTAATATTGTTATTTTATACACCAAAAAACCAGCATTGTTGCCTTGTTGGCCACATAA<br>ACATCAACCAAGAATATAAACACATTATGCAATTTTTTGAAAGTAAAAGTCTCCAACAATATTAAAAACCAAAGCTAAACAAATGATCATTTACCATACTGTATATAATTTGGAACTCTAGCGAGAAGCACACTTGATTTCATGG<br>CTTTCCGAGAGCCTATACTTCATAATGACTATGCCACATATATAGCGCAATTCTTGATTAAATCGAATGACTATGGTATATTGGTATAAATTTAGGTTTGAGCCTTTATGACCAGATGAACATTCTAGATACTTCTTAGCACAATCA<br>TGTACATATAAATCGATAAACTGGAAATTGTTGAGAGCTCACGTCTTAAAGCATATGTTGGCCAAAAACGCCAATCACGTAAGATGCATAATTTATGCATCTCTTAACGTTGATTACTTTGAAATCTCATCTTGTGATGATGAAAT<br>TTAATCCCTTAAGGCGAAGAGAAATAAGCACAATGACCAAGAAAATCCTCTTCTTAAACTTCTTTGACGATTGGAGTTTCTGATGCTAATTCTTTGATATTCTGTATATGCATTGGCCAATTTTCATATATGTAATTAATAAACACA<br>CTGATTGATATACTAATTCATTGAAAAATTTAGAGGAAAAGAACTAATTGAAACAAATATTATAATTTATGATCTAGAGTTGGGCCAGTACTATTCTACAGGCCCATATGATTTGTTAAAATTAATATATATATATACAAGTTTACTT<br>CAAATGGGCCCATTTTTTAGAGAAAAATAGTAAAGTAAATTATTTTCTTGGGTCGGCAAATTAATTTAGCGAGAAAAAAAAAAGGAACTCCCTCCTCTTTTCTCTG
Atc<br>Hs<br>p70<br>-4	AT3<br>G125<br>80.1	TGATTAGTGTGACTGAACCTAACCGGTGCGTTTGTAATATAAATTCTTGTCATTATTTTGGCTGCCACTATCTGATCTGAGAAGCCACAAGGAGTTGAAGTTTACAGTTGTTAAAATCAATTTGACAAACAAAAAATCAAGAGAGA<br>AACATACAATTACCAATTTGATCCACAGATAAATCAATTGTTAAAAAGTAATTAAATACTACTATTGGGCCAGGCCTGCAAAATAAAGACCCTCAGACATCGAAACCCTGAGAGTGAGATCCAATACTTGTTCTGTTCTGCAAAT<br>CGCTTCTGGTCCCTGGAGTCTGGAGATCTCATCCTTTCCCACCGATTCCGTCCCGATAAAACGGTAAATCTCGCTCAATTTTAAAACTATGTATATATATGCATTTCTTCTTGAGTGATCTTCTGATTCGACGACAAGTGTGTTTGAT<br>TTATGATCTGGTTAGTCTTAGACTCTTAGCTGTTCTTCTCGCGATAGAGATTGTCTCTGTGGCGGCCATCTGTGTGTGATGGTCCTTTGAATCCGTCTCTGTCGACGAGAACTGCCAAATTCTCTTGTCGGATTGTGTCACGTTTTAG<br>GTTTTTACATAAATTCTCAACTTGTTCTCCAATGTGATCCCATAGCGGAGTGGATATCGCGTTAGACTCCGAATCTAAAGGTCGTGGGTTCGATTCCCACTGGGATCATCCCATTTTTTTGGTCTTTTTTTTTGTTGGTTATTAATTAT<br>TACTATCAATCCGTTCTTTTAGCAGTGATCAAGCGAATTTAGGTCTGGCTCTGCCTCTGGCTCTAGTTGAGTTTAGGGAGCTGCTCCTTGTTGTTTGCCATTATGGTTTCTCTACTTTGGAGTCAACTTTGCAATGTTACCCTTGTAGT<br>CTCTTCTGTGGACTTTGTATTTGGTGAAATTGAAAAGTTTAGTAGAAATCTGGCTGCGCCAAAGATGATAGAGACTCTAATGGGATCCTTATTCGAACATTTTGCTGAACTGATTAGATACAATAAGGCAACACTGATTCCATTAC<br>TGTTAGTCTGTTACTACAAAAAGAATTGTTGCTCAAGTATTGTTTGTGTTGGTTGGTGCAGATGATCGATGATCAGGACTTGGGGTTTATTGCCAACTTTCTTGGCATCTTCATCTTCGCATTGGTAATTGCTTATCACTACGTAACT<br>GCTGATCCCAAATACGAAGCCACTTGAGTGATGATATTTTAGAATGATGTAAGGCTTTTTAGTTTATACTAGTATTATCTGTGTTTCAAACTGAGAAGAGATAATAACAGTCTTTGTTGAGATGATAATGTTTTCAAGATGTTCCTA<br>ATCCATTTCACATCTTCTCAATTTTATATGCATGTGCATATATATGTTCCCTCCAATTATGTTGTTCGAATGTTTGATGAAACTTTGAATTTTTTTCTTTAAGCAAAAAAAAATCTCAAACACCAAAGCGAGGAGTCATTCTAGTTCA<br>GTTTTGAGTCATTCTAGTTATTTTTACAAAGTTTTGAGTCAAATTGGGTAAATTTTTTGGTTATTTTGGTCATAAAAATAACTAGATTATCTCTTATATCTTATGAGTTAATTTGGTAAATAAACCATTTATTTGGGTCAAACTATTTT<br>TTTCCCCATATATATATATCCAATCAATAATAAATTCATAATATATTTCATTAACGCGATTGAAATACTAGTAATTAATTGAGGACTAAAGAAAAAGTAATTTCCTTTTTATCTTTAAAATGTGCAAAAAAAACAAAAATGTTAATTG<br>GGTGATGAAATAACTTGTTTTCAAAACGGGAGTTACTATTTGACAATTTAAAAAAGACCCATCTCGAAGGAGCTAGAAGCGATAACAAAATAAAAAGGAAACAATAGTAATTAGATGGCGCAAAAATAAGATCCAACGGCTGA<br>GATCTTTACTCGTGAACGTTCTCGAAAGCTCTTTGCCGACCCACTCTTCATTCATATATAAACAAACACCTCTCTGCCTTCTCTTCCT
At<br>Hs<br>p70<br>-<br>BiP<br>3	AT1<br>G090<br>80.1	AAGTGTAACCATTAGATAGTAGACAACGAACCTCAATATATCCGAGGTTGTCGCGATCTAGTTCTTCCATGATCAAGGCAGCATATTCATCTACATTTTCTTTAATCTTTGATAGCTTGTTAGCCGAAGCACTTAAAGCAATTATCT<br>ATAATTCAGCCGAAACAACTTAGATTGTAACATTTTTATATTAGGACCATAACGAAATTGAGATCAATTTAATAAGGTATACCTCACCTCCTTAACCTCATCTCCTGTAATACGTCCGTCCAAATTTTTGTCCACCCTTATATACAT<br>AAACACAAAGAGATAACTAATCAACTTACCATATTTTATGACAAATGATAATCATGCATTATACTTGTCAAAATACTATAAATAAATATAAAATCATGCTTACATATCAAAAAAGATTTGCAGCCTATCATCAAAACTATTGCCTG<br>TGATCTGCTCCCAGAATTCAAACAATTCAGTTTTGGTGATTGACGATGACGTAGTCCCTCTCCTTCGGACCAACGCCTCAAAAAGCTCGTTCACAAATTCGCTAGATTCAACCATTCCTATTTAGTTGAAATAATTCTTTTAAAATT<br>CAGTTAAAAAAAAAAGTAATAAAAAAACAAAACTGTATATGAGAAACAAGTTGAGTATTGCAGTGAAATTACAGAGAGAAAAAAATGAAACCAAAAAAGGTATATTATTTAAAGTTTTTTTGTTTACCTATGCAATGTCCAAATT<br>TGGATTTAGGGAGTTTCCCTTCAACGGCCAACTTATCAAACCTCATTGCAACCTCATCCCAGCCTCTACCAACGGCGTCGTTTTTGGCAATGAACCTCAACCCTCGAAGAGCAAACATAGCACCGAACGACTTACTACGGTCGAGC<br>CGGCGTGAAGCAAACGATAGCGACTTAACCATCGTTTTCAGCCTCCCGGAAAGAACCGAACGAAGTGTCGCCTTGGCCTTCATTGTGTTGATGGTTTCGTCTCGCACTTCGAGTGTGATCTCGACGTAGTCCTCATCAGGATTGTC<br>TGAACCAGTGGCTTTGCATCGGGAAATCTTGTTTGTCTCACCTTCTGACGAAGATTCCATTTCTTCCTCCCGCATTCACTACAATTACATTATTCACGCTGTTTTGTATACTATGTAATTTATATAATGTCAAAAAAGACGGATTATA<br>ATGCCCATATTACCCTTCTAACAAAAAATAAAATAAAATAATAATATCCATATTACCCTTTGACAATTTCGAGTATACCATATGAATTATAACTTAGTATTAAAATATTGATTTACTCCAACGACATATTTTAAAAATCAAATGAT<br>ATATGGTCAATTATTTAGTTTGAAAGTGAATTAATATTAGAAGCCAAAAACCTAGGGCCGCCATTAGGGTTGGTTTGCCTGTTTTGTTGTTATCCAATATCCAATTCTTATTGTGCATTGATTCCAATACAAATACAACACTGAGA<br>GTAATTAGAAAATGGCATATTTCTTATATATATGTCTGCGTGATAAATAAATGGAGTGGTTAAGCATTAGTCATGTTCTTCCAAATATTACACCTCACCGCATATGCAGTAGTTTTGGCATTACACTAATTCATTAATAACAAAAG<br>AAATTATCCTTATGTAAATTATGACGCAAATCTTTTTATTTTATTAATCGGTACACGAAAAAAATCTTTTAATAATTCATGCATGTGATAAAAACATTGTTAATAATTTAATTTTGATTATAATGATAATTGTTTTGGATAACTATAC<br>ATACATACGAAGAAGTCGTAAAACCTTTGAAAGATTTTGCGATGACGATTTAATGTCACGTGTCTGCTTGTGATTGGGTACTTTACGTGTGTAAAAAGTAAGGAGCGCGCCAACACAAAATAACCCATTAAGCTTACGTGTCAAG<br>AAGTGATTGGAGAGGACACTCTACCGAGGCTAAATACGAATCATACTGAAGCACATATAAATAGACGACGAACTTCATACTCTTCCAAATAAACTAA
At<br>mt<br>Hs<br>p70<br>-2	AT5<br>G095<br>90.1	TGAGGATATCGATCTTCGGCGATGGATGTTACCAGATATCAAAGCTTTCAGCTTTGGTCTTAAGGATTGGCGCTCAAGGATCAATTGTATCATCAATCCTTACATGTACGAACAGGTCCGATGTCTATTCACATTGTTTCCTTTTGT<br>GAAATAATTAAACTCTTATTGAATCTGTGCATGTCCTTAAACCATTCCTCAGTATAGTATTTCATGTCTCTCTATGTTGCAGTTGTTGAAAATAAGTTCAGCGGATCTCATTGCGTATGGGAATTCGTATTTTTCATCTCGTCTTATT<br>GATGCAAAGAAACTGCTAAAGCTGAATAAAGCTTTCAAGATTAGATTGGGAAGAGGGTTATATGGAGAGTGCCTGGTAATTGTTTTTTTTTGTTGCATCAAAATCTCTTTGTTTCAAAAATCACTGATTGAGAATTTGTTCTAATC<br>ATAGGGAATGCGCGCAGATGGTAACCATCAACTAAGCGATGAACTTGGCAAGCTTCTTAGTCTACAAAGTTCTGCAGCTGGTCTGAGGTAAATATAATTTCCTTATGACGCTTCTGTTGTTGTGAAGAAATGTGGATAGTAGTGTA<br>AGTTAGTATCACCTCTTTGATATCAGGCCTATAGGAGCTGTTACATTTGTGCAAAGGAATAATCTCAAAATGTGCTTGAGAAGTACCGATGCTATAACCAATACATCCGAAGTTGCTAAGGTTTGTCATGTTTCGAAATCTTACTG<br>CAACTTTTTGTTTAATGGTTTTAGTGATCAGTTAAACTTATATACCTTCCATGTTTTTTAAAATTTGGAAAAGGCTTATGGAGGAGGTGGAACGTCTAGTTCAAGCTCGTTTATCATAAGAATGGATGAATATAATCAATGGATTTC<br>GAAGCTTTGACCATGAAAACTCTTCTCCAAGACCAATCTCAAAGCGAAATTTTGGCATTGTCAAAGACATTAGACTTAGAAGTCAATATCTAGTGACAAGTTTGGTGATCACAAACGGCTGATAAATTATCGAAGATTGAAGTTG<br>AAAGTTGAGTCGATTCCTTTCTTGAGCCGGTTTTGATTTAGATTAGTTTGGGCACAGCTACAGCTACATGTAAGGCGGTGAAATCGAACGGCCTAAAATTGAGAACGCAACAAGTGTACATGTCTACTCCAGTTATTCTTGATTTT<br>GTTTATTCTCTTTTTTTTTTTTGTCAAAGTATCTTGAGCCGTTTTCTCTGGATTTTTATTTGACTCGCTCTTCTTTTCTTGCTATCCAAAAAAGGTTAAAAGAAGTTGGAAGCTTGTTTGGCTTCAGGTTTAAGACTATCATCAATCAG<br>GCGTTGTTACGAAACCCGCTAAAAGAGAAAGAGGGTTATAGTTATTTAGTTTCACTCGTTCGCGAAGTCCCACATCGCTAAGAAAAGAACGTGAAAGAAAAAACGAGTGGTATAAAGTACGAGGCCATGGCACTCAGCAAATCA<br>TACCTTTCTCGGCCTTTTGGCTAAGATCAAGTGTAGTATCTGTTCTTATCAGTTTAATATCTGATATGTGGGCCATCGGCCCACACGATATTAACTCTATTTTTTAAGGGAGGAAGCCCGTTTAGATAGCTTGCTATCTGGGCTTTCA<br>CGAGTCTCCCATGCGTTGCACTATTGCGAGGGCTTGGCTCAACCCGCCATTTATTCAGTCTAAGGAAATAAAAAAAAGCTAAAGTGATTAAATCGAAAAGCCAAATAAAATCCATGAACGTCCGTCTTTCTTGTATAGCCCATGA<br>CAGTTGGGCTTAAGGAGCCCATAATCAAAACCCATACAAAAGATGTATATATTAGTACCTAAATAAATTGCAATTCGACCACTCTGTTTCCTGAGATTTATCCTCAGTACCCGATACTCTAAAATTGACTTACCTGAAATTCAACA<br>TAACGATTCTCAAACTTCCACGTGGCAATATCACGTCCTTGCACTTGGTCTTCACGATTTTCGTTTTTAAACGACGTCGTTTCAAAGAGCATTATA
Osc<br>Hs<br>p70<br>-4	Os03<br>g169<br>20	GAGAGGGCGTCGGCCTGGATCTGCACGAAAGGGAAGGGCGGGTGGGCGGCGAGGAGAATGACGGAGCAGGCGAGCGAGGCGGGAGGTGGCGGAGCGAACCGACGGTGGGCGGCAACCTCGGCAAGGTGGGGAGGAGGCGGA<br>GGGGAGCGCGGAGAGGTCGACGGTGACAGCCGGCGGGAAGAAGGCGAGCGTGCCGGAGATTGAGCAGAGGAACTCCGCCAGTGGGTCCGTCGTGAAAGACGACGATGACGAGGACATCGGCAGCTTGGAGGGCCGCGTCGGC<br>GGCGAGGATGGGCGCCACCACGGCCAACCTCTCGCCTGCGGCGCCGGCGTTGTTGTTGTCGTCCTCCGTCGCCTTGCCCCTCGTCTGTGCCGCGGCCGCCGCCTGCTGGAGCCCGCGTGGAGCGCCGCCTTGCCCCTCGCCTGCTCC<br>GGCCGACGCCGCTCTGCCGGCCTGCTCTAGCTGCGGCCTGCCCCGCCCTGTCCCACCCCGCTCAGCCGGTCAGCCGCTCCGCCCCAGTCGTCGCCTGCTCCGGCTCGGAGAGAGAGGAATTATTTTTTGTGTAGCTGATATATGGT<br>CCCACGATTTTTATTATTTTTTGGGATAAAATTGCCATGTAAGCGATACGTGGATGCCACGTGGGACGAAGACATAGTCAAAGGAGCTACGTAGGCGTCTCATCGGCCAAAACCGCCCTTCATACCACCGAGGGACCTCGTTTGC<br>CCGGTTTTCGTAAGTTGGGGGATGGATCGTATTCGGTTTTGTAGTTAAGGGACGAAAATAGGACTGACCGACAAATAGAGAGACTAAGGGCCAGTTTGTTTTGTGACCAATGTTTTTGGTAAGGCCAACATGTGTTTGGATTGAA<br>GCCAAATTAAACTAATTTGGCTATTAAAATGGCCTATGTTATATAGGCATGTCAAAAACATACACAATTATTGTTAAGGTTGCAAAAAAAATTTGACAAGCCTAATTTAGGCCTGCCCTAAGTGACGATATGCATGGTCCCATGGCC<br>ATATGCTACCGTGTTCCGGCCCATGAACTACAGCTAAGGGCCTACATAGGTGATGCTGTCAGCCTGCCACAGGCACAGCCCAACGGCTAACCACTGTCGAGCCTCTGCACGAACACCGGACTGGCCCGGCACGCATAGCACCTGC<br>GCAAACAGAGGGAATTTTTTTTCCATTTTTCATTTTTTATTTTTTAATATTTACAGAAATATTGTACCGACGAAAATATTTACAGAAATAGACCCTACCGCCCTCTCTGAGAGTGGCAGGGTGACGCGAGGGACGGACAAGTTGAA<br>AAAAAAAACTAGAAAATATATTTTTGGTAGGGCGAAAATTGCATTTACTCCCTTGCAGCCTTTGTAGAGGGCGGCAAAGGACCTAAGTGCAAAATGCGCACACCTTGCAGCCCTCCACTTGGACGGCAGGGGCCATTGTGTAAAT<br>ATTTTGGATGGTATAATATTTCTATAAATATTAAAAAAATTAAAATTAAAAATGAAAAAAATTCCTCAAACAGGCTCGTACAAGGCTCCTTCCATCTACCATCTCTTTCTATACGTTCCAGGTGGCGAAACGTAAGAGAAAAAGG<br>AAAAAAAAAAAAGCAAAAGCCGTCTCCAAGAAGGCACAGACGCTATACATAACTCCAGCGTTTTCCACTTGGCGCGGGAACCCGCTCGAGCGGTCGAGCCGGACGGACCAACCTCCCAAAGGAAACCAAAAGAAGATCTCACG<br>TCGTCGCACCCAACCCACCAACCACCCGCCGCACGAAACCTGCGAGCGAACCGAGAGAGATCACCATCCCCCCGCACCGTTCTGGAGACGTCCCGTGCGCGCGCCACAACACCCGAACCCTCCACAAGCCTTAAGCCCGGCCCC<br>GAGCCGAAACCGCCTCCGCCTATTCGCGCCACCCACATCACGCCCCTACTCGAACATTCGTGAACGCCGCGCCGCTCTGCTCCTATATAAGCAAATCCCCTCCCTTTGCCTTTCTCCCCCA
Os<br>Hs<br>p11<br>0-8	Os12<br>g057<br>60	GTAGATCAATTTTGTCCACATAAGATTGATATCTCTGGCATATGGCCCAATGGTCAGTGACCCTTCATTCTCATGATCTCTGGGGAAGCAGTAAGAAAACGCGGTATAATCTGTCTGCTGACATACCTGGTTGAAGAATGAGTAAC<br>TTTTAGTCCCAAAACCAATTATCCCAGCATTGACTCCATTGTAAAGATTATCTTCTCCACAGCCAAAGATGAAATTATCAATGCTTCTGTTGCTGGCAAGAGTGAGCCTGTCTTTCCCTAAGTACCCAACTGAATACTCTCCTGACC<br>CATATCTTAAGCTGTAGATACAAGTATCATCCTCTTCTACACACCCATACTCAACAGCAAGATCCATGTGCATGCCATTGCAAGCCTCAGTTGAGCACCCAACTTTACTGTAAGTTGAAGAATTGTAAGGGTTGAATATTTGTCCA<br>GCTTTTGCAGCTTGGTCATAGCATTTTATCTGACAATTTTTACACTGAACCCAGGAGAGAGTTGAGCCGGTGTCAATGGTCACAAGATTGAAGACAGGTGGTGTGCCAAGGCTGATGCCCATGAAATACTTGTTCTTGCGCATGCT<br>ATCATCGCCTATGACGGTGGAGGAGTCTGCAGGTATGTTTGCTGGCTGGATCATTGTAGATATGAAGGAATTGTTTATGTGGAACACAGAGAATGTAAAGGCTCCATTCGTGTTTAAACCAATACCTGTGTTGTTCAGGAGAAAA<br>ACAGTGTTTCATGAATCAAGAACAAGAACACATAAGTATGATGTGTTAAGAATGTTTGGCTTACCAATATTAATGCAAGGACTACAGGTAAACTACCATGACATAGTTGCAAAAGAAGTACTTAATCTTGGTTTGTTGATGGTTTT<br>TATATGTTGCCTCGTGTCATAAGTGTGTTTATTTTTCATGATTCCATATTTAATACGAAAAATCCATACCTAACCGAATTGCATTCTGATGGAATAGAATACCCATGCAAATTGCAAAATAAAACTACCTTGCACATCAGGTTTACA<br>AGATCACTTCTGACAGCAAAAGAAAAAAAATTAGTATTTCACCTGCCAAGCTTTTTTGGGGGAAAAAAAAGATGGCACTTTGGTATGGCATTCCAACGTAAAGCAGAGCTTTCTTAGCTTCGATTGTGAGCATAATTAACAGATT<br>AGATGAAAAAAAAGGAATGACGAGCGCATTTTCTAGAAGCCATAGCATCATATAGCAGTAAAATTTGAAACTTCATTTCTGAGTTGATTTTTTGCTTTACTCATCATAGTCAAGTTTTCAGCATTGGCTTTTAAACTGCTAATAAT<br>ATGGATATAGAAGTTCTGCCCATAGCTTAAACGATCATAGCATAATACAGTTGAAATTAAAAAATTAAAAGTCATTAACCACACATTTGACTGAATTTTGTATAACATACAAATCGCGGCCACCCATGTATACGTTTTTCATGTAA<br>TCGGGGGAAAAAAAACGGAAACATGATTACCGTAGGTTGATGAGGAGGCTGGACTAGTAATGCTCATGCACAGTCGAATTGTGCACCATAGCGAAATCACCATGACAGCCAAGAACAGCTCCATCAACTATGTCGATCTTCACA<br>AAGTCTAAGCTAGGTGCTGAGTAATAAAATGAAATGTCTCTAATTCTCCTATTTATTATATGGGTTTTAATTAACACATGCTGCCTGACAATATGCATGCTCCAACTACCAACAATAACAGATATTTCATCACGATGAATATGCTAT<br>TTCTTTTTTTTTTCTCCCCTAGAAATCCACTTAATTTCTCTACTGTTTGGCTGACATGTGGGCCCAACAGGCAAAACCCTTCCTGTTCTATCTCATTCTCATCGTCCTGGATTAATTAATCCAACCCGAAAATGAATCGGATTAGGATC<br>CAACGAACCCGAAGAAGAATCGGATTAGGATTCAACGAGGCAGATTTAAAGCCAAAGCGATCGAATTTCTCTACAGAGATTGCTCGATCGATCGATCGATG
Ppc<br>Hs<br>p70<br>-2	Phpat<br>.003<br>G105<br>300	TAATATAAACATTATCTTAGAAACAAATATTGAAGCCATTTATTTGCAATCGATTTCAAATGACAAGAAATAAGAGTGACTATAGATCTCCAAGTTGGCCTTTAAATGGAGGCAAATTTATATTCTACATACATATACATAAGTAT<br>TCTTTGTGCTTTGTAGAGGAATAGTGAATGGAAAATAGAAATCAAGGAATAAAGAACAAGAAATAAGAATAACTAGGTAAAGATTTCTTTTGGCTATACACTTGCACATATGCTTTGATATGTGGATTAGATCTTGAATCAAGTG<br>GTATTTTGTTGATTTCGACAATCTAATACAATTAAACGCCACCAATATGAATTTTACTCATTTTGGAGTACCTTCACACGTCAATCTTTGGGTGACTCTCTCACCAAAAAAATGTTTTCAAAGAATTTTTGGAGGAAAAAAAATTCTTT<br>TCTCTTCCAAAAAGTTATTGTACTCTTAGCTCCTTTGTAACATTTTGTGAATATTTCTTTTTGGTCTATTCTCTTCTTTTGATGTTTTTTAATAAAGTTATATTTTTTAGTATTTTTAGTATGTATGTATATATATATATATATATATATA<br>TTTTCATAAGATACATGTGTGATAAATTAAATGTTGTAACAAACTTGTTAAACTTTTGGATGTCATATATTGTTGTTTACATAACATTCAAAAAATCTATTATCATTTTTCTCATTCTATCATTGATACAAAAGTTATCATATTTGAC<br>AAATATTTTAATTTAAGAATGTTTTAATTTAAGGATGTGCTAAGGAGGCTATTGCTAGATATCTATTCTTTATATCCTCAAATGTTTTGACCTCTTTTGTTATTAGAGCAATGTAAGATGTAGCTTTTACGTGCAAAACATGTGTATT<br>TGTCTATTATTCACTAAACTACAATAAAGTACTTGATCATATTAGAATGATTCTAATTACAAATATTATAAAATGTGATCCTTCTATAATAATCTAAATGTGATGATGTGGGTTATATTGATTTTAGACTTGACATCAAACATTCAT<br>TAATAAAACTGAAATTATAAAACTAACACTAGTCTCTAAGATTATCACATGTAATTGGAACTTGACCAACTCCCATCAGCTACCAAGCTTGATGTTGGATTATGAAAGGGTGAATAAATCACAATGAACTACTAATGGATGAATG<br>CATAAATGAGCTAAAAGAGTCCTTTTCCATGGGGGGAATAAAAATGAGAGATACAAAAGAAGTAATAGATTGTGTGGAAAGGGAAGCAGCCCCTTCTTCTTAAAAGAAGAATGTTACCATGATGGCTTGGTCGGGGATAGAGGT<br>GGGGGGGGGGAGGAGGAAGGAGCAGAAAGAAAGGTTGGATGCGGTGGAAGGGGGAGTGGGGGGGTTCCGACACATGGCGATGCACATGGGGATCGGGGGGCGTTGAGGTGGTCGGCTGCGACGCCCCCAAGGCCGGATTTGG<br>ACCCGCTTGACGCACCGACTGGCAGTGCGAACCGGATTTTGGCCATTGTGGCCTGTTTTCGTCTATTCTGGACACTAGACCCGGACTTCACCCCTTCCTTCCTTGTCTTGATTTTCTCAAGGTACGCCCGGGCCTGGTCAGAGCTTA<br>GGGACGGACGGAGGCCAGAAGAAGAAGAGTCTGGAAGCCCCTGGAACTCCCCCAGCGCTCGCGGGTGCCCGGTGGAGGGCCGGGTCTGGGGGGTGAGTCATTGGGTCCGGAACCCTCTAGAAGTTGCTGGAACGCGCTCGAAC<br>CCGGCGAACCTTCAGCTGCGAGCAGCCAACTGTTGATTCGGCCGATATCGTCAGCCTAGAAAACTCAAGCCTCGCTGCTCGCTCTCGCCAATTGATACTTAGATTTGTCTCAGCATCGCGGCTCTGAACTTGCGAGCGTCCGTCTC<br>CGTCTCTCTAGCTACCAGTGCCCTTTAGGTGTTAGATTTGGTTTCCTTTTTCTAGCGTGTTGCTGTTGCGTTCGAGGGGAGTTTTCTAGTGAGCGTCGCCA
Pp<br>mt	Phpat<br>.001	AAACAAAACAATGGGAAAATGAAAGCACCTGCGCCAGCCCCTGCCGCGCTGTTGAAAATGATGGACCGCTGCTCCTCTGGCATGGCGTCCAACAGCAATTTCCTCCCTCGGCAGCTCCTACGCACGATCTACGAGACACCTGTCC<br>AACATATCAGTCACCGCAGCAGGGCATAAGCCACGCTCAGCACAGGAGCGTTGAAGCCGTCGGTGCGACCTCTTCGCAGCACGGCAAAATGCATCCCACCACAACACATATCACCCCGGATTCTTCACAGAGAACCCCTAGTTTG

Hsp70-2	G091000	CATCGACACATTTCAACAAACGCCGCGACTCAATTAGTCCCTGTAGCTCCGCTAGAACCGAGCCAGGCGAGAGGAGGTGAGACGATGCTACTGTTTCTTCCAATGCTTCCAGGCATTCACGTAGCTCGCAGAAGTCCGACTCTAC<br>AAATACCCACTCGCTCTATGCTACTCCTGCTTATCGTCTTGCTGGAAAACTACGGAAGTTCGACGAACCCACATAAATCTTCTGAATCTTGCTCCGTCGCAATACAGTTCGAGGTAGACGAAAACATTCGGTTGGTGTGGAGGGTT<br>AACGATGCCGACACATGTTGAGGGAAGAACAGGGTGTAGGAACAAGGGAAGTGAGAACAGGGCGGATTGGAATCTCCGCCACGTAGGACTTGAATATGACCTACGTCGAAAAGCAATTGATCTACGTGGACCCTCCCCGCCGT<br>GCAGAATCAGAATCAGAGTGGACGTTCTGTCCGGGATTCAGTCATCATGAGATTCAGCGCCGAGAAGTTTACTTTGTGTCAGATTTAAAGATTGAATAAATGAGAATGCAAATAAAAATGTGTATATATGGATCTACGTACATTT<br>AAAAGATTGCTATTGCCACTACAATAATTCTCATCCATTTCAAATTACTTATTTTAAACATTTAAATCCTTACTTAAATACTTAGAGGCGCCATAGTAGAGACTAATTACCATTCAAGTAAATACCCAAGCTCAAGATTAAGACAT<br>ATATAAAGTGCCAAAGTTTAGACCAAGAATTATTTAAGAGGTTGTTTAGAATTTGGATAAGGGGATACTTCTAATGTTGTCTTTTTTTTTTTTTTTTTTAATATGATTCTCTAAATTTTTGTGATAATTCACAACGGAATATCTTTAC<br>AGCTGACCTAAACTAAACAGTGAACACCAACATTGTATTGTAATTCTAATTAAAAAGTAATGATGTTTCTTGGGAAATTATAAATATCCTTTTCGAAAATTGATACAGTGCCATTTTTTTACATAATAAAAATCAACCCACCCATC<br>TTCATTTTTCCAGAATCCAACTATATTTTCATTTGATTAAGACCAATACGGATAGAGTAGAGAATTCTTGATGATGGTAGAGATGGGTGGGTCAATTTACTAAAAACTTGGGCAGGTCCACGATTTCCTTCTCATATCGTATAGTAT<br>TGTAGGTCAGATGCACATGAACTGATGTCCCTACTCCAAAAGTTGTTCAGGGGAATTCAATGGAAATTACAGCAAAATTGATCCAGATATTGAAATCGCTAATCATCAACCCACAGGCATGGAGGTGCAGGCAGTGTCGGCCACA<br>TCCAAAACGACACCCACAAGGACATCCGTGGGGGAAACGCAAGGCCCGCGGGGTTGCAGGGGATGCGAACCCACACAATAATTACAGAAGGTGAAAGAGGGAGGGAAAGACGATGCGCGGGGGTGGGCACTTCGTGCCCCGC<br>AAGACAACCCCCCACCCACCACCACCGCTCAATCGGACCGTGAATTCCCTCGCTCACATCCAGCGCAGCGCAAGCAAACAAACAAACAAACCTTGGTGGCGTCGAGCAGGGGAACACGAGACGAGAGTGGAACGTCGGGGTTCCCG<br>AGCGGGGCCCCGAAAGAGGAAGGTGCCATCCGCAGTCAGTTGGTCGGTCAGTCAGTCAGGCAGGAGTGAGTAGGAAGGAAGGACCAAGGCTCCTCCCACCGGCGCTCCCCTTC
PpcpHsp70-1	Phpat.026G035400	CATATATACACATAAGTATATAGATTTGTAATAAATAATAAAAAATGGTAATACAATCTGTACACTATATTTCATTTGAATTATGTATTTTATAGGATTGAGGCCCTAATTTTAACTTAATTTTTTTTAAAAAAATCTTAATTAGC<br>ATAAGGGCTTTTAAAAAGTTGCCAAATGAATTTATACAGATAGGAGTACATGTCATTGCTTACTTAGAAAAAATCCTCAAAAAAAGTATAGAAAATTACAAGAAAAATGTATAATATTTATGTGTAATTATGTGTAATTTATATA<br>ATTTTCTTTTCTACAAAATATATTCATGTATAATTATTTTGTGCTTGATGGATCTACAAAGTCTGGTTTTAAGTTTTAATGAGAATTTAATGAAATTTTTTATAAGAAATTGAATCTTCTCCTCTATCTTTCCTATCAAGTATGCTATT<br>ACAATATGTTTTAAAAGAAAGTTTATTTTTATGCAATTTACTTCATTCCGTACATTTACTTCTTTTTTACATTGTTCTATCTATGCAATTTGTGTGGCAAAGCTCATCCCATAATTTTATCTTTAATAAGTTTCTATATTATATATATAAT<br>AATATAATAATTAGAATTCTTTCAATTCATTGTATCAAAATCTAAATATTCTTCTTTCTTAGCTAGTTTTATTAATATAATTTCAATCTGTACATGTTTTAGTTGATGCCTCTTTAATATATCTTTAATTAACCTCTTGCAGTTAATTTT<br>ATAGAGATTAAAATATAATATAGATCTATTGGAATCCATAAATTGATATCTATTTTTAATATATTAGAATAAATTAACTAATACCATTATTTTTGATGTTTAATTTTTTTCAATATCATGTCTTTTTCTAATTTTCTATTTACATTCTA<br>GTGTTTTTTTTGTAAAAAAAATCTTGTACTATATATATATATATAATTGTTTTTAATATGTTTTTCATATTAACATCATAACAACCAAAAACAATTCTACATTCAAATTCTATTATCCAAAAAAATTACATAGTGTAATTTTATTTGTAA<br>ATTTTTTGTCAAAATTAACTCTATTTAACAATCTAAAATAGACCACTTTTCCTTTGATGCTTTCTAAAATAAAAATATTTATAATCTCACAATGTTGTACATACATTATCTAAGGACATTTATAAATCTTACTAAGATTTATGCTATT<br>TTAATTTATAATGTGATCCTTATTGATTTATTTTATTTTATTTTATTTTTGTAATTCTTATTTGAGTTAGAATGTAGTAAATGTGTTGGTTATATTTACTTTGATGTTTTTACATCAAAATTGAATATAATTTAGATTATGTTATATTAA<br>ATTAAAAATAAAATATTTTTGTAAAAACTTGAGTATATATTTATAGAACATATAATAGATATTTTCATATATATAATGTGTTGATATATAAAAAAAGAATTATAGAACTTACTTTGTGTTACCACATGTGTCAAAAAGAGAGATTG<br>AACCCAATGAATCAATTATATCAATTCTATGTGGTTGCATGTAACAGTAATTGTCCTTCCACCAGTATCAACCTAATTTCACAAATTCCCAAACACTGAAAGTGAAAAGAAAAGAAAATACAAACAAACGAAAGATAAATAAAA<br>GGGTATTTCAATAGATGTAGTAGGTACGACAATTTTTACTTCATTACAAGGTTTGAGCATTGACCAAGTTAAATTCTGAGATCCAATAATGTATACTTTGATGTACCTTCTCCACTTCCCATAACTTATAATATTCTCATAAATATG<br>CAAATAACTCACCTTCCTTGCGTTCACAAGTTTTTGGCACTTCATCCACCAGAATAAATATTTATTATTACCTTGAATGAGTTGCAACACTCGACGCTATAGGGAATGCTACGTGGGCAGCTCTGGTGCCGCTTTTAATTTGAATCT<br>TATTTTGTTTTTAAATGTTTTTTTTTCCGCTTCGTCTTGGTCTTCTAGAAATGAGTCGACGTTTCCGGCGGAGGGCCCTGC
PpcpHsp70-2	Phpat.004G105700	TTCATCGTAAATGGATGTGTATTGAAGACTGGGCTGATGACTGAAAGTTAATTGCTAATAGCCACGTGTACATTTAGGAATTGATCACGACGAAGGGTATATTTACGCACACATGGCGAGATTTTTTTAATTCAATGCCGCACCG<br>CTTCATCTGAACAAGACAAACTTTATATATGTCATGAATGCTTCCATCACCTTCATAAAAGAGCGTTTTTCTTGATGCCAATGACCTGATTAGACAATAATACATTTGAAGCAATTTCAATCAGATCTTCTGCACTGGTTGCCTCGA<br>TATCATTAGCTCTTGGGTATTCTCCATAGAATTACGGTTTACAAATCTGAAGCAACAGAAGTAAGTGCCTCTGGAATCAAAATAAAAATCATAAGAATAAGAAAAACAGTAAGAGATTCGCTTATTCGCAGATTACTGCTATGTC<br>AGAAACGCTCTGCCGGGTACGATCGACGAGGGTACAGCTGGCTGTGAAGCTGAAAATTTCCGGCCCTCCAGCTTTTATTCTTGGCTTCTGCTCTAACCAATACAATGCTTAAATGCTCTGGAGTAGGTTAACTACAAAGAAAATGC<br>TGTTTCACCCTCACGTGTTGTCACCCACGTCTGGCGGGCACCATAGAAATGCGGGACACTAACTCCCTCCTCCCTCAATCCTCCTCATTTAAGGTCACTCTCATCCAGGACTGCCGTTTAAGTATGATTTGCAGGTTACCTGTACTC<br>TGGTGGTTTCTGATATCCTCGATTAAAGATGTTTCATCCTCCAAATAAATAAATAAATAAAATAATAAAAAACTACCACTCCAGAGATTGAATATTTCCGATGGGAGAAAGGGTACCAACATACCATTTCAGTAAATGAAAATTG<br>AGCTTCAGGATGTTCTGGTTGGAATGTTGTTTCCTCTTCTAACTGTAGGCTTAGCTGTGTGGTTCTAGTCTGCCGCCATAATGTTGTTCTCACATGACTCTTATATTGTACTTTTCGTTACCGTTAACCTTTTTGAATCTTCCGTGTGT<br>GATGAGGAAGGCATACTCTCTGAATTCCCCCTCACTTCGATCTGCCAAACAACTGGAGTCTCCTCTTTGAAAGTTAAGTCTAGCTTTTCCTCCTTACATTCACGCCTTCAGACAGTACCAGACCGTATCCACAATACTAAGAACCTG<br>TTTCGGAACCCGAGAAATTTCTACATAAGCGAGGACGCTGCACTAAAAAGAAACCATTCATGTGGACGGATCTTACCAACTTCTGCAGTTTAACACGAGGCATGTTTTAACAAACTCAGTAGAAAGCACCGAGCGGACTGAGGC<br>CTGGAAACGAAACCAACCTTGTTCGTTTTCCTGTAAAATCTGACTACAGATGCAACAAAGATATCACGGATGATCCAGTCGAGAATTACTGTCTGACGCTCATCCCAGAATCGGCACACCGTAGTTTTGTGATATGTTGCACTACT<br>CTCCTACCCATCTGCGCACCAAGTGTTCCAGAAATTGTCTCGAACGCAACAATCTAAAAATAAGAGCGGTTCCAACGTAATATGGGAACTGTTGTGAAAGCTAAGTTTAATCGATAACAACGAGCAGTCCTAGCAAGGGAGGGTT<br>CAAGAAGACATTCAGAAATTACTAGTCTCTGTTGTTGTTATTGTTTACGAATCAAATATCATTTTAACCCACCACAACTGAGATTTACGGGAGTTTAGAGCTGAGATTTCCCAGAACCAATAGGAACCCCAACAACACAAAGTTGC<br>ATCCGCGACACAACCCATGGACTCGCAAACTGTAGCCACCTGAATGAGACATTGCTCTTCGGAGATACGGAAGCTTGAATCTCGTCGACACTGCGTATTCTCGATTATTTCGGAAATTTCTGGAAGGTCGTTCAATTCTAGAAAAT<br>GATTTCCACGTTGGAATGGATCCACCACAATCCTCCACTTGGCAGCAGCAAGAACTGGAGCTTTCTTCGTCCAATTCTTCGCTTGAAATCCATCTTTTAGC
	Drought	
AtcHsp70-16	AT1G11660.1	TTCTCAAGTTCATAACGTTATAGACTTATAGCTTTTTCATAACCGAGGAAATTAGAATTCTTTCATTACAATAAAAAGAAAAGTCAGCGATTTTTCCCCTGCTAGATCAGTAATATTCCTAAATTATGTCCATTAATTGAAATCATT<br>ATTCATTAATTATAAGAAATCTATCAAAAAATTAAGAACTCAACAAAACTATTGATTAATGGTTTTTCCGACATGGACACAAAGGTTAAAACCAAATTCCACATATGTATTTTTGGGCTGCAAACTCGTAAATTAAAATGCTAAAG<br>TAGTAAATGTTATCTTATACATGTAAGTACTATATATATATATGACCTCATTTTTGCAAACAAATAAATTAAGATATTTGGACGGCAAGCTCTTAAATTTAAATGTTTACAGTCTAAGATGTTATCTTACAAATATATAAACTATATGA<br>CCTCATTTTGCATAATTAAATATGAACAATGATTTTTATATACAGGCATTAACTATAAGATTGTTAGCTGGAAAATTCATACTATCTTATATATTATCTTAATGATTTGACTTATAATTAAGTTTAATTGTCCTCTCTTTTTTTTTGGC<br>CAAAAAGTTTAGTTTCTAATAAGAAATTACATAACTTGAGAAAAAGAAAATAAGAAACACGTATACAAGCTACAAGTTAGTGCACCAGCACTGCTCAATGCAATGACTACAAATACTTGGTCATTTCAACTCCTCTCCTCATCTC<br>TCTCTGATCACACTACCTTCTCTTCCAAAAGTCGTCGCCGGGAAAACACAAAAAGACGAAAATGACCCTATTCAAGAGGCCATGTCTTTACTCAAAGATGGACAAGGAAGATCCAGAAGAGGTACTTAGCCGACGAGCCAAGTT<br>CTTGATTTACAAAACACTTCAAGAAGCCGACTTGATTTCTCGGCGTGATCCTCATTCGTCGTTTATACGGCTGAAGCTTTATCTGTTGAAGGTCAAGATCGGAAAGAGGTTGGCTAAGCTACGCCGGAGTGTAGTATCCGCCGTTA<br>GATTTGGCGGAATTCGAAAGCATTCTCATAATGGCGTGAGAGCGTTGAAGAAGATGTTCCAAGGTGGTGCAACAACTGGACTACCTAGGCCTATTTTCTCCCTTGAAGTTTGAAATTTCATTTATTTATATTTCTTGTTTTGTGTAA<br>TTTTTTTTTCTTTTGTTTCCGGAGTTTCTTTTGATCTCTATTACAAAAGTTTATCAAGAAATGTAATATGAAGAGATCATGAGTTTTGTTTCTTTCAAAAAGGTAAAAAAAAAATCTTGAAATAATCATTTAACTATATGATGATCTAT<br>TTTTATTTTGTCAAATTTTAATTATATGATGATGCTAAAATATATATAAGACACACAGATTAGTGTAAATGTGAGTTATGTTTACTTAATTTTTGTACCTAAACTTTTTTTTTTTTTTTTTTTTGCTAAAGAACCTAAACTTTTCAAAC<br>GCTCATCAAAGTGGTTAGAGATCAAATTTAAGTTTGATGTAAATGTGAGTTACGTTTACGTAATTTTTGTACCTAAACTTTTTTTTTTTGCTTATGAACCTAAACTTATTATTCAAACGCTCATCAAAATGGTTAGAGATCAAATTTG<br>AGTTTGGATATTTTGGATAGTCATAAAAAGCATGGTTGGATAGCTTTTTTCAAATTTCAAATTAGTACCACATATATGGATACAAATCAAGTAATTCATGCATGTTAGCAATATGTAAGTTCAAACGACCTACCTTTTAAAGCGAA<br>ATAAAATCTCAAGTCAAAAAGTTATTGAATCCGAAAATCCGATCCTACTCGATTGAAAGTTTGAAACATGTGGGGGCATTCGTGCGACATCGTGAATCAATCTTGCGCTAGGTTGTCCAAAAATATCTCTGGGATATAATATCAAT<br>TAAAAGGAATTAATAAGGACTTAAATGCAAATACATTTTTCTTTTCGTTAGCCTTTCCCTTGTCTCTTATTTTTCTAATTTCTAGCCCTTTC
AtcHsp70-5	AT1G16030.1	ATTGTTGTCTCTCCATCTAGCAATCAAGTCAAAACAAAATGACACTCATTCTGCCGATCCAGAGACGATCAATCTATTCTAGGCTAGCTTCAAATTCTTGTGTTCGCTGAGAAGAGTCAACAAACTCACCATTTGTCCACCAATGT<br>AGCCTCCTATAGCTCCATAACACACACATATAGGCCATTCCTGTCGAGAAAAATAATATAAGGATTAGTTCATTCTTGCAATAACTGCGAAAAAAGGGAGCAATAAAGTTCAGAAACAAGCCCTTGACTGTATGTTGAGAAGAA<br>ATAAACATTACCTGCCATGGTCTTTCCCAGTCAAGTGGCATAGGCCAAGCTCCAAACCATCCTCCAATAATTGCTCCATATGCTGGAACTAAGAGCATATGTTCTATAATTCCGATTGGTCTAAAAGAAAAAAAAAAGATGGATT<br>AACAACAACATAAAGTGTGTGAATTATCATACTAGGTAGCATAAGTGACTTACTTTAGTGAAGCAAAGATACGATGCCAATCTATCCATGATGCACCTAAAACTGCAGTTGCTGGTACAACCTATGAAAATCAAAATCATCAGAC<br>ACCATCAAAAGGTTTGTTTCAAACTGCAGAAATACACATATGGCATGGTATTAACATCTTTTAAGTCATGGACACTGCAGCTTACCGTGAAAACAGACATTAGAAAGGACCAGTGAATTGTTTTTGATAGAGATCTGCATAAGAT<br>GAGAAGGTAATACTCATGTCAGCTTTTGAAAATCACACAATCTCCATGCAGAAGTGAGACCACAACCAGCATTAAGGAGCCTCATTTTTAATCTGGACAACTGAAAAGTCCAGATTTACAATCAACATTTCCACAATTAGAAAGA<br>AATAAAAAGATATAGAGAAAGAAGATCTTAGTGAAACATACTGCATTCCAATAGGTGCACCCAAAGAAACAGCTCCCAAAGCATTTATAAGAGCACCTATTAAACAAATCAAAACACAGGTATAGATATAGTAGACACAATCA<br>AGGGATACTGAATCAGCAAAAGCACAAGATGAAAGCATAAGAAGCTCAGACTCACTCACATCTTCAGGTTTAGCTTAACATCTAGAATTTCCAGGTGAACTCATTGAGCAATCAACTTCTTTGAAAAATTAGTGAGCTATAATAA<br>GCTGAAAAACAGAAATACTAACCGGCAATTAGTCCGACTAAGCTTCGGCCAACAGCTCTAAAGTACTGGAAATCAATGAGAAAATCATGAATACAGAGTCAAAACACACAAGACCAAAATAATACTATATGAAAAGAAACGAT<br>TTTTTTGAGCATTAAGAGTGTAAAGTGAGGATACTAGCTTGTTTTGTCAATTTCATAAAAGGGAAATGAGACAAGCACAAAGCAATCGAGTTAAAACGAGAAATTCAGTTTCTTTAATTCTCACAGAGAACCTCAGAGATGAACT<br>ATACTCACCGAGCATTTCTCTGGGTTTCGTCGGAACAAGCTGTAGATGATTACCACGATCGGGAACTCAATAATCTGAATATCAACATCAAAACAAAAAGGCTAAAATTAACTGAAAAATATCCACTAGCAACCAGGTTATGAAA<br>GAAAGTTTTAGTACCCATAGGAGACGCAGAGTGAGAGTTGGATCAGAAATGAGATCGACAGAGTATTTGTTACGGACCACGTGAAATCCGAAGATCAGAAATAACCCAGTAATCACATAAACAGCAAAAGCCCCCCAAGTTGAT<br>ATCGTGATACTAACGGAGATTTCTGGATTCTTCTTCTCCTTCGCCTCTTTCATGGCTTTTCCTTTCTCGTCTTCGAAATCACAGAACAAGTGAAGAAAGAAGACGTAAACAAAATATTGAAAATCCTCCAGAACTTACACTGGGCCT<br>TTTATTCTATATACGGGCCTACAAGTTTATACCATATGGGCTTTAATAGGCCCATTTAATTATCAAGCGGTCGCCGGAGATAAAATATATCCCGGTCGGTGAATCCAGAACTCTCTTG
AtHsp70-BiP1	AT5G28540.1	TCCACACCAAGACGGAATAGAAATACTGAAGATATTGACTCTCCAATTCGTAGAATGGTATATATCTTAAATGATTTGAGTTTATTTTAAATTGTTATTTGTATTCTAATTTTTTTAATTATGATGTTTCATTTAATTATTCAAGTAT<br>CATTTAATTCACATAACTTTAGTTTAATATTTTAAAAATATTTAGATTAAATTATTTTTTGAATTTATTTACCATTTTAGTGTTATTTAATTGATTGTAATTAATCATCGTAGAGAAAACATGATCAAAGATATATACTTGAAGAAGA<br>AAGAACGTTGATTGAATTATATGATGAAACACTAGTTTTAAACAATTACATGTTTAAAGATCCGGGACAACCGGGACGAGCATATATTGTTCAAAAAATTCAATGAAAACATCAAGTGAACAGATTTAAGATGCAAATTCGATGA<br>GTGGAAGAAACAATACAAAAGATGGATCCTTTTACATGGTAAGACTGGTATTAGGGTAGATACCATTAGTGGAAGAATTGAAGCTTCAAATGAGTGGTGTAATGAACGTATACATGTAAATTAATCTTCTTAAGTATTAATATGG<br>TTTAACTATTTGTAATTTTTTTTATTATGCGGTTACAATTTTTAATATTATATATTTTTGCAAGAAAACGACAGAGTGGGACGTTTTCAACGAGAACCTTCTAAGTTTTGGGAAATCTGAAACGTCCCATCCCGAATCGAGGATGTTAC<br>AAAATCATACGTATATGTTTTGCAAATCTTGATGTGGGTTCTCAACGTCAACATTCAGTGCGACAAAGAAGAGAAGAACTGATAAGAGAGAATGAAAGTTGTTTAGAGGATAGAAAGATGCATATGATGAAGAAAATGCATATA<br>GAAAACGAGCAAAATGACTCAAATGAACAAGAGGAGGTTGAGAGAGAAGATAGACAATATATGGCAAATATCTAAGAAAGTAGTGTCAATATGTTATTTTGATTTATAATTTTTAAAATATTGTTGTAATAGTTATTAAAATGAT<br>ATGTTATTAATTATTAAAATTGTTAAAATTTGTTTTGGTTTTCGGTACACACTTTTGCATACTACAGTACGTTTTTAACCATTCAAAGGATAAATTAAAGTTAGTACGCATGACTCCTTACCAAGATGTTAGTACGCATGTCTTAGG<br>ATAGAACATACCATTCAATCATTAAAGGCTTTAATGGGTCATTTCACTTGGGCTTCTAAAATTTAATATCGGTTCTATTTATTTTAGATTAGAACTAATTAAGTTAATCTCATTCTTCATAATATAATCTCATTTATGAAAGAATATT<br>TTTTTTAAATGATAACAAAATAAATATAAAACAAATACTTTTTAAAAAATCAATAGCAAAAATAGATAGTACGTTTCTCTTTGTTAACAATGATGCTTAGGGGTAGTAGACATTCGGGTACGCAGGTCGGGTTTGGATAAAATCC<br>GATCGGGTAAGGATCCTTTGGGTTGAGAAGTTTAGGATTCAATAGGATAAATCTAAAATCTAAATTTGTTTCCGGTTTGGGTCCAATCGGATTTGAGATTTTTTTACTATATTTTCAGATCTTTTTTTGGAACTAATACCCAAACT<br>ATTGAGTAAATATCTGGATTTTTGGGTAAAATTCGGAATACGGATTCGGGTTCGGATTCGATTTTTTTGGATAGAAAAATTTAGGATCCAATAAAATAATTTCTCAGCTTTTAAATTTAGATTTGTATCCAATTTTTCGGATGGTTT<br>GGATTATTCGGGTTAGGTTTTTCTTCGCAGCCCTAGTGATACTAATAGAAGAAGACTGGGCCCAACAAAAGCTCATTGTCTAGAACTAAGAAGAAGAACCGAAGTAACCAACGGCCACGATTACTCCAACACAAGACCAAATCT<br>GATTGGTTGACATTACAAATCGTCGTAAGATAATTGGTCCACGTCATCTCTGATGACATAGTTAAATTTCTTCGTCTTCCATAAAAAGCTTCTTCTTCAA
OscHsp70-4	Os03g16920	GAGAGGGCGTCGGCCTGGATCTGCACGAAAGGGAAGGGCGGGTGGGCGGCGAGGAGAATGACGGAGCAGGCGAGCGAGGCGGGAGGTGGCGGAGCGAACCGACGGTGGGCGGCAACCTCGGCAAGGTGGGGAGGAGGCGGA<br>GGGGAGCGCGGAGAGGTCGACGGTGACAGCCGGCGGGAAGAAGGCGAGCGTGCCGGAGATTGAGCAGAGGAACTCCGCCAGTGGGTCCGTCGTGAAAGACGACGATGACGAGGACATCGGCAGCTTGGAGGGCCGCGTCGGC<br>GGCGAGGATGGGCGCCACCACGGCCAACCTCTCGCCTGCGGCGCCGGCGTTGTTGTTGTCGTCCTCCGTCGCCTTGCCCCTCGTCTGTGCCGCGGCCGCCGCCTGCTGGAGCCGCGTGGAGCGCCGCCTTGCCCCTCGCCTGCTCC<br>GGCCGACGCCGCTCTGCCGGCCTGCTCTAGCTGCGGCCTGCCCCGCCCTGTCCCACCCCGCTCAGCCGGTCAGCCGCTCCGCCCCAGTCGTCGCCTGCTCCGGCTCGGAGAGAGAGGAATTATTTTTTGTGTAGCTGATATATGGT<br>CCCACGATTTTTATTATTTTTTGGGATAAAATTGCCATGTAAGCGATACGTGGATGCCACGTGGGACGAAGACATAGTCAAAGGAGCTACGTAGGCGTCTCATCGGCCAAAACCGCCCTTCATACCACCGAGGGACCTCGTTTGC<br>CCGGTTTTCGTAAGTTGGGGGATGGATCGTATTCGGTTTTGTAGTTAAGGGACGAAAATAGGACTGACCGACAAATAGAGAGACTAAGGGCCAGTTTGTTTTGTGACCAATGTTTTTGGTAAGGCCAACATGTGTTTGGATTGAA<br>GCCAAATTAAACTAATTTGGCTATTAAAATGGCCTATGTTATATAGGCATGTCAAAAACATACACAATTATTGTTAAGGTTGCAAAAAAAATTTGACAAGCCTAATTTAGGCCTGCCCTAAGTGACGATATGCATGGTCCCATGGCC<br>ATATGCTACCGTGTTCCGGCCCATGAACTACAGCTAAGGGCCTACATAGGTGATGCTGTCAGCCTGCCACAGGCACAGCCCAACGGCTAACCACTGTCGAGCCTCTGCACGAACACCGGACTGGCCCGGCACGCATAGCACCTGC<br>GCAAACAGAGGGAATTTTTTTCCATTTTTCATTTTTTATTTTTTAATATTTACAGAAATATTGTACCGACGAAAATATTTACAGAAATAGACCCTACCGCCCTCTCTGAGAGTGGCAGGGTGACGCGAGGGACGGACAAGTTGAA<br>AAAAAAAACTAGAAAATATATTTTGGTAGGGCGAAAATTGCATTTACTCCCTTGCAGCCTTTGTAGAGGGCGGCAAAGGACCTAAGTGCAAAATGCGCACACCTTGCAGCCCTCCACTTGGACGGCAGGGGCCATTGTGTAAAT<br>ATTTTGGATGGTATAATATTTCTATAAATATTAAAAAAATTAAAATTAAAAATGAAAAAAATTCCTCAAACAGGCTCGTACAAGGCTCCTTCCATCTACCATCTCTTTCTATACGTTCCAGGTGGCGAAACGTAAGAGAAAAAGG<br>AAAAAAAAAAAAGCAAAAGCCGTCTCCAAGAAGGCACAGACGCTATACATAACTCCAGCGTTTTCCACTTGGCGCGGGAACCCGCTCGAGCGGTCGAGCCGGACGGACCAACCTCCCAAAGGAAACCAAAAGAAGATCTCACG<br>TCGTCGCACCCAACCCACCAACCACCCGCCGCACGAAACCTGCGAGCGAACCGAGAGAGATCACCATCCCCCCGCACCGTTCTGGAGACGTCCCGTGCGCGCGCCACAACACCCGAACCCTCCACAAGCCTTAAGCCCGGCCCC<br>GAGCCGAAACCGCCTCCGCCTATTCGCGCCACCCACATCACGCCCCTACTCGAACATTCGTGAACGCCGCGCCGCTCTGCTCCTATATAAGCAAATCCCCTCCCTTTGCCTTTCTCCCCCA
OscHsp70-6	Os05g38530	AACGGTGCATGTGGTATCCCCTTGAACTATAAAAGGAGGACCTTACCCACCGAGAAAAGGGACGACTCTTGATAGCTTGAGCTCGAGAGAAAGCACAGCTAGGGTTTCCCAGGAGCTCAAGATCCCTTGTAAGAACTCCACCAT<br>AATTCCACACACAGGAGTAGGGTGTTACATCTCCCGGCAGCCCAAACCTGTATAATCCCTCGTCTCTCACACAGATTCGCAAGCGATCAAGTAGCCAGACCCTAAGCGGAGATACGCTCTTGATCATCGCGCCTTATCCCCCGGCC<br>GAACTCACAAAGGGGGGGTCTCACGACCTCCCGCTAGAGAGGATCACTCCTCGACACCTTGGTTTTGACAATAAGAAGCATGTTCACTTTATTACCATTTTCAACCCTATCAAATTTTGATTAGGTAACAAACTAAATATGATTTG<br>ATCAATCGCATACTATCAAAAATTGTTAGTTCCGGAATTTTCCTCTCATAAAAAGCTATCAAATTTTAGAAACACATCAAACTAAAATATAACAATATCAAAATTTAGCAGCGCTAAAATTTATCAAATTTGATATTGTCAAAATC<br>CAAGCATAAAACAGGCGAGAAAGCTACGGTGTACTGAACAATTCCATTGGACGAATCGATCTTGTGCATCTGTGCAGCTGTTTTCGCGGTACAGGGTGCAACAAAAGCCCATGACGGCCCACACCTGCCTCTCTCCGCTCCAAAC<br>ACCGAAACAAGGGGGTGGGTGCAATGGGCCGGCGCTCGAAGACCGCGAACTCTTTCCAACAGCCCAGCGCATTAGCCCCTCCTCCTACTCTCTCTACCTTCTTTTTAACATGCGACTTTCTTTCTGTGGACGACGGCATCAACGAC<br>GGGAGCAGGAGCGGGGGCTGAAGCACGGTGCGTGGGCTCCTGGAGTGGCGACGGCCTCTCCGGCGAGCTTCCTCTGGCGAACTCCCTCCGCTCCTCCTATGGCGAAATCCAAACAAGGGTCAGTTTCGACTCCAACCTTCTCCCA<br>CCACCACCTCCTGACCGTGCCACCACCCGGCCTTGTCGGCACTGAAAGGCGTCAACTTGTCAGCGCGGGCCTGCTCGGTCGGTCTCCTCCTCCCCTATTTCGTTTAGCTTTGCCCCCGCCACCAACACCGGCCCACGGCCCATGGC<br>CGACCCCGCGGCTTTGGCGCCGCCATCGCTATCTCGCCGCTGTCCTTTTTTCATGACCTTCGGTGCCATCCCTCTAAATTCGATGCACCTCCCTGGCTCTATCTCCCTTTACCTCCGAAATCCTAACCCTACCCATAATCTCTAGTGA<br>GTCTTGTCTTTATTTATGGCCTCTTTGAATCGCAGGATTGATAAAACGTAGGATTTTGATAGGAATGTAAGTGTAAAACACATGATTGTAAAATAGAGGAAAAACATAGGAATGGCCGTTTGATTGAACCGCAGAAAAAACACAG<br>GAATTAGATGAGAGAGATAGACTCAAAGTTACTAAGAGATTGAAGCTTTTGCTAAATTTCCTCCAAAATCTCTATAGGATTGGCCATTCCATAGAAATTTCAAAAGATTTAATAGGATTCAATCCTTTGTTTCAAAAAACTTCATA<br>GAAAATTTTTCTATAGAATTAAAATCCTCTAAAATTCCTATGTTTTTTCTCCAATTCAAAGGGGCCCTTAGGTTGGAATTTGGAAAGTGTTCGCGAGAAATCAAGCGGTCGCACGTTAGCGAATTAGGATTTCCGGAAACAAAGGA<br>CCGACTCCGCCTATCCATCGTCACGAGCACAGTGTAGAACCTCCCAGACCTCAAGAGACCGTTCAAAAAGCGCGCGCCCAAGCGGGGCCCACCAACGCGTCCCCACCGTGTCGCCTCCTGATTGGTTGTCCCCTCTTCCTTTCACG<br>CGAACCGGCACCCTCCCGACCCTTCCAGAACCCCCAATCCGACGGCCAGGATCGCCCGCGCGCGAACGTTCTAGACCCCCGCCACCTCCGCCACAAAACCTCTGC
OsHsp110-1	Os01g08560	AGAGAAAATAGTACTTCCTCCATCTACATTTGATAGTCGTATTTCATCTTAGCACACAGACCAAGGATAAGTAATTTTACTTATCATCCATTTAAACATGCTACTAGTCATTCCTCGTAAACAAGCGATTCATTAATATTTACATTT<br>CTCAATGCCCATGTAGCCAATCATGTGTGAAAGAATGGAGAGTCATGCGAGAAAGTCATTAAGATGATAGTTTGTTGAATTGAAATATGACTATCAAAAATAAATTTTTCAGATTTAGAAATATAACTATCAAAAGTAGATGGAG<br>GGAGTAATATTTTCGACCCGAGATAAATTTATAAATATTATTTTTTTTTATAAACTTGGTTAAACTTGAAGCAGCTTAACTTTGACCAAAATCAAAACAACTTATAACCTAAAACGAAGGTAGTATATACTTATATTTATATGTATAA<br>ACTTTGTAACTAAATATATACACATATGCTTATATACACATCTATACAAAATTATACATTATACATATAAACTTTGTATTCGTACATATAAAATTACACATTCTTTAGACATATAAAAAGTTTATACATATACGCACAAACTTTATG<br>CATATACAATTTGTACGTATACGTATGGAACGCGATGCGCTGATCGTGGATTAGCATTTTCGACTAGGTAGCTACATATAGCATTATCAAATACCCTCACAATAATAAATATCTAATTGTCCTTCTAAAGCAAGTATTTCCTACGTA<br>TGTTGAAAAAAAAAACTTATCTTTATATCTTGGGCTACTATGAAAACCGAACTATCTTTCATTAGATACGCAAATATATATTCATTTGATTATATGCATTATTATGAATAAGTGCGCGTAGCGTATAAGCACATGCAGTTATACTGTG<br>TTTTAAAAATACACAATCATATTTATAGTCAGGTAAACGTAAACGTACCATGTATTCATTTATGTACCGGGCCTGTTCATCTGCACTATAGCAGGCTACGACGCAGTCACCTCACACGGTATTTTGTGTAGCATAATTATGTGTTGG<br>ATTGGGATCAGGTGCAGTCCCAACTACAACTGCACACGTTAGGGACTACACAGGATCATGTCCATTTAAATTTTCAATGGAAGGTTCAGATTAAATGCTACAGTAGCATTGGTATAATAATTTTTGTTGGATTATCTAAATGAACA<br>GCGCTCTATTAAATTTGGGCCTTTGAATTTGCATCCTGCGGCCATAGTATAACTGTCTAAGTTGCAGTCATAGTAATAACTGCATAAGAATTAAGGGTCTGTTCACTTTGATGCCAAAATTTTGACAGGATTTCTTATATAGTTACC<br>AAAATTTGACAGCAAACTAAATGTAGCCACTTTTTTTTGTAACTTTACCAAAATTTGGTAAGGTTGTAAATGATATCAAAGTGAACCGGCTCCAAATCCGTATGTGTTGTAGCCGCTTTAACATGTTGCCGAACACGGCTGCTGCA<br>ACTTTATAGAGATATTATCCTTACTAAATAAAATACACCATCGATGTCCGAAGAAGAAAGGAAAAATCATATTGAACGATCAAACCATTGTCGCCGTATGTTGGTACACGGTATTAAACGTGCCAAAACCATCTTCCCCCTCCCC<br>ACAGACGTCAAACGTGGCGAACCACTTGACACCAACGTGTGCACGTCTGACTTCTTGCACTCCCTATACGTCTTGACCCCCCCACCCCCCAAACGCCCAACGGATCCTTCTGGAACCCACTCGCGCCGTCAAACCACCACCGCCTC<br>CGTCTCCTTCTCCGTTCCCGTCTCCGTCTCCCCCAGAGAGCTCTCGTCGCGCGCGCGCACTTTTCTCCTCTCCGACTGCGGGGCCCACCAGTCAGAAGAGAACTCTCCTGGCGCCTCTCTCTCCCCCAACCCCACCGGATACCTGAT<br>CCGGGCCGTCCGTTCGATCGATCCGACGGCGGAGACGTTGTCCTGGGCTCCCACGGCAGGAATTGGATGGTTCTAGAACTCACCCGAGTCCCGACC
OsHsp110-5	Os05g51360	GACAAATGAGGGGAGAAGCAATAGAGCTATAAATGAGTAGAGTGATGGCGGTTGATGGGACATCTAAAACGGTAAGCAAAATCCTAAATAGAATCTCAATGACGATTAAGAGGAGGGACGATGGGTAGGCTATTAAACAGCAC<br>AATCACGGTGGTTGGTGGGTTTTATAGAAAGTAAAAAATAAACCCCAATGATAAATATGTTCGATTTTTAAAATCTCAATGACAATAGAGAGAGGAGTCTGTGGGCCGGCAGTAGAGGAGTACAGTGGCAAAGCCGTTGACAGT<br>TTGACAGGACTTCTAGAAAATAAAAAGTTAAACCCAAACGATAATTATGCTCGATCTTTAAAACCTCGGTGATATAAAGAGAGGACGCAGCGAGCGGGCCGTAGAGGAATACAGTGATGGCGTTTGACAGAACTTTTAAAATTA<br>TAAAAACTAGCATGGTGGCGCGCGCAGATTACGCGGCTAGCATCATTGTATTTTCTTTCATATAAAAGCATATATGTTTTCTTAATGTATTGTTCAAATATATTAAAATGACAATATAATTTTAAATTTTGTACTAATTTTACGAAA<br>CTACTAATGTGTAATATTCATATTGTATTTTATATACGTGTTAGTTGTTAATTATTTTTAATATCAAATTTTAGTTATTTGTAACTTATATTCCTATATGGACTCTAGACTCGTCTTTCAATATTTCTTTTTCTAATTCCGAATTTTGGT<br>TATCTGTAAATTGTATTTCTACATAGACTATCTGTAAATTGTATTTCTATATAGACTCTAGGCTCTTCTTCTAATATTATTTATTTTTAATTCTGAATTTTTATTATTTCTAATTGTATTTCTATGTGGACTCTAAACTCCTCTTTCAAT<br>ATTCTTTAATTTTTAATTTCAAATTTTAGTTACTTCTAAATTGTATTCCTATATTTACTTTAGACTCTTCTTTCCTTGTTTTTTTTTAATTTCGAATTTTAGTTATCTGTAAATTGTATTTTTATACGAACTCTAAACTCTACTTTTAATTT<br>TATTATGTTTATTCCGAATTTTTGTTAGTTTCAAATTCCTATATGGACTCTATACTCTACTTCTAATATTCCTTATTTTTTAATTCCGAATTTCTATTATTTCTTAATTGTATTTCTATATGGACTCTATACTCTACTTCCAATATTCCTT

		ATTTTTTTAATTCCAAATTTCTATTATTTCTTAATAGTATTTCTTTATGGACTCTAGTGCACTCTGTCAATATTCCTTATTTTTAATTCTGAATTTTAGCTATTTTATATTGTATTCCTATATGGACTATGTTTTTTTTTCTCCTATTAAT<br>GTGGGAATTTCTAGACCGTGAGAGCGAACGTGGAGGCTCCTTTTTCTATTACTTTAATAAGATAATAGATGATAACCTACGAGACAATGAACTCTAAAAACTACAGAGTCAAATTTTTAAAAGGTTTTAAGGAGGATGAATAAAA<br>ACAATAGTAGATCGGCAAACAAATAAATAAAGAGGTGATAAAGAAACAAGGGGTAGCGATTGACGTGAGTTTTAAAAATATAAAATTAGAAACAATTCGGTCTTTCAAAGTCCTAAAACAACGAGTTAGCTATTTAACAAAACT<br>TAAGTAAAATCATATGTAAAAAATAGATAATTTTGTATGGGTACTAGCCATGCAATTGTGCGGATCACCCGGCTAGTTCGACGAATTCAAGCGTTTTTGGGTTGCAGCGTTTGGAAGGAGGCCCAAATTAAAGTGGTGATCGAA<br>ATTGTCAAGTGCTTGAGTAGATAGAGATTTTGTTTGGTTGGCACTACCGAAGCATGCATTGTTGGAGTGGGATGCCATAGAGCTGCGATCAAGATCCGACGGCTCCCAATGGCTCTGGTGACATGTCTCCTTGACACTTTCCAAAA<br>ACGGACAATTGGTCGGTTGATGTTGGGTTCCTTATTTGTACTACTCCAGAAGTATATAAAAAGCGGAAGTTCCCAATGCAACAAGAGG
PpcHsp70-2	Phpat.003G105300	TAATATAAACATTATCTTAGAAACAAATATTGAAGCCATTTATTTGCAATCGATTTCAAATGACAAGAAATAAGAGTGACTATAGATCTCCAAGTTGGCCTTTAAATGGAGGCAAATTTATATTCTACATACATATACATAAGTAT<br>TCTTTGTGCTTTGTAGAGGAATAGTGAATGGAAAATAGAAATCAAGGAATAAAGAACAAGAAATAAGAATAACTAGGTAAAGATTTCTTTTGGCTATACACTTGCACATATGCTTTGATATGTGGATTAGATCTTGAATCAAGTG<br>GTATTTTGTTGATTTCGACAATCTAATACAATTAAACGCCACCAATATGAATTTTACTCATTTTGGAGTACCTTCACACGTCAATCTTTGGGTGACTCTCTCACCAAAAAATGTTTTCAAAGAATTTTTGGAGGAAAAAAAATTCTTT<br>TCTCTTCCAAAAAGTTATTGTACTCTTAGCTCCTTTGTAACATTTTGTGAATATTTCTTTTTGGTCTATTCTCTTCTTTTGATGTTTTTTAATAAAGTTATATTTTTTAGTATTTTTAGTATGTATGTATATATATATATATATATATATA<br>TTTTCATAAGATACATGTGTGATAAATTAAATGTTGTAACAAACTTGTTAAACTTTTGGATGTCATATATTGTTGTTTACATAACATTCAAAAAATCTATTATCATTTTTCTCATTCTATCATTGATACAAAAGTTATCATATTTGAC<br>AAATATTTTAATTTAAGAATGTTTTAATTTAAGGATGTGCTAAGGAGGCTATTGCTAGATATCTATTCTTTATATCCTCAAATGTTTTGACCTCTTTTGTTATTAGAGCAATGTAAGATGTAGCTTTTACGTGCAAAACATGTGTATT<br>TGTCTATTATTCACTAAACTACAATAAAGTACTTGATCATATTAGAATGATTCTAATTACAAATATTATAAAATGTGATCCTTCTATAATAATCTAAATGTGATGATGTGGGTTATATTGATTTTAGACTTGACATCAAACATTCAT<br>TAATAAAACTGAAATTATAAAACTAACACTAGTCTCTAAGATTATCACATGTAATTGGAACTTGACCAACTCCCATCAGCTACCAAGCTTGATGTTGGATTATGAAAGGGTGAATAAATCACAATGAACTACTAATGGATGAATG<br>CATAAATGAGCTAAAAGAGTCCTTTTCCATGGGGGGAATAAAAATGAGAGATACAAAAGAAGTAATAGATTGTGTGGAAAGGGAAGCAGCCCCTTCTTCTTAAAAGAAGAATGTTACCATGATGGCTTGGTCGGGGATAGAGGT<br>GGGGGGGGGGAGGAGGAAGGAGCAGAAAGAAAGGTTGGATGCGGTGGAAGGGGGAGTGGGGGGGTTCCGACACATGGCGATGCACATGGGGATCGGGGGGCGTTGAGGTGGTCGGCTGCGACGCCCCCAAGGCCGGATTTGG<br>ACCCGCTTGACGCACCGACTGGCAGTGCGAACCGGATTTTGGCCATTGTGGCCTGTTTTCGTCTATTCTGGACACTAGACCCGGACTTCACCCCTTCCTTCCTTGTCTTGATTTTCTCAAGGTACGCCCGGGCCTGGTCAGAGCTTA<br>GGGACGGACGGAGGCCAGAAGAAGAAGAGTCTGGAAGCCCCTGGAACTCCCCCAGCGCTCGCGGGTGCCCGGTGGAGGGCCGGGTCTGGGGGGTGAGTCATTGGGTCCGGAACCCTCTAGAAGTTGCTGGAACGCGCTCGAAC<br>CCGGCGAACCTTCAGCTGCGAGCAGCCAACTGTTGATTCGGCCGATATCGTCAGCCTAGAAAACTCAAGCCTCGCTGCTCGCTCTCGCCAATTGATACTTAGATTTGTCTCAGCATCGCGGCTCTGAACTTGCGAGCGTCCGTCTC<br>CGTCTCTCTAGCTACCAGTGCCCTTTAGGTGTTAGATTTGGTTTCCTTTTTCTAGCGTGTTGCTGTTGCGTTCGAGGGGAGTTTTCTAGTGAGCGTCGCCA
PpcpHsp70-2	Phpat.004G105700	TTCATCGTAAATGGATGTGTATTGAAGACTGGGCTGATGACTGAAAGTTAATTGCTAATAGCCACGTGTACATTTAGGAATTGATCACGACGAAGGGTATATTTACGCACACATGGCGAGATTTTTTTTAATTCAATGCCGCACCG<br>CTTCATCTGAACAAGACAAACTTTATATATGTCATGAATGCTTCCATCACCTTCATAAAAGAGCGTTTTTCTTGATGCCAATGACCTGATTAGACAATAATACATTTGAAGCAATTTCAATCAGATCTTCTGCACTGGTTGCCTCGA<br>TATCATTAGCTCTTGGGTATTCTCCATAGAATTACGGTTTACAAATCTGAAGCAACAGAAGTAAGTGCCTCTGGAATCAAAATAAAAATCATAAGAATAAGAAAAACAGTAAGAGATTCGCTTATTCGCAGATTACTGCTATGTC<br>AGAAACGCTCTGCCGGGTACGATCGACGAGGGTACAGCTGGCTGTGAAGCTGAAAATTTCCGGCCCTCCAGCTTTTATTCTTGGCTTCTGCTCTAACCAATACAATGCTTAAATGCTCTGGAGTAGGTTAACTACAAAGAAAATGC<br>TGTTTCACCCTCACGTGTTGTCACCCACGTCTGGCGGGCACCATAGAAATGCGGGACACTAACTCCCTCCTCCCTCAATCCTCCTCATTTAAGGTCACTCTCATCCAGGACTGCCGTTTAAGTATGATTTGCAGGTTACCTGTACTC<br>TGGTGGTTTCTGATATCCTCGATTAAAGATGTTTCATCCTCCAAATAAATAAATAAATAAAATAATAAAAAACTACCACTCCAGAGATTGAATATTTCCGATGGGAGAAAGGGTACCAACATACCATTTCAGTAAATGAAAATTG<br>AGCTTCAGGATGTTCTGGTTGGAATGTTGTTTCCTCTTCTAACTGTAGGCTTAGCTGTGTGGTTCTAGTCTGCCGCCATAATGTTGTTCTCACATGACTCTTATATTGTACTTTTCGTTACCGTTAACCTTTTTGAATCTTCCGTGTGT<br>GATGAGGAAGGCATACTCTCTGAATTCCCCCTCACTTCGATCTGCCAAACAACTGGAGTCTCCTCTTTGAAAGTTAAGTCTAGCTTTTCCTCCTTACATTCACGCCTTCAGACAGTACCAGACCGTATCCACAATACTAAGAACCTG<br>TTTCGGAACCCGAGAAATTTCTACATAAGCGAGGACGCTGCACTAAAAAGAAACCATTCATGTGGACGGATCTTACCAACTTCTGCAGTTTAACACGAGGCATGTTTTAACAAACTCAGTAGAAAGCACCGAGCGGACTGAGGC<br>CTGGAAACGAAACCAACCTTGTTCGTTTTCCTGTAAAATCTGACTACAGATGCAACAAAGATATCACGGATGATCCAGTCGAGAATTACTGTCTGACGCTCATCCCAGAATCGGCACACCGTAGTTTTGTGATATGTTGCACTACT<br>CTCCTACCCATCTGCGCACCAAGTGTTCCAGAAATTGTCTCGAACGCAACAATCTAAAAATAAGAGCGGTTCCAACGTAATATGGGAACTGTTGTGAAAGCTAAGTTTAATCGATAACAACGAGCAGTCCTAGCAAGGGAGGGTT<br>CAAGAAGACATTCAGAAATTACTAGTCTCTGTTGTTGTTATTGTTTACGAATCAAATATCATTTTAACCCACCACAACTGAGATTTACGGGAGTTTAGAGCTGAGATTTCCCAGAACCAATAGGAACCCCAACAACACAAAGTTGC<br>ATCCGCGACACAACCCATGGACTCGCAAACTGTAGCCACCTGAATGAGACATTGCTCTTCGGAGATACGGAAGCTTGAATCTCGTCGACACTGCGTATTCTCGATTATTTCGGAAATTTCTGGAAGGTCGTTCAATTCTAGAAAAT<br>GATTTCCACGTTGGAATGGATCCACCACAATCCTCCACTTGGCAGCAGCAAGAACTGGAGCTTTCTTCGTCCAATTCTTCGCTTGAAATCCATCTTTTAGC

Table S6 *Cis*-element of *Hsp70* gene families under different cellular locations and treatments.

Cellular localization	Number	Sequence	Name	E-value
Cytoplasm	C1-1	CTCCCTCTTC	NODCON2GM	7.30E-03
	C2-1	GGGGCGGCGG	GCCCORE	3.50E-35
	C2-2	AAAAAAAA	None	2.30E-08
	C2-3	AGAGAGAGAG	CTRMCAMV35S	7.90E-06
	C2-4	CCCCGCCGCC	GCCCORE	1.70E-02
	C3-1	TTTTTTTTTT	MARTBOX	1.10E-10
	C3-2	GCCAGCCGCC	AGCBOXNPGLB	7.30E-04
	C3-3	GGCAGAGAAG	None	6.40E-06
Mitochondrion	Mt-1	GGCCGGCGGG		1.10E-10
	Mt-2	TTTTTTTTTT	MARTBOX	1.60E-07
Chloroplast	Cp-1	GGTGGCGGGG	SORLIP1AT	2.70E-10
	Cp-2	TTTTTTTTTT	MARTBOX	5.80E-07
ER（Bi）	Bi-1	CGGCCGCGGC	CGCGBOXAT	6.60E-20
	Bi-2	AAAAAAAAAA	MARTBOX	1.90E-16
	Bi-3	TGGTCCACGT	ABRELATERD1	9.30E-04
	Bi-4	CTTCCTCTTC	NODCON2GM	4.10E-03
salt	Salt-1	CCGCCGCCGCCC	GCCCORE	3.50E-22
	Salt-2	AAAAAAAAAAAA	MARTBOX	7.30E-09
	Salt-3	AGAGAGAGGGG	None	5.20E-04
ABA	ABA-1	GGGGAGGGGGAG	None	3.00E-14
	ABA-2	AAAAAAAAGAAA	DOFCOREZM	1.30E-14
Drought	Drought-1	GGCGGAGGGGGG	None	7.60E-19
	Drought-2	AAAAAAAAAAA	MARTBOX	5.40E-08
	Drought-3	CCCTCTCTCTCT	CTRMCAMV35S	2.40E-05
